# CPX Targeting DJ-1 Triggers ROS-induced Cell Death and Protective Autophagy in Colorectal Cancer

**DOI:** 10.7150/thno.34663

**Published:** 2019-07-28

**Authors:** Jing Zhou, Lu Zhang, Meng Wang, Li Zhou, Xuping Feng, Linli Yu, Jiang Lan, Wei Gao, Chundong Zhang, Youquan Bu, Canhua Huang, Haiyuan Zhang, Yunlong Lei

**Affiliations:** 1 Department of Biochemistry and Molecular Biology, Molecular Medicine and Cancer Research Center, Chongqing Medical University, Chongqing, 400016, P.R.China; 2 State Key Laboratory of Biotherapy and Cancer Center, West China Hospital, and West China School of Basic Medical Sciences & Forensic Medicine, Sichuan University, and Collaborative Innovation Center for Biotherapy, Chengdu, 610041, P.R. China; 3 Key Laboratory of Tropical Diseases and Translational Medicine of Ministry of Education & Department of Neurology, the First Affiliated Hospital of Hainan Medical University, Haikou, P. R. China

**Keywords:** Ciclopirox olamine, DJ-1, mitochondria, ROS, Autophagy

## Abstract

**Rationale:** Colorectal cancer (CRC) is one of the most common cancers worldwide. Ciclopirox olamine (CPX) has recently been identified to be a promising anticancer candidate; however, novel activities and detailed mechanisms remain to be uncovered.

**Methods:** The cytotoxic potential of CPX towards CRC cells was examined *in vitro* and *in vivo*. The global gene expression pattern, ROS levels, mitochondrial function, autophagy, apoptosis, etc. were determined between control and CPX-treated CRC cells.

**Results:** We found that CPX inhibited CRC growth by inhibiting proliferation and inducing apoptosis both *in vitro* and *in vivo*. The anti-cancer effects of CPX involved the downregulation of DJ-1, and overexpression of DJ-1 could reverse the cytotoxic effect of CPX on CRC cells. The loss of DJ-1 resulted in mitochondrial dysfunction and ROS accumulation, thus leading to CRC growth inhibition. The cytoprotective autophagy was provoked simultaneously, and blocking autophagy pharmacologically or genetically could further enhance the anti-cancer efficacy of CPX.

**Conclusion:** Our study demonstrates that DJ-1 loss-induced ROS accumulation plays a pivotal role in CPX-mediated CRC inhibition, providing a further understanding for CRC treatment via modulating compensatory protective autophagy.

## Introduction

Colorectal cancer (CRC) is one of the most commonly diagnosed cancers among both men and women worldwide, with 8.1% of all new cancer cases and 8.3% of all cancer deaths in 2018. Although over the past several decades historical changes in risk factors (eg. smoking, increased use of aspirin, red and processed meat consumption, obesity, and diabetes) and the dissemination of colonoscopy with polypectomy have decreased the incidence and mortality rates 1, almost 50% of the patients after surgical resection have a risk of recurrence and metastasis leading to death within 5 years of diagnosis 2. Randomized trials have shown that the death and recurrence in patients treated with adjuvant chemotherapy (for patients with stage III/IV and high-risk stage II colon cancer) have been reduced 3, 4. While the lack of informative markers for the identification of patients at high risk of relapse who might benefit from adjuvant therapy, the effectiveness of chemotherapy has often been limited 5, 6. Hence at present, searching for novel target-based therapeutic agents for colorectal cancer is an urgent need.

Ciclopirox olamine (CPX), a synthetic antifungal agent and iron chelator used to treat mycoses of the skin and nails for more than 20 years, has recently been identified as a promising antitumor candidate 7, 8. Most recent studies have revealed that CPX induced cell death in many types of tumor, including acute myeloid leukemia (AML), breast cancer, colorectal cancer, neuroblastoma, and inhibited tumor growth in primary AML cells xenografts as well as human breast cancer MDA-MB-231 xenografts without gross organ toxicity or loss of body weight 9-13. CPX was capable to inhibit proliferation and induce apoptosis through regulating expression of cell cycle and apoptosis-regulating proteins in breast cancer 10, 11, 14. Moreover, CPX could induce autophagy by ROS-mediated JNK activation^15^. Notably, previous human phase I study of oral CPX has evaluated its safety, dose tolerance, pharmacokinetics, and pharmacodynamics in patients with refractory hematologic malignancies, demonstrated that CPX was rapidly absorbed and revealed a favorable therapeutic index of CPX, but affected by a short half-life 16. However, the detailed molecular mechanisms underlying CPX-mediated suppression of tumor growth remain to be further elucidated.

DJ-1 (RS/PARK7/CAP1) is a highly conserved homodimeric protein which was initially cloned as a putative oncogene capable of transforming NIH-3T3 cells in cooperation with H-Ras 17. The Leu166Pro (L166P) mutation and degradation of DJ-1 is known to be linked with early-onset, autosomal recessive Parkinson's disease (PD) and thus DJ-1 is thought to play a role in neuroprotection 18, 19. In addition, DJ-1 functions primarily as an endogenous antioxidant and protects cells from oxidative injury through modulating many signal transduction, such as extracellular signal-regulated kinase (ERK) 1/2, apoptosis signal-regulating kinase (ASK) 1, Akt/mTOR, and HIF1α signaling pathways 17, 20, 21. Several lines of evidence subsequently suggest that DJ-1 is over-expressed in multiple types of tumor and is positively correlated with tumor progression, tumor recurrence, and chemotherapeutic drug resistance 22. In our previous study, we have demonstrated that DJ-1 promotes CRC proliferation and metastasis and is negatively correlated with patient survival 23. Therefore, DJ-1 expression level may be utilized for identifying CRC patients that are more likely to relapse and those who may benefit from more aggressive adjuvant chemotherapeutic treatment.

Autophagy, a lysosome-dependent catabolic pathway by which damaged or senescent organelles are removed, plays an important role in the regulation of cancer progression and in determining the response of tumor cells to anti-cancer therapy. Four functional forms of autophagy have been described to date: the cytostatic, nonprotective, cytotoxic and cytoprotective autophagy 24, of which cytoprotective autophagy is more frequent in response to chemotherapy. A growing body of evidence implicates that autophagy can enhance the acquired resistance of some cancer cells during chemotherapy resulting in limited effectiveness of anti-cancer drugs 25, 26, suggesting autophagy as a potential target for cancer treatment.

In this work, we find that CPX induces apoptosis and growth inhibition in CRC cells via massive accumulation of ROS. CPX promotes transcriptional-mediated downregulation of DJ-1, a potential biomarker for CRC diagnosis and prognosis, which results in the mitochondrial dysfunction, and thereby increases the levels of ROS. Concomitantly, the accumulation of ROS induces cytoprotective autophagy in response to CPX treatment, thus impairs the antitumor effect of CPX. CPX treatment accompanied with autophagy inhibition can effectively inhibit CRC growth. Collectively, our results demonstrate an experimental foundation for downregulating DJ-1 as an important mechanism underlying CPX anticancer activity and provide evidence for targeting cytoprotective autophagy as a potential chemotherapeutic strategy.

## Material and methods

### Cell culture

The HIEC, SW480, HT29, DLD-1 and SW620 cell lines were purchased from the American Type Culture Collection (ATCC), HCT116 and RKO cell lines were purchased from Shanghai cell bank, NCM460 was purchased from In Cell. All cell lines were cultured according to the guidelines and were maintained in DMEM(Gibco) supplemented with 100 U/mL penicillin (Sigma), 10 mg/L streptomycin (Sigma), and 10% serum (Hyclone) in a humidified incubator at 37 ℃ under 5% CO_2_ atmosphere.

### Reagents and antibodies

The following primary antibodies were used: DJ-1 (Santa Cruz Biotechnology), RUNX1 (Santa Cruz Biotechnology), Lamp1 (Santa Cruz Biotechnology), LC3 (MBL International Corporation), p62 (MBL International Corporation), PARP (Cell Signaling Technology), PRKAA/AMPK (Cell Signaling Technology), phosphor-PRKAA/AMPK (Cell Signaling Technology), Beclin1 (Cell Signaling Technology), ATG5 (Cell Signaling Technology), PCNA (abcam), NFIL3 (Santa Cruz Biotechnology), ETS1 (Santa Cruz Biotechnology), FOXA1 (Santa Cruz Biotechnology), C-JUN (Santa Cruz Biotechnology), EGR3 (Santa Cruz Biotechnology), ATF3 (Santa Cruz Biotechnology), MAFF3 (Santa Cruz Biotechnology), BHLHE40 (Santa Cruz Biotechnology), NR4A2 (Santa Cruz Biotechnology). Ciclopirox olamine, Rotenone, APO, NDGA, NAC, Glucose, Oligomycin, 2-DG, CQ, Bafilomycin A1, E64D and PepA were purchased from Sigma.

### Animal models

All studies were approved by the Institutional Animal Care and Treatment Committee of Sichuan University. For establishment of tumor xenografts, DJ-1-overexpressing and vector HCT116 cells were stably transfected using pReceiver-Lv103 lentivirus vector containing puromycin for screening. 1×10^7^ cells were suspended in PBS and injected subcutaneously into 5-week-old female Balb/c mice. When the tumor volumes reached 100 mm^3^, mice were randomized into two groups, respectively, treated with CPX (25 mg/kg) prepared in a solution (4% ethanol) or vehicle control via oral gavage once daily. Mice were euthanized for analysis after three weeks. Tumor tissues were isolated and frozen in liquid nitrogen or fixed in 10% formalin immediately.

### Quantitative RT-PCR (qRT-PCR)

Total RNA was extracted using Trizol reagent (Invitrogen) and reverse transcribed using Reverse Transcription PrimeScript 1st Stand cDNA Synthesis kit (TaKaRa, Otsu, Japan). qRT-PCR were performed using quantitative PCR reagents SYBR PremixEx TaqTM (TaKaRa) following the manufacturer's instructions. Levels of GAPDH quantified with target genes acts as an internal control and fold-changes were analyzed using the 2^-ΔΔCt^ method. The qRT-PCR primer sequences were shown in Supplementary Table 2.

### Luciferase reporter constructs and reporter assays

The DJ-1 promoter region (from 1 kb upstream of transcription start site to 1 kb downstream) were cloned into pGL3-luciferase reporter vector (Promega). The PCR primers for DJ-1 promoter were as follows: Forward primer: GCGTGCTAGCCCGGGCTCGAGGGATCCTTCTAAGCTCATTCAA, Reverse primer: CAGTACCGGAATGCCAAGCTTGAGCTCTTTTGGAAGCCAT.

For luciferase reporter analysis, HCT116 cells were cotransfected with pRL-TK vector (Promega) encoding Renilla luciferase, RUNX1 expression vector, and pGL3-Basic or DJ-1-reporter construct, respectively. After 24 hours, cells were treated with or without CPX at the indicated concentrations for 24 hours, then cells were lysed and luciferase activity was measured using a Dual Luciferase Assay System (Promega) according to the manufacturer's instructions. All transfection experiments were performed 3 to 4 times in triplicate.

### Mitochondrial and glycolysis stress tests

Oxygen consumption rate (OCR) and extracellular acidification rate (ECAR) were measured using the Seahorse XF24 analyzer. Briefly, HCT116 and SW480 cells were seeded in plates after 24-hour CPX treatment. After 12 hours, the cells washed three times using seahorse assay medium and then loaded into the machine. Repeat measurement cycles (3 minutes Mix, 2 minutes Wait, 3 minutes Measure) three times for Basal OCR and ECAR after injection of 10 mM glucose, 1 μM oligomycin and 50 mM 2-deoxyglucose.

### Immunofluorescence

Cells grown on coverslips that treated with CPX for 24 hours were fixed with 4% paraformaldehyde (Sigma) for 30 minutes and then washed three times with PBS. Fixed cells were permeabilized with 0.4% Triton 100 and 5% BSA for 1 hour at 37 ℃. For staining, cells were incubated with primary antibodies for 12 hours at 4 ℃, followed by incubation with secondary antibodies (Thermo Scientific) for 1 hour at 37 ℃. Finally, Nuclei were stained with DAPI for 10 minutes. For autophagy flux studies, cells were transfected with GFP-RFP-LC3 for 24 hours and then treated with CPX for another 24 hours. Images were captured using a confocal microscopy (Carl Zeiss MicroImaging).

### Immunohistochemistry

Immunohistochemical (IHC) staining was performed as described previously 27. After blocking with goat serum for 1 hour, the sections were then probed with the desired primary antibody, including DJ-1, p-AMPK, LC3, and PCNA. The immunostaining intensity (A) was divided into four grades (0, negative; 1, weakly positive; 2, positive; 3, strongly positive), and the proportion of staining-positive cells (B) was indicated by five grades (0, <5%; 1, 6-25%; 2, 26-50%; 3, 51-75%; 4, >75%). The final score for each slide was calculated as ΣA*B.

### Cell viability and proliferation assays

CCK8 assay was performed to determine cell viability. Briefly, cells were seeded in 96-well plates at a density of 5000 cells. After treatment, CCK8 (Dojindo, Kamimashiki-gun, Kumamoto, Japan; CK04) were added and incubated for 40 minutes. The absorbance value was then determined at 450 nm.

The long-term effects of CPX on CRC cell proliferation were analyzed with a colony formation assay. After treatment, 1500-2000 cells were seeded in six-well plates and the medium was changed every 3 days. After two weeks, cells were fixed with 4% paraformaldehyde (Sigma) for 30 minutes and stained with Crystal Violet for another 30 minutes, and then the colonies were washed three times and taken photos.

Cell proliferations were determined using EDU assay. Cells grown on coverslips were treated with CPX for 24 hours and stained with EDU (RiBoBio) for 12 hours. Then, operating according to the instructions of the manufacturer. Finally, Nuclei were stained with DAPI for 10 minutes and observed using fluorescence microscopy (Leica).

### Immunoblot and immunoprecipitation

Cells were lysed with RIPA buffer (50 mM Tris base, 1.0 mM EDTA, 150 mM NaCl, 0.1% SDS, 1% Triton X-100, 1% sodium deoxycholate, 1 mM PMSF) supplemented with protease inhibitor cocktail (Sigma) and then protein lysates were centrifuged and boiled with loading buffer. For immunoprecipitation, Whole cell lysates were prepared in RIPA buffer (40 mM TRIS-HCl, pH 7.5, 150 mM NaCl, 0.5% Nonidet P-40, cocktail, 5% glycerol, 10 mM NaF) and incubated with 1 μg of antibody overnight at 4 °C. Next day, Sepharose protein A/protein G beads were added for 2 hours. The immune-complexes were then centrifuged and washed 3 times using RIPA buffer. All lysates were quantified by the BCA Protein Assay (Thermo Fisher Scientific) and analyzed by SDS-PAGE.

### Acridine orange staining

To detect the formation of acidic vesicular organelles (AVO), cells were seeded in 24-well plates and treated with or without CPX at indicated concentration for 24 hours, and then stained with 1 µmol/L acridine orange (Signa-Aldrich) for 15 minutes. Finally, Cells were washed five times and then observed using fluorescence microscopy (Leica).

### Transfection

All siRNAs were designed using BLOCK-iT™ RNAi Designer (Invitrogen) and synthesized by GenePharma (Shanghai, China). The sequences of the siRNAs used are listed in Supplementary Table 3. Cells were transfected with siRNAs using Lipofectamine RNAiMax (Invitrogen) according to the manufacturer's instruction.

pCMV-myc-DJ-1 and Dominant-negative PRKAA1 (DN-PRKAA1/DN-AMPKα1) plasmids were obtained as described previously 23, 28. The plasmids were transfected into the indicated cells using Lipofectamine 2000 (Invitrogen) according to the manufacturer's instruction.

### Transmission electron microscopy

Transmission electron microscopy assay was used to visualize autophagic vesicles and mitochondrial morphology. After 24-hour CPX treatment, HCT116 cells were fixed in glutaraldehyde (Sigma) and ultrathin sections were prepared using a sorvall MT5000 microtome. Then, the sections were stained by lead citrate and /or 1% uranyl acetate and visualized by Philips EM420 electron microscopy.

### Flow cytometry

The indicated cells treated with CPX for 24 hours were harvested and washed three times with PBS, then resuspended and incubated with Annexin V/PI solution (KeyGEN Biotech). Apoptosis was analyzed with a FACSCalibur flow cytometer (Becton Dickinson, San Jose, CA USA).

### Chromatin immunoprecipitation (ChIP)

ChIP was performed with the EZ ChIP™ Chromatin Immunoprecipitation kit (catalog # 17-371) according to the manufacturer's instructions. Anti-RUNX1 (Santa Cruz Biotechnology) was used for ChIP. The qRT-PCR primer sequences were shown in Supplementary Table 4. ChIP qPCR data are calculated relative to IgG using the 2^-ΔΔCt^ method, where ΔCt = (Ct [ChIP]-(Ct [Input]-Log2^100^)). The experiments were repeated at least three times.

### Data analysis and statistics

Data were expressed as means ± s.d. All experiments were performed at least three times. Statistical analysis was performed with GraphPad Prism 6.0 software. Statistical differences between groups were determined using two-tailed Student's t-test. Significance was designated as follows: *, P < 0.05, **, P < 0.01, ***, P < 0.001.

## Results

### CPX inhibits proliferation and induces apoptosis in CRC cell

**Figure 1:**
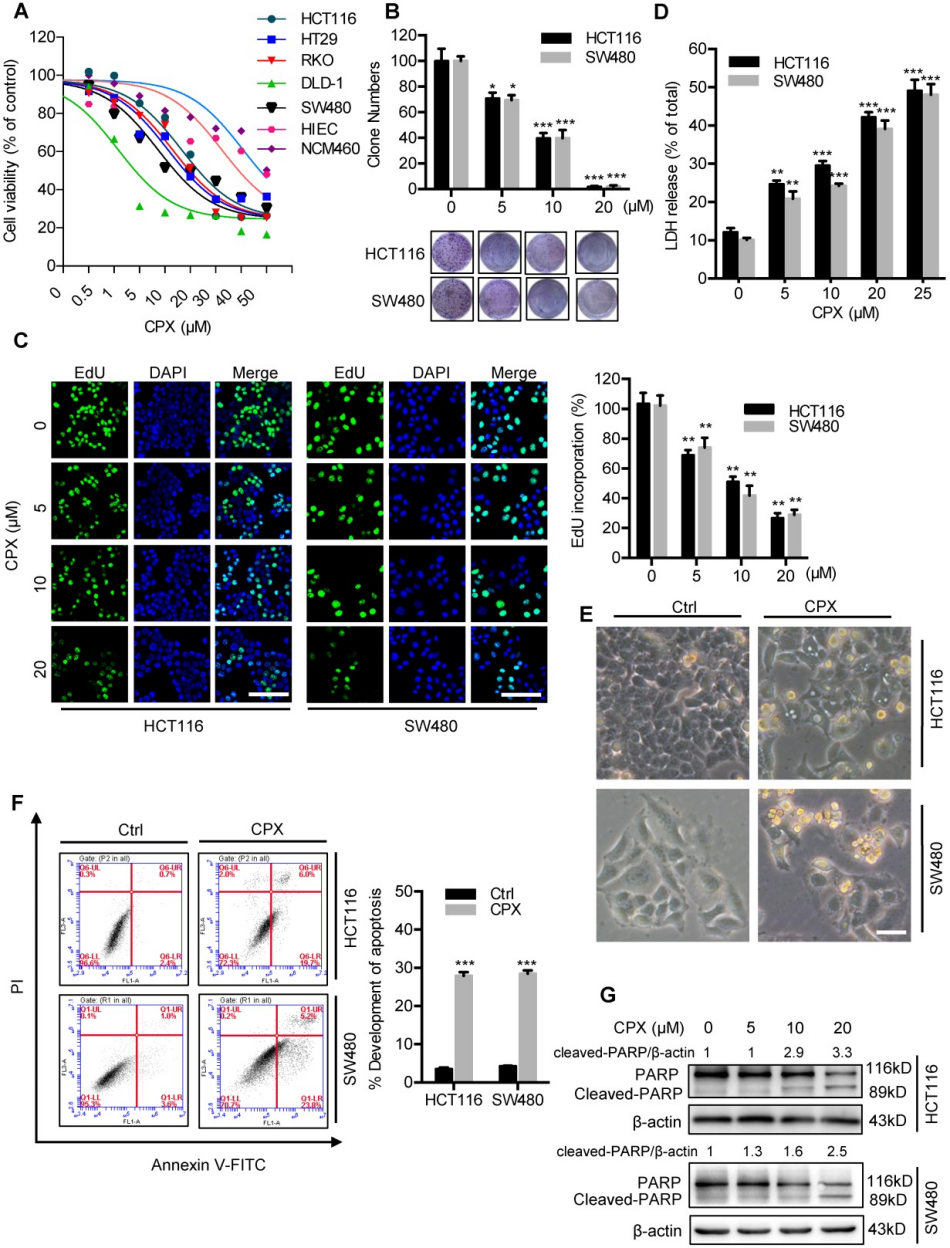
** CPX inhibits proliferation and induces apoptosis in CRC cells. A.** HCT116, DLD-1, RKO, HT29, SW480, HIEC and NCM460 cells were treated with the indicated concentrations of CPX for 24 hours and cell viability was determined by CCK8 kit. **B.** Cell proliferation rate was analyzed by clone formation assay. HCT116 and SW480 cells were treated with the indicated concentration of CPX for 24 hours, after treatment, cells were seeded into 6-well plates for two weeks and colony numbers were quantified. **C.** EdU assay of CRC cells treated with the indicated concentration of CPX for 24 hours. The EdU incorporation was quantitated. Scale bar, 100 μm. **D.** Analysis of dehydrogenase (LDH) release in HCT116 and SW480 cells subjected to indicated concentration of CPX for 24 hours. **E.** Morphology of HCT116 and SW480 cells following treatment with or without 20 μM CPX for 24 hours. Scale bar, 100 μm.** F.** HCT116 and SW480 cells treated with 20 μM CPX for 24 hours were fixed, stained with Annexin V/PI, and then analyzed by flow cytometry. The apoptosis rate was quantitated. **G.** Immunoblot analysis of PARP in HCT116 and SW480 cells. Data are means ± s.d. and are representative of 3 independent experiments. *, P < 0.05, **, P < 0.01, ***, P < 0.001. Statistical significance compared with respective control groups.

To validate whether CPX exhibited antitumor effect against CRC, CCK8 assay was performed to assess the cell viability in response to CPX treatment in different human CRC cell lines. As shown in Fig. 1A and Fig S1A, CPX treatment for 24 hours markedly decreased the cell viability of various CRC cell lines (HCT116, DLD-1, RKO, HT29, and SW480) with relatively low IC50 values, whereas normal human small intestine or colon mucosal epithelial cell lines HIEC and NCM460 cells demonstrated higher tolerance to CPX. Consistently, the proliferation of CRC cells was significantly inhibited under CPX treatment, as evidenced by reduced colony formation and EdU incorporation (Fig. 1B, 1C, and Fig S1B). We then performed LDH (lactate dehydrogenase) release assay and found that CPX treatment exhibited marked cytotoxicity in HCT116 and SW480 cells (Fig. 1D). Previous studies have suggested that CPX exerts antitumor activity by inducing apoptosis 10. To evaluate whether apoptosis was involved in CPX-induced cytotoxicity in CRC cells, we first detected morphology of CRC cells and found obvious cytoplasmic vacuole formation and morphological changes that are typical of apoptosis in CPX-treated CRC cells (Fig. 1E and Fig. S1C). The pro-apoptotic effect of CPX in HCT116, SW480 and HIEC cells was further evidenced by Annexin V/PI staining measured with flow cytometry (Fig. 1F and Fig. S1D). In addition, CPX treatment resulted in increased levels of cleaved PARP, a specific and sensitive marker of apoptosis, in CRC cells (Fig. 1G). Collectively, these results demonstrate that CPX exhibits a considerable antitumor effect in CRC cells *in vitro*.

### CPX-induced downregulation of DJ-1 is involved in anti-CRC effects of CPX

**Figure 2:**
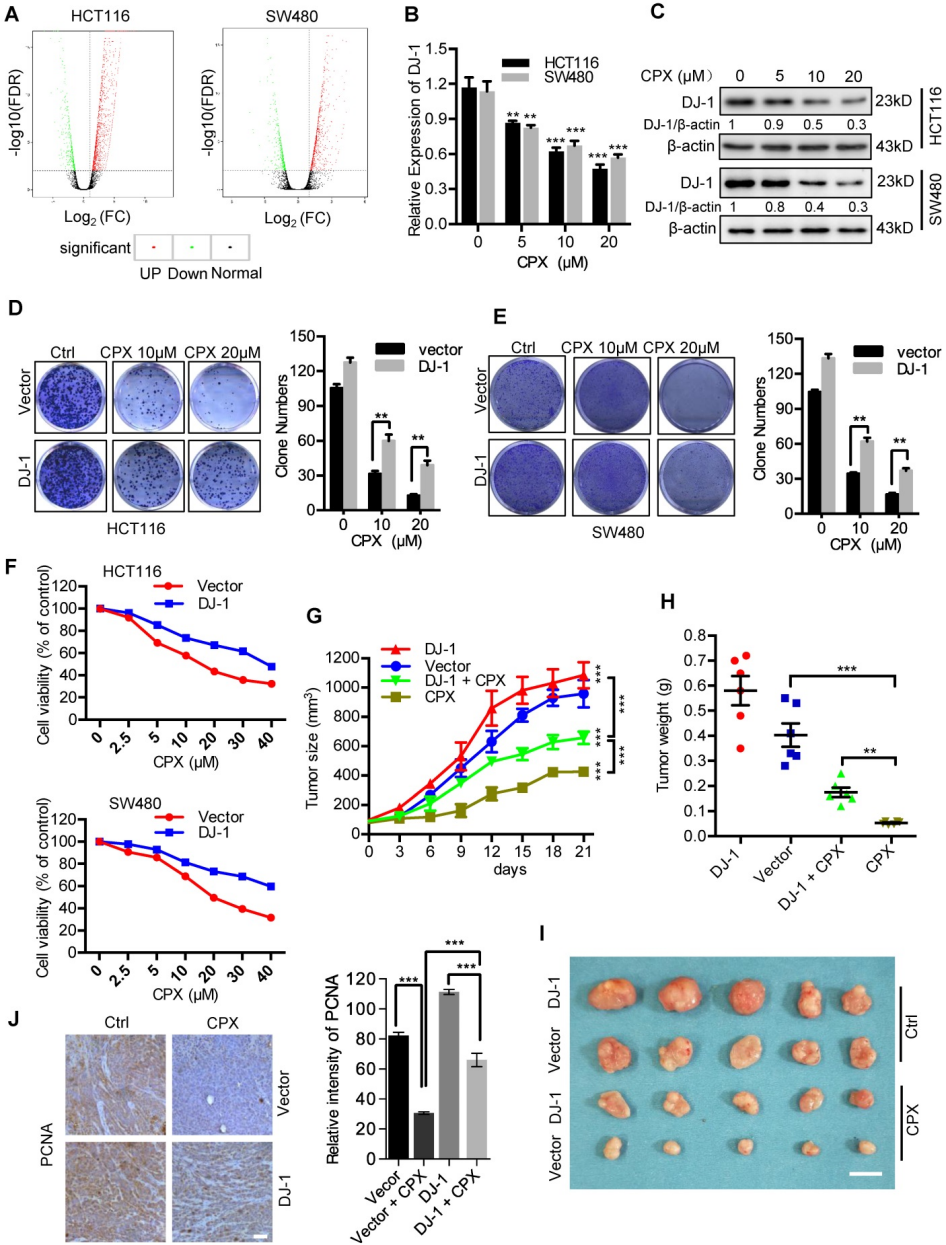
** CPX-induced downregulation of DJ-1 is involved in anti-CRC effects of CPX. A.** Volcano plot analysis of DEGs in HCT116 and SW480 cells treated with or without 20 μM CPX for 24 hours. Red areas indicate significant overexpression and green areas indicate significant downregulation. **B.** qRT**-**PCR was conducted in HCT116 and SW480 cells treated with or without 20 μM CPX for 24 hours to determine DJ-1 mRNA expression. Statistical significance compared with respective control groups. **C.** Immunoblot analysis of DJ-1 expression in CRC cells treated with or without the indicated concentration of CPX for 24 hours. **D** and **E.** Colony formation assay of CRC cells stably overexpressing DJ-1 and Vector treated with indicated concentrations of CPX.** F.** Cell viability assay was performed by CCK8 kit. **G-J.** HCT116 cells stably overexpressing DJ-1 and Vector were subcutaneously injected into nude mice(n=6). Tumor volumes were monitored at indicated time points (G), and the weight of tumors were measured at time of sacrificed (H). (I) The image of isolated tumors derived from mice treated with vehicle or CPX (25 mg/kg/day). (J) Representative images of immunohistochemical staining of PCNA and quantification of relative intensity of PCNA staining in xenografts. Scale bar, 100 μm. *, P < 0.05, **, P < 0.01, ***, P < 0.001.

To demonstrate the molecular mechanisms of CPX-induced CRC suppression, we determined the global gene expression pattern for CPX-treated cells and compared it with that for the controls using RNA-seq. Using a ≥ 2-fold change (FC) and < 0.05 P-value as a cut-off to define overexpression or downregulation, we identified a series of differential expressed genes (DEGs) when compared between CPX-treated and control groups. Sequentially, we divided these DEGs into 843 upregulated genes and 454 downregulated genes in HCT116 cells (Fig. 2A and Supplementary Table 1) and 1651 upregulated genes and 449 downregulated genes in SW480 cells (Fig. 2A). Notably, the expression of DJ-1 was markedly decreased in CPX-treated CRC cells. We then performed the real-time PCR to ensure the expression levels of DJ-1 under CPX treatment. As shown in Fig. 2B, CPX treatment decreased the levels of DJ-1 mRNA in a dose-dependent manner. This was also confirmed by immunoblot analysis of DJ-1 protein levels in CPX-treated CRC cells (Fig. 2C).

To investigate whether CPX-induced downregulation of DJ-1 was involved in CRC suppression, we evaluated cell viability and proliferation in CRC cells stably overexpressing DJ-1 under CPX treatment. As shown in Fig. 2D and 2E, DJ-1 overexpression markedly restored CPX-induced suppression of cell proliferation in HCT116 and SW480 cells. Consistently, the cell viability of DJ-1 overexpression cells was also higher than that of control cells when treated with CPX (Fig. 2F). We then generated a mouse xenograft model by subcutaneously inoculating human CRC HCT116 cells into nude mice. Consistent with *in vitro* study, exogenous expression of DJ-1 could obviously impair the anti-CRC effects of CPX *in vivo*, as evidenced by faster growth rate and larger size of tumors (Fig. 2G-2I). This was further supported by stronger PCNA intensity in xenografts of DJ-1 overexpression group than those of control group when treated with CPX (Fig. 2J). Taken all, these data suggest that CPX-induced downregulation of DJ-1 is involved in anti-CRC effects of CPX.

### ROS induced by DJ-1 downregulation are responsible for anti-CRC effects of CPX

**Figure 3:**
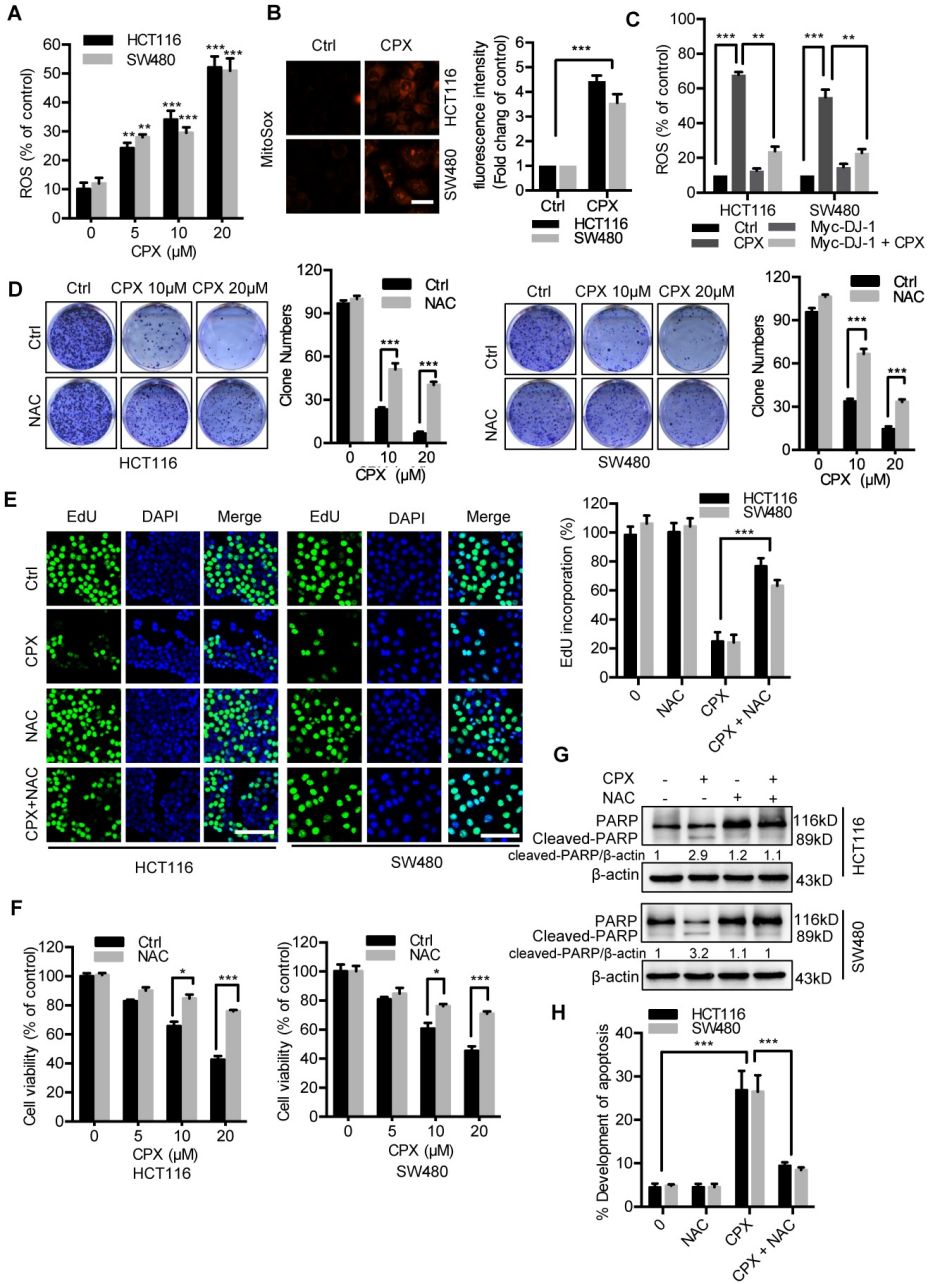
** ROS induced by DJ-1 downregulation are responsible for anti-CRC effects of CPX. A.** ROS level was analyzed by DCFH-DA staining via a fluorescence microplate Reader (Thermo scientific) in CRC cells treated with the indicated concentrations of CPX. Statistical significance compared with respective control groups. **B.** MitoSOX Red was used to evaluate ROS level in mitochondria upon CPX treatment. Scale bar, 25μm. **C.** ROS level was determined by flow cytometry in CRC cells treated with or without 20 μM CPX for 24 hours overexpressing Myc-DJ-1 and Vector. **D.** Colony formation assay of CRC cells treated with the indicated concentrations of CPX in the presence or absence of 5mM NAC. **E.** EdU assay of CRC cells treated with or without 5mM NAC in the presence or absence of 20 μM CPX for 24 hours. The EdU incorporation was quantitated. Scale bar, 100 μm. **F.** The CCK8 assay determined cell viability of CRC cells treated with the indicated concentrations of CPX in the presence or absence of 5mM NAC for 24 hours. **G.** Immunoblot analysis of PARP expression in HCT116 and SW480 cells treated with CPX (20 μM) or vehicle and/or with NAC (5mM).** H.** HCT116 and SW480 cells treated with CPX (20 μM) or vehicle and/or with NAC (5mM) were fixed, stained with Annexin V/PI, and then analyzed by flow cytometry. The development of apoptosis was quantitated. Data are means ± s.d. and are representative of 3 independent experiments. *, P < 0.05, **, P < 0.01, ***, P < 0.001.

Previous studies have demonstrated that decreased DJ-1 leads to impaired antioxidant response 29. Therefore, we determined to detect whether CPX-induced DJ-1 downregulation could increase cellular ROS levels. Treatment of CPX dramatically increased cellular ROS levels (Fig. 3A and 3B), whereas exogenous expression of DJ-1 could reverse this phenotype in HCT116 and SW480 cells (Fig. 3C). Next, we would like to identify the role of ROS in CPX-induced growth suppression. We found that combinatorial treatment of CPX with ROS scavenger N-Acetyl-cysteine (NAC) restored CPX-induced growth suppression, as evidenced by increased colony formation, EdU incorporation and cell viability in combinatorial treatment cells (Fig. 3D-3F). In addition, treatment of NAC also reduced the pro-apoptotic effect of CPX, as evidenced by Annexin V/PI staining and levels of cleaved PARP (Fig. 3G and 3H). Together, these findings reveal that CPX downregulates DJ-1 and induces cellular ROS accumulation, thereby promoting apoptotic cell death.

### CPX induces mitochondrial dysfunction to promote ROS accumulation in CRC cells

**Figure 4:**
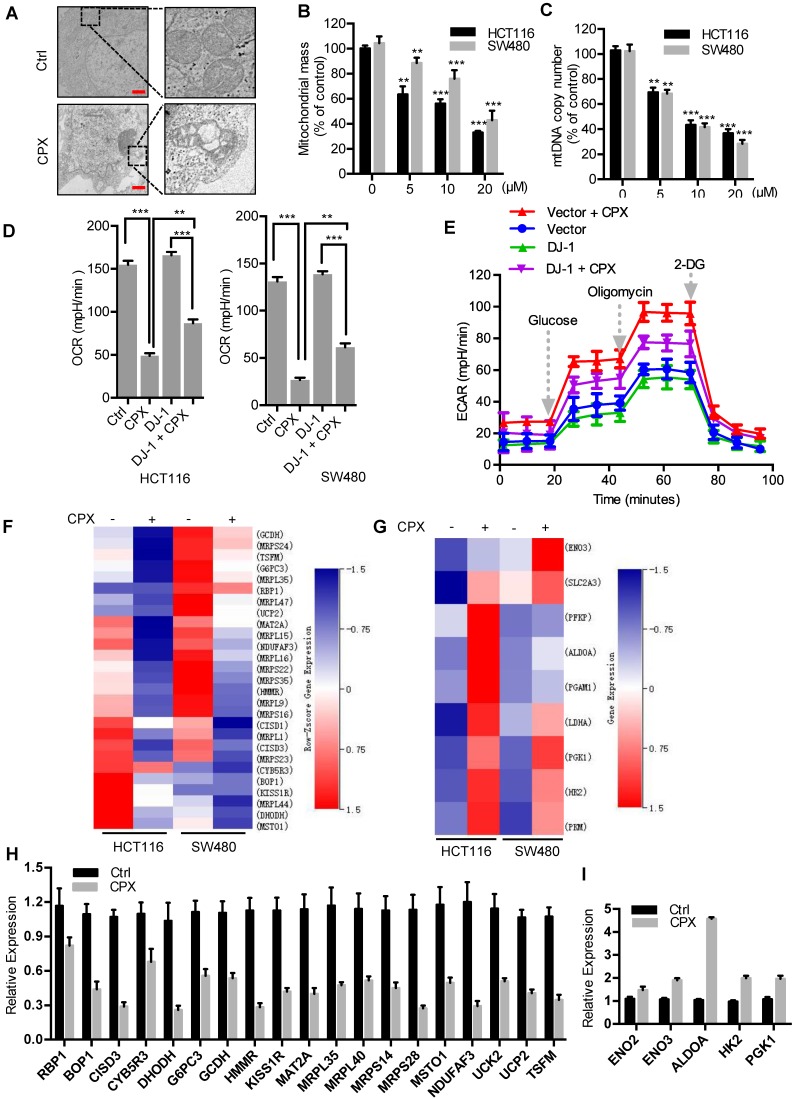
** CPX induces mitochondrial dysfunction to promote ROS accumulation in CRC cells. A.** Transmission electron microscopy images from HCT116 cells treated with or without 20 μM CPX. Scale bar, 1 μm.** B.** Mitotracker Green was used to analyze mitochondrial mass independent of mitochondrial membrane potential (MMP) alteration of CRC cells treated with or without 20 μM CPX for 24 hours via a fluorescence microplate Reader (Thermo scientific). Statistical significance compared with respective control groups. **C.** The number of mitochondrial DNA copies was examined by qRT-PCR in HCT116 and SW480 cells treated with or without 20 μM CPX for 24 hours. Statistical significance compared with respective control groups. **D.** CRC cells were transfected with empty Vector or Myc-DJ-1 plasmid for 24 hours, followed by treatment with or without 20 μM CPX for 24 hours, then seeded in plates for 12 hours before OCR analysis. Basal OCR was analyzed under XF Base Medium containing 10 mM glucose, 1 mM sodium pyruvate and 2 mM glutamine (pH was adjusted to 7.40 ± 0.05 at 37°C) (n = 3 per group). **E.** The glycolysis stress test was performed under XF Base Medium containing 2 mM glutamine (pH was adjusted to 7.40 ± 0.05 at 37°C) after injection of 10 mM glucose, 1μM oligomycin (an inhibitor of mitochondrial membrane adenosine triphosphate synthase) and 50 mM 2-deoxyglucose (2-DG, a non-metabolizable glucose analog that inhibits glycolysis) in HCT116 cells treated as in (D) (n = 3 per group). **F** and **G.** Heat map shows normalized intensity values for genes involved in oxidative phosphorylation (F) and glycolysis assessment (G) after 20 μM CPX treatment with a P value < 0.05. **H** and **I.** qRT**-**PCR analysis the expression of genes involved in oxidative phosphorylation (H) and glycolysis (I) after 20 μM CPX treatment in HCT116 cells. This experiment was carried out in triplicate. Data are means ± s.d. *, P < 0.05, **, P < 0.01, ***, P < 0.001.

In the above study, we have found that CPX induces ROS accumulation and as shown in Fig. 3B, mitochondria, a major source of ROS production, produces more superoxide in CPX-treated CRC cells. To evaluate whether CPX induces mitochondrial alterations in CRC cells, we examined the morphology, quantity, and function of mitochondria. Compared with the normal tubular mitochondria in control group, CPX treatment led to apparent alteration in mitochondrial morphology or even damage (Fig. 4A). After CPX treatment, a significant reduction in mitochondria mass was observed (Fig. 4B). As the relative mitochondrial number can be predicted by quantifying the mitochondrial DNA (mtDNA) copy number, we measured the levels of mtDNA content and found that CPX treatment resulted in decreased mtDNA copy number (Fig. 4C). Next, we tested whether CPX was involved in regulating mitochondria function and measured the oxygen consumption rate (OCR) and extracellular acidification rate (ECAR) in real-time. The results revealed that CPX inhibited mitochondrial oxidative phosphorylation (OXPHOS) and activated glycolysis (Fig. 4D, 4E and Fig. S2F). In addition, we performed RNA-seq to analyze the gene expression pattern of OXPHOS and glycolysis pathway. As shown in Fig. 4F and 4G, panels of OXPHOS-related genes were downregulated upon CPX treatment, whereas several key genes involved in glycolysis were upregulated, which was confirmed by Quantitative RT-PCR assay (Fig. 4H and 4I). Collectively, these data suggest that CPX induces mitochondrial dysfunction to promote ROS accumulation in CRC cells.

We next investigated the role of DJ-1 in ROS production and mitochondrial function. In the absence of CPX, knockdown of DJ-1 with siRNA increased ROS levels, decreased mtDNA copy number and OXPHOS-related gene expression (Fig. S2A-D), indicating a mitochondrial protective function of DJ-1. Upon the treatment of CPX, overexpression of DJ-1 partially restored the expression of OXPHOS-related genes (Fig. S2E) and the CPX-induced OCR inhibition (Fig. 4D) and attenuated CPX-induced ECAR elevation (Fig. 4E). These results suggest that DJ-1 plays an important role in CPX-induced mitochondrial dysfunction.

### CPX induces autophagy in CRC cells

**Figure 5:**
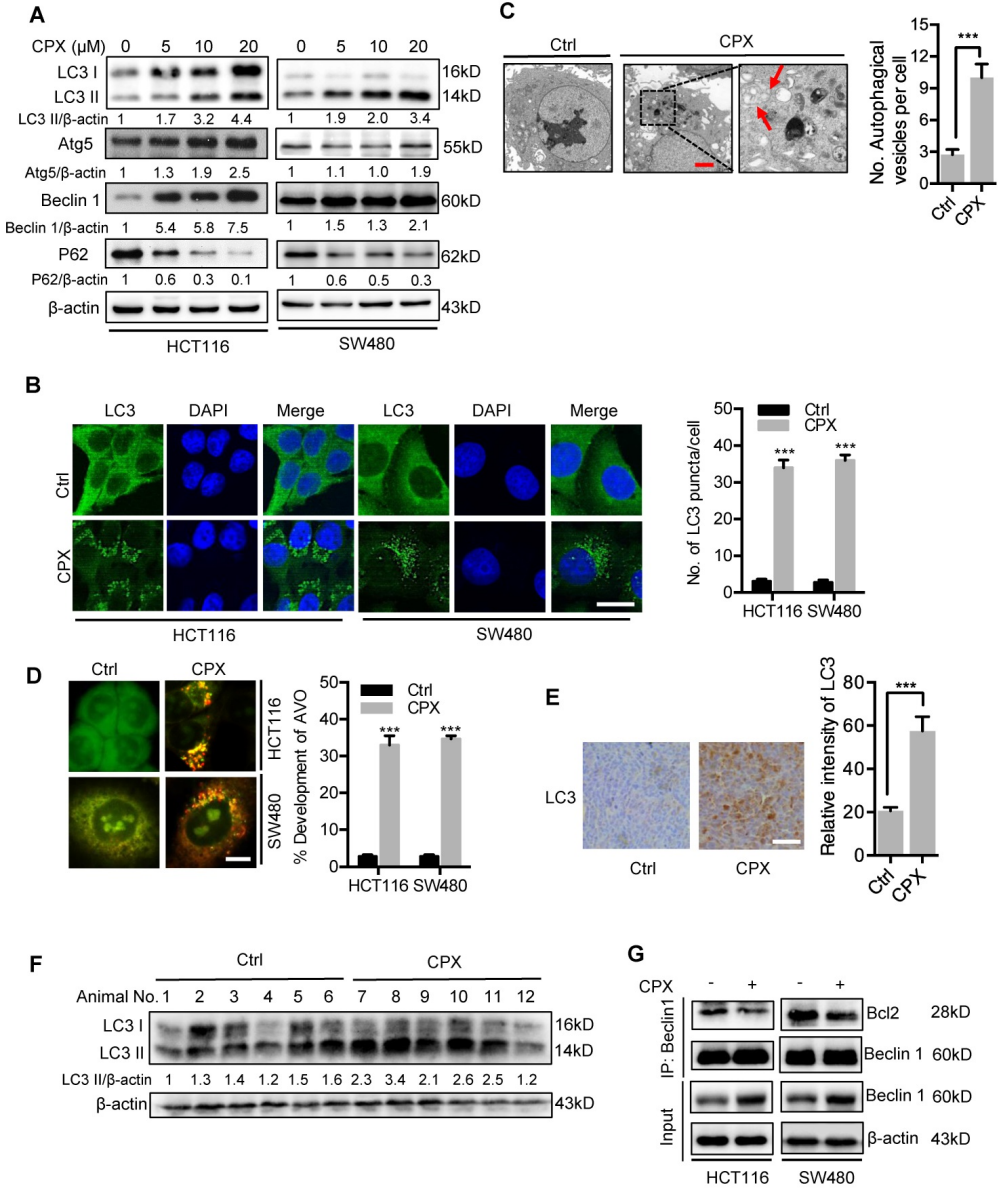
** CPX induces autophagy in CRC cells. A.** Immunoblot analysis of LC3, Atg5, Beclin1, and p62 expression in CRC cells treated with indicated concentrations of CPX for 24 hours. **B.** The formation of endogenous LC3 puncta was shown and quantitated by immunofluorescence analysis in cells treated with or without 20 μM CPX for 24 hours. Scale bar, 20 μm. **C.** Autophagic vesicles detected by transmission electron microscope in HCT116 cells treated as in (B). Scale bar, 2 μm.** D.** Left, autophagy was measured by acridine orange staining of cells treated as in (B). Right, total number of acidic vesicular organelles (AVO) per cell. Scale bar, 10 μm. **E.** Representative images of IHC analysis for LC3 expression in HCT116 xenografts collected from vehicle or CPX-treated mice. Scale bar, 50 μm. **F.** The protein expression level of LC3 in vehicle- or CPX-treated mice bearing HCT116 xenografts was examined by immunoblot analysis. **G.** Co-immunoprecipitation analysis of the interaction between Beclin1 and Bcl-2 in CRC cells treated with or without 20 μM CPX for 24 hours. Data are means ± s.d. *, P < 0.05, **, P < 0.01, ***, P < 0.001.

Increasing evidence has highlighted the potential application of pharmacologically modulating autophagy for cancer treatment 24. We thus investigated whether autophagy is regulated by CPX in CRC cells. To ascertain this hypothesis, we first examined the protein levels of autophagy-related genes in CPX-treated CRC cells. As a result, CPX treatment promoted the turnover of LC3-I to lipidated LC3-II, which is required for autophagosome formation, in a dose- and time-dependent manner in various CRC cells and other types of cancer cells (Fig. 5A and Fig. S3). The autophagic phenotype was further supported by the accumulation of autophagic vesicles (LC3 puncta) in CPX-treated cells compared with control cells (Fig. 5B). To further corroborate CPX-induced autophagy, the appearance of double-membraned autophagosomes was investigated by transmission electronic microscopy. As shown in Fig. 5C, there was a significant accumulation of autophagosomes/autolysosomes in CPX-treated cells but not in control cells. Cells were also stained with acridine orange to detect the formation of acidic vesicular organelles (AVO). As shown in Fig. 5D, abundant cytoplasmic AVO formation was readily observed in CPX-treated cells. In addition, mouse xenografts were stained with LC3 to clarify whether CPX could induce autophagy *in vivo*. As shown in Fig. 5E, CPX-treated xenografts displayed stronger LC3 staining compared with the control group. Consistently, a similar tendency was observed in LC3-II conversion in CPX-treated tumors (Fig. 5F).

Also, we examined the expression levels of Atg5 and Beclin 1, two autophagy-related proteins, to clarify whether CPX promoted the formation of autophagic vesicles. As shown in Fig. 5A, CPX enhanced the expression of Atg5 and Beclin 1 in a dose-dependent manner. In addition, it has been reported that the diminished interaction of Beclin 1 with Bcl-2 is a key event in the initiation process of autophagy 30. We found that CPX treatment decreased the binding of Beclin 1 with Bcl-2 (Fig. 5G). Moreover, silencing the expression of either Beclin 1 or Atg5 using siRNA prominently inhibited elevation of LC3 lipidation and endogenous LC3 puncta accumulation in CPX-treated cells (Fig. S4A-D). Co-administration of wortmannin, a PI3K inhibitor, with CPX failed to induce autophagy (Fig. S4E). Taken all, these data suggest that CPX induces autophagy in CRC cells both *in vitro* and *in vivo*.

### CPX promotes autophagy flux in CRC cells

**Figure 6:**
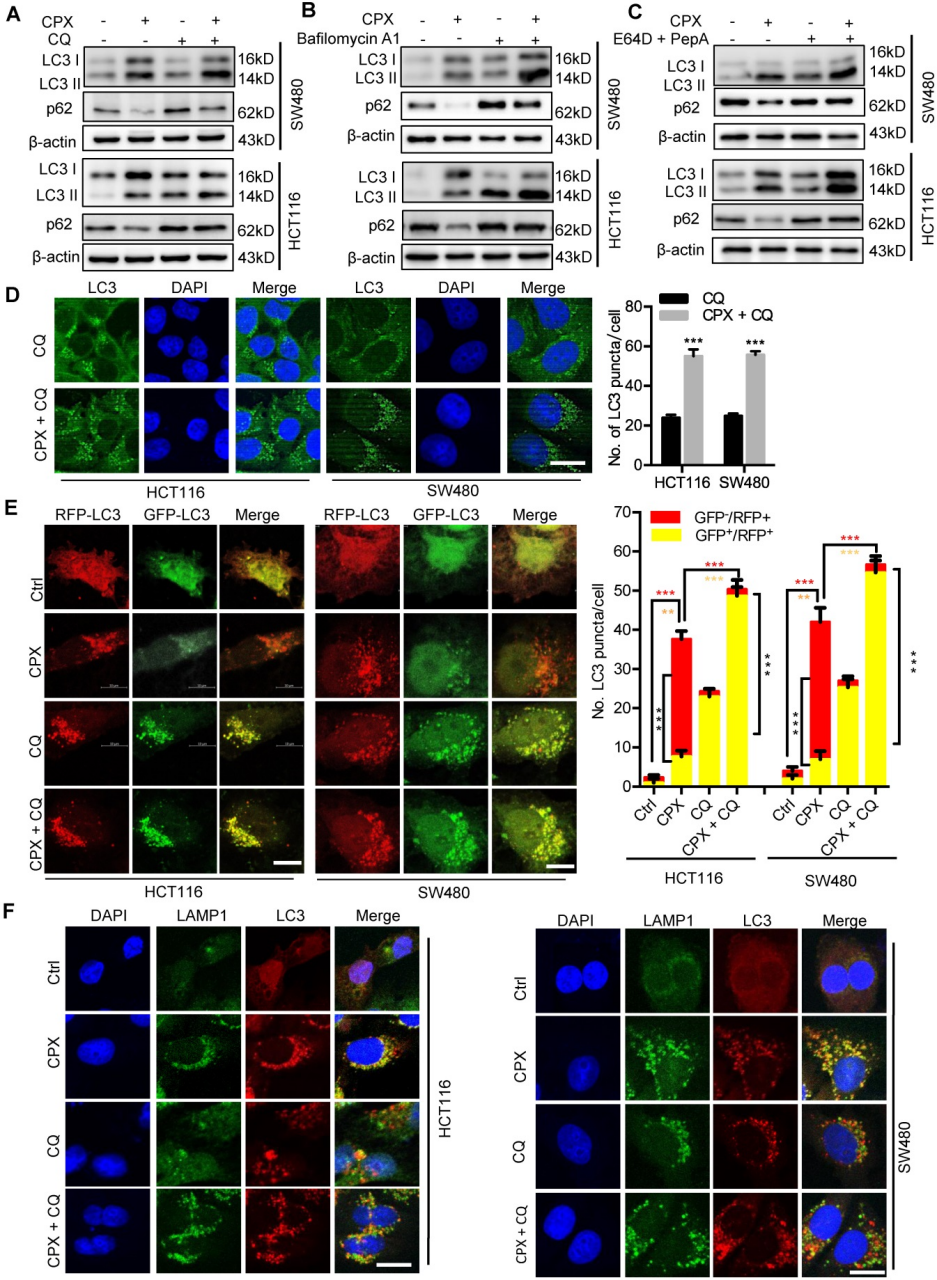
** CPX promotes autophagy flux in CRC cells. A.** CRC cells were treated with vehicle, CPX (20 μM), CQ (10 μM), or in combination for 24 hours. Immunoblot analysis was used to detect the expression of LC3 and p62.** B.** CRC cells were treated with CPX (20 μM) with or without Baf A1 (100 nM) for 24 hours. The expression of LC3 and p62 were examined by immunoblotting. **C.** CRC cells were treated with E64D (10μg/ml) and PepA (10μg/ml) in the presence or absence of CPX (20 μM) for 24 hours. The expression of LC3 and p62 were examined by immunoblotting. **D.** The accumulation of LC3 puncta was examined by immunofluorescent analysis of cells treated with CQ (10 μM) in the presence or absence of CPX (20 μM) for 24 hours. Scale bar, 20 μm. The number of LC3 puncta was quantitated. **E.** Immunofluorescence analysis of cells transiently transfected with tandem mRFP-GFP-tagged LC3 and treated with vehicle, CPX (20 μM), CQ (10 μM), or in combination for 24 hours. Scale bar, 10 μm. The ratio of red puncta indicating autolysosome (GFP^-^/RFP^+^) versus yellow puncta indicating autophagosome (GFP^+^/RFP^+^) was quantitated. **F.** Immunofluorescence analysis of the co-localization of endogenous LC3 and LAMP1 in CRC cells treated with vehicle, CPX (20 μM), CQ (10 μM), or in combination for 24 hours. Scale bar, 20 μm. Data are means ± s.d. and are representative of 3 independent experiments. *, P < 0.05, **, P < 0.01, ***, P < 0.001.

To determine whether CPX induced complete autophagic flux, we examined the protein levels of p62, a well-known autophagic substrate. As shown in Fig. 5A, we observed decreased p62 levels in CPX-treated cells along with the increased LC3-II levels, implying the induction of complete autophagic flux. In addition, combinatorial treatment of CPX with autolysosome inhibitors (CQ, Baf A1, or E64D and Pepstatin A) resulted in enhanced LC3-II turnover and accumulation of endogenous LC3 puncta (Fig. 6A-D and Fig. S5). Using a tandem monomeric RFP-GFP-tagged LC3, we found that CPX treatment resulted in dramatic formation of red fluorescent autophagosomes, which transformed into yellow under co-treatment with CQ (Fig. 6E). To further confirm the fusion of autophagosome with lysosome in CPX-treated CRC cells, we examined the colocalization of LC3 with LAMP1 (lysosome marker). CPX treatment induced obvious colocalization of LC3 with LAMP1, suggesting the fusion of autophagosome with lysosome (Fig. 6F). Together, these findings reveal that CPX induces complete autophagic flux in CRC cells.

### DJ-1/ROS/AMPK axis contributes to CPX-induced autophagy initiation

AMPK pathway is the main autophagy-regulated signaling pathway and tightly linked to the status of mitochondria. We then tended to identify whether AMPK pathway was involved in CPX-induced autophagy in CRC cells. As shown in Fig. S6A and B, CPX treatment resulted in activation of AMPK and inhibition of mTOR pathway, as evidenced by increased phosphorylation levels of AMPK both in CPX-treated cells and CPX-treated xenografts. To determine whether the activation of AMPK was involved in CPX-induced autophagy, we transfected AMPK siRNA or domain negative AMPK (DN-AMPK) to inhibit AMPK activation. As shown in Fig. S6C-F, inhibition of AMPK prominently inhibited elevation of LC3 lipidation, endogenous LC3 puncta accumulation, and cytoplasmic AVO formation in CPX-treated cells. Thus, these results suggest that CPX-induced autophagy is dependent on AMPK activation in CRC cells.

In the above study, we have found that CPX could downregulate DJ-1 in CRC cells. We wondered whether CPX-induced downregulation of DJ-1 was involved in CPX-induced autophagy. To ascertain this hypothesis, we transfected a Myc-DJ-1 to enforce the exogenous expression of DJ-1 in CRC cells. As shown in Fig. S7A and B, exogenous expression of DJ-1 inhibited activation of AMPK and elevation of LC3 lipidation both in CPX-treated cells and CPX-treated xenografts. This was further confirmed by decreased formation of endogenous LC3 puncta, reduced AVO formation, and diminished colocalization between LC3 and LAMP1 (Fig. S7C-E). In contrast, knockdown DJ-1 could induce elevation of LC3 lipidation (Fig. S7F). Therefore, these data demonstrate that CPX induces autophagy through downregulation of DJ-1 in CRC cells.

Next, we also elucidated the role of ROS accumulation in CPX-induced autophagy. As shown in Fig. S8A-C, combinatorial treatment of CPX with ROS scavenger (NAC) resulted in AMPK inhibition, reduced LC3-II turnover, decreased accumulation of endogenous LC3 puncta and AVO formation. Moreover, we examined the protein levels of LC3 in CRC cells treated with CPX or/and apocynin, rotenone and nordihydroguaiaretic acid (NDGA) used to block NADPH oxidase, mitochondrial and 5-LOX-driven ROS release, respectively. The results shown that only rotenone can reduce LC3-II turnover (Fig. S8D and S8E). Collectively, these findings reveal that CPX-induced autophagy is dependent on increased mitochondria ROS levels in CRC cells.

### Inhibition of autophagy augments the anti-CRC effects of CPX

**Figure 7:**
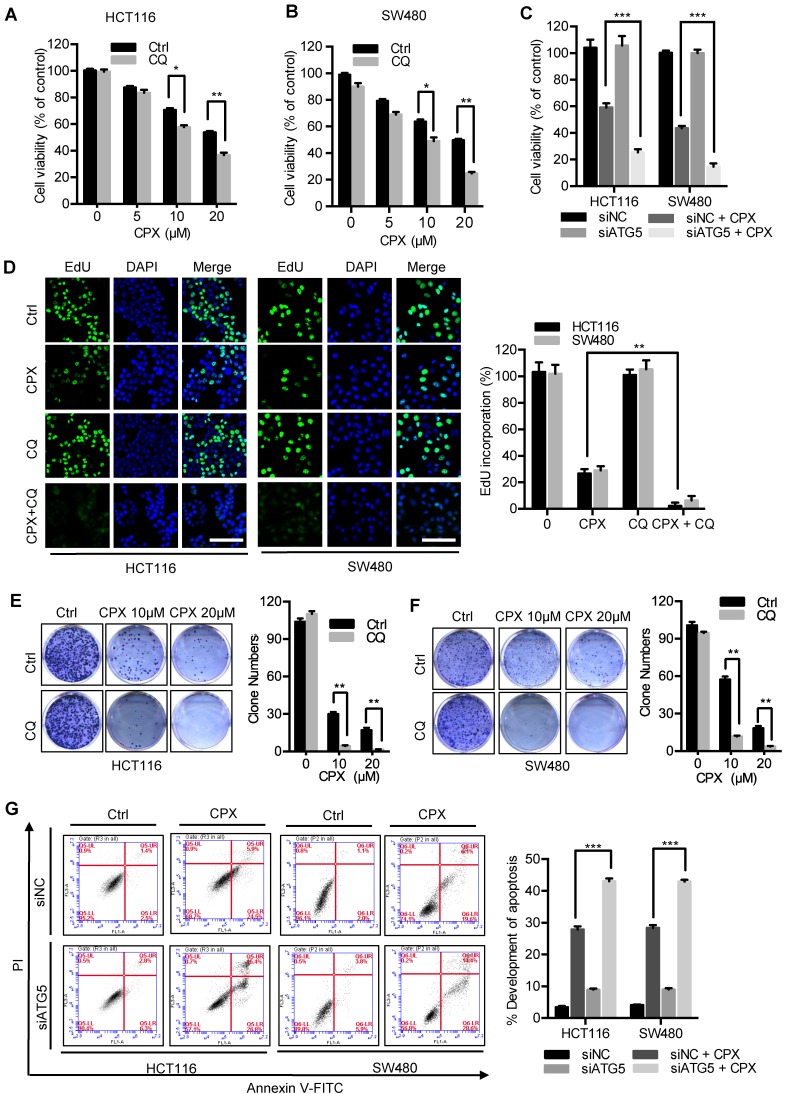
** Inhibition of autophagy augments the anti-CRC effects of CPX. A** and** B.** The cell viability of CRC cells treated with indicated concentrations of CPX in the presence or absence of 10 μM CQ for 24 hours was detected by CCK8 assays. **C.** The cell viability of CRC cells transfected with siNC or siATG5 for 24 hours, followed by treatment with or without 20 μM CPX for another 24 hours was detected by CCK8 assays. **D.** EdU assay of CRC cells treated with or without 10 μM CQ in the presence or absence of 20 μM CPX for 24 hours. The EdU incorporation was quantitated. Scale bar, 100 μm. **E** and** F.** HCT116 cells and SW480 cells were treated with indicated concentrations of CPX in the presence or absence of 10 μM CQ. Cell proliferation was detected by colony formation assay. **G.** CRC cells were transfected with siNC or siATG5 for 24 hours, followed by treatment with or without 20 μM CPX for another 24 hours. Cells were fixed, stained with Annexin V/PI, and then analyzed by flow cytometry. The development of apoptosis was quantitated. Data are means ± s.d. and are representative of 3 independent experiments. *, P < 0.05, **, P < 0.01, ***, P < 0.001.

To evaluate whether autophagy was involved in the anti-CRC effect of CPX, CRC cells were treated with CPX combined with autophagy inhibitor CQ. As shown in Fig. 7A and 7B, combinational use of CQ with CPX boosted CPX-induced growth inhibition. Consistently, similar results were obtained by inhibition of autophagy using siRNA-targeted *ATG5* knockdown (Fig. 7C). A similar decrease in cell proliferation was also observed in CPX-treated CRC cells in combination with CQ as evidenced by colony formation analysis and EdU labeling (Fig. 7D-7F). We also observed that *ATG5* knockdown could augment CPX-induced apoptotic cell death, as evidenced by Annexin V/PI staining measured with flow cytometry (Fig. 7G). In conclusion, these results suggest that inhibition of autophagy augments the anti-CRC effects of CPX.

### Transcriptional inhibition of DJ-1 by RUNX1

**Figure 8:**
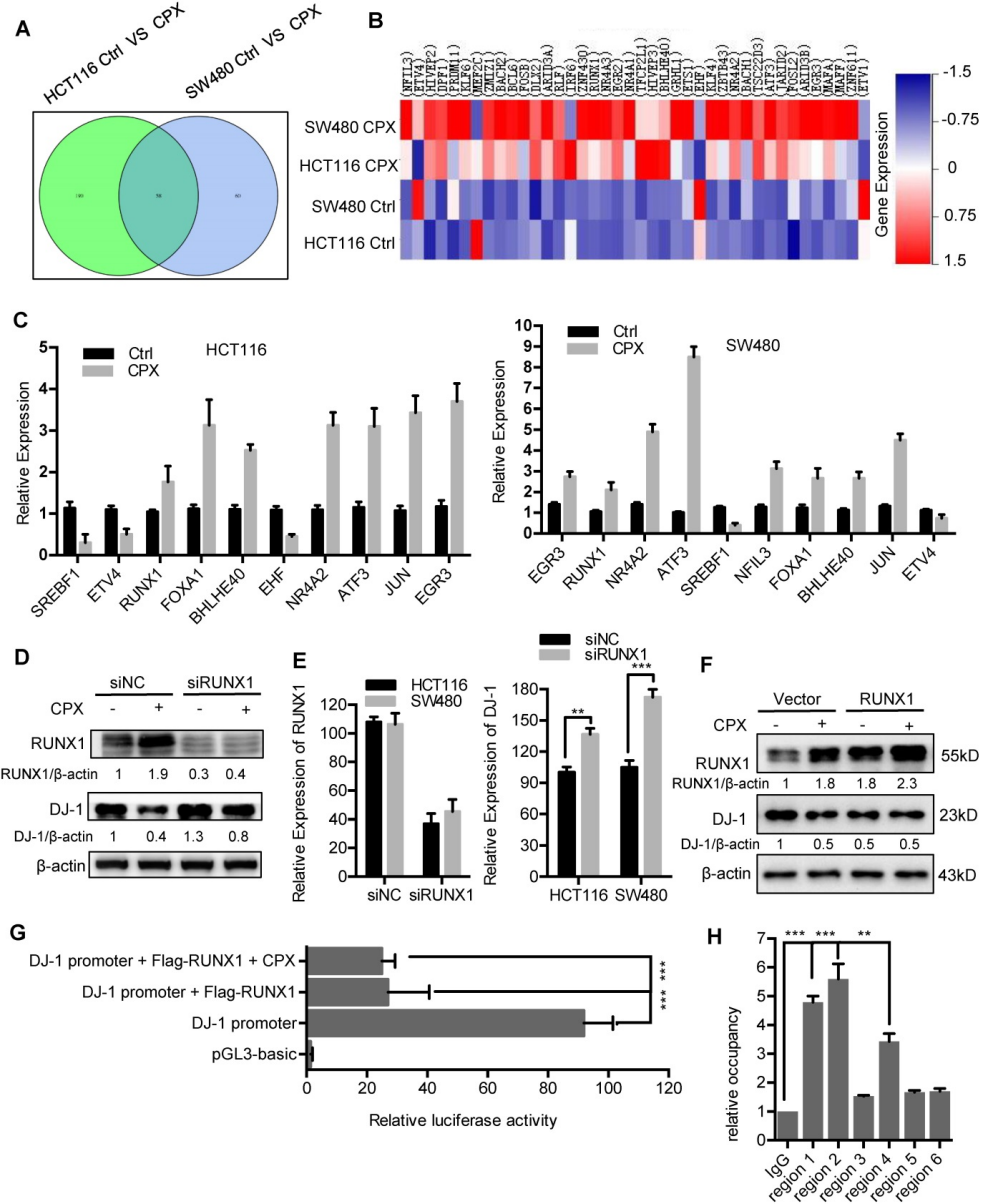
** Transcriptional inhibition of DJ-1 by RUNX1. A.** Venn diagram analysis of transcription factors was performed between HCT116 Ctrl VS CPX and SW480 Ctrl VS CPX. **B.** Heat map representation of normalized intensity values for transcription factors binding to DJ-1 promoter in HCT116 and SW480 cells treated with or without 20 μM CPX. **C.** qRT**-**PCR was conducted in HCT116 and SW480 cells to determine the mRNA levels of genes from (B). **D.** HCT116 cells were transfected with siRUNX1 for 24 hours, followed by treatment with or without 20 μM CPX for 24 hours. The protein levels of DJ-1 were examined by Immunoblotting. **E.** HCT116 cells were transfected with siNC or siRUNX1 for 24 hours, mRNA expression of DJ-1 and RUNX1 was examined by qRT-PCR. **F.** Immunoblot analysis of DJ-1 and RUNX1 expression in HCT116 cells transiently transfected with Vector or Flag-RUNX1 plasmid for 24 hours, followed by treatment with or without 20 μM CPX for another 24 hours. **G.** Luciferase-reporter studies on DJ-1 promoter in HCT116 cells transiently transfected with Vector or Flag-RUNX1 plasmid in the presence or absence of 20 μM CPX. **H.** Chromatin from HCT116 cells transfected with RUNX1 plasmid for 48 hours was used for ChIP analysis with RUNX1 antibody, followed by qRT-PCR to amplify different RUNX1-binding site regions of DJ-1 promoter. Enrichments were calculated relative to IgG. The qRT-PCR primer sequences were shown in Supplementary Table 4. Data are means ± s.d. and are representative of 3 independent experiments. *, P < 0.05, **, P < 0.01, ***, P < 0.001.

The mRNA expression of DJ-1 was significantly inhibited by CPX treatment (Fig. 2B). Herein, to explore whether DJ-1 was downregulated at a transcriptional level, we analyzed the expression of transcription factors under CPX treatment using transcriptome sequencing in HCT116 and SW480 cells. As shown in Fig. 8A and 8B, the expression of various transcription factors was regulated by CPX. Quantitative PCR further confirmed the mRNA levels of several transcription factors (Fig. 8C). To validate which of these transcription factors mediated the suppression of DJ-1, we interfered those using specific siRNAs, and found that silence of RUNX1 restored, at least partially, CPX-induced DJ-1 downregulation (Fig. 8D, E and S9A, B). Consistently, overexpression of RUNX1 inhibited the expression of DJ-1, which could not be further inhibited by CPX treatment (Fig. 8F), while overexpression of NFIL3, FOXA1, ETS1 showed no observed effect on DJ-1 expression (Fig. S9C). Promoter prediction revealed that nine RUNX1 binding sites exist in DJ-1 promoter region (Fig. S10). We then cloned DJ-1 promoter sequence into pGL3-luciferase reporter vector and conducted luciferase reporter analysis. As shown in Fig. 8G and S9D, compared to other transcription factors regulated by CPX, only expression of RUNX1 markedly suppressed the luciferase activity, while CPX treatment failed to show additional effect on DJ-1 promoter activity, indicating that RUNX1 bound to DJ-1 promoter and inhibited DJ-1 transcription. To investigate whether RUNX1 directly bound to DJ-1 promoter, we used chromatin immunoprecipitation (ChIP) assay. Six sets of PCR primers which contained nine predicted RUNX1-binding sites were constructed (Table S4). As shown in Fig. 8H, regions 1, 2 and 4, which contained RUNX1 binding sites 1, 2, 3 and 7 in DJ-1 promoter, were found to bind with RUNX1. Taken together, these results suggest that CPX-induced DJ-1 inhibition is largely due to the upregulation of RUNX1, which transcriptionally inhibits DJ-1 by directly binding to its promoter.

## Discussion

CPX is an off-patent antifungal agent, the effectiveness and safety of which have been well demonstrated 31-34. Recently, CPX has been found to have considerable potential to inhibit various types of cancer 7, 9, 35-37. It is of great interest to reveal activities and mechanisms of CPX in cancer treatment. In the present study, we demonstrate that CPX exhibits potent antitumor effect on CRC both *in vitro* and *in vivo*. CPX inhibits the growth of CRC via inhibition of cell proliferation and induction of apoptosis, which are involved in the downregulation of DJ-1. Loss of DJ-1 protein results in the mitochondrial dysfunction, which leads to the accumulation of ROS, thus inhibiting cell proliferation and inducing apoptosis. Interestingly, downregulation of DJ-1 simultaneously provokes autophagy, which plays a cytoprotective role under CPX treatment. Blockage of autophagy using CQ or ATG5 siRNA markedly enhances the antitumor effect of CPX. Our results provide supporting evidence for DJ-1 acting as a modulator of autophagy to benefit anti-cancer efficacy during anti-cancer agent treatment.

The therapeutic potential of CPX has attracted increasing interest. Numerous studies have suggested the antitumor activity of CPX. Previous pharmacology and toxicology study have confirmed that the LD50 value of CPX in mice, rats, and rabbits ranged from 1700 to 3290 mg/kg after oral administration 38, 39. In mammals, the CPX serum concentrations of 10 μM were achievable without toxicity when the compound is administered orally in rats and dogs 38, 39. Recently, several groups have demonstrated that CPX, given to nude mice at 20-25 mg/kg/day, potently inhibit tumor growth in myeloid leukemia, breast cancer and pancreatic cancer xenografts, but does not display obvious toxicity 33, 40. Additionally, after oral administration to humans, 96% of the administered CPX could be recovered from urine and the drug concentrations of CPX was pharmacologically achievable, although its half-life is short and requires dosing repeatedly 39. Notably, short half-life may represent a limitation for the use of CPX, and the use of CPX prodrugs or new drug delivery systems may rewire a way to improve pharmacodynamic profile. For example, Andrew M. Davidoff *et al.* delivered CPX via a subcutaneously implanted, continuous release pump to treat neuroblastomas 11. More importantly, John A. Taylor III *et al.* designed and synthesized the phosphoryl-oxymethyl ester of CPX, fosciclopirox (CPX-POM) which has higher oral bioavailability and is able to be metabolized rapidly and completely to the active CPX when intravenously injected 41. The safety, dose tolerance, pharmacokinetics and pharmacodynamics of intravenous CPX-POM are currently being characterized in a United States multi-center first-in-human Phase 1 clinical trial in patients with advanced solid tumors (NCT03348514). In addition, CPX has obvious activity to regulate inflammation, thus, it may exert anti-cancer role by regulating tumor microenvironment. A recent report showed that CPX could stimulate immunogenic cell death in pancreatic cancer 42.These studies suggest that CPX can be benefit for treating solid tumors.

CPX treatment resulted in a remarkable decrease of DJ-1 expression, which played pivotal roles in CPX-induced CRC inhibition, suggesting that CPX exerted the anti-CRC activity, at least partially, by targeting DJ-1. In cancer cells, DJ-1 has been identified to be a negative regulator of the tumor suppressor PTEN in primary breast cancer and non-small cell lung carcinoma (NSCLC) 43. Moreover, DJ-1 is upregulated and correlated with patient prognosis in various types of cancer, including NSCLC 43, ovarian carcinoma 44, and CRC 23, indicating that DJ-1 acts as an oncogene and can be a potential therapeutic target for cancer treatment 17. DJ-1 protein expression has been revealed to be downregulated by CPX treatment in HeLa cells through 2D gel electrophoresis 45. However, the underlying mechanism remains unclear. In this study, we found that CPX decreased the mRNA levels of DJ-1. Further investigations revealed that CPX treatment resulted in upregulation of RUNX1 to bind to the promoter region of DJ-1 and subsequently inhibit its transcription, implying a transcriptional regulation of DJ-1 in CRC cells.

DJ-1 is widely suspected as a cytoprotective protein in cancer cells. DJ-1 acts as an endogenous antioxidant to eliminate ROS by provoking antioxidant system, including nuclear factor erythroid 2-related factor (NRF2) 46 and glutathione 47. DJ-1 can also prevent oxidative stress-induced apoptosis 48. Mechanistic study revealed that DJ-1 blocks ASK1 activation by sequestering the death-associated protein Daxx in the nucleus 49, or by preventing the dissociation of ASK1 from Trx1 during oxidative stress 50, which negatively regulates apoptosis by inhibiting ASK1 51. Intriguingly, a recent study has revealed that in dihydroartemisinin-resistant HeLa cells, DJ-1 is overexpressed and translocated to the mitochondria 52. Consistent with these observations, our results showed that CPX-induced ROS accumulation was largely attributed to the downregulation of DJ-1, as restoring DJ-1 expression dramatically decreased ROS level. Moreover, CPX treatment resulted in mitochondrial dysfunction, which was responsible for ROS generation. This may be caused, at least partially, by the downregulation of DJ-1, supported by the observations that DJ-1 acts in parallel to the PINK1/parkin pathway to keep mitochondrial function, and its deficiency results in mitochondrial dysfunction and ROS accumulation 53, 54. Therefore, the elevated generation via mitochondrial dysfunction, together with the impaired removal due to DJ-1 loss, resulted in the robust accumulation of ROS.

ROS played a central role in CPX-mediated CRC growth inhibition, as the scavenging of ROS using NAC significantly abolished the anti-cancer effect of CPX (Figure 3). It has been widely considered that therapeutic stimulation of ROS can be a potent strategy to preferentially eliminate cancer cells 55, 56. More intriguingly, in addition to inducing apoptosis, CPX-induced ROS simultaneously provoke robust autophagic flux, which generally acts as a stress-eliminating process. The role of autophagy in response to therapeutic treatment is multifaceted 24. The induction of autophagy by therapeutic agents can be pro-death or pro-survival, contributing to the anti-cancer efficacy or drug resistance 57-59. In most cases, autophagy plays a pro-survival role during anti-cancer treatments in various types of cancer 60. This cytoprotective autophagy impedes the effectiveness of cancer therapies. Growing evidence has shown that autophagy inhibition could enhance the efficacy of anti-cancer agents, suggesting autophagy as a promising target for cancer therapy 61-64. Indeed, in this study, the combination of CPX with ATG5 siRNA or CQ treatment, which blocked autophagy initiation or autophagosome degradation, respectively, resulted in remarkable decrease of cell proliferation and increase of apoptosis compared with CPX treatment alone. In summary, we conclude that DJ-1 acts as the first protection for CRC, its loss results in ROS accumulation and apoptosis. In this condition, cytoprotective autophagy is provoked by DJ-1 loss to serve as the second protection.

In this study, we reveal that CPX induces DJ-1 downregulation, leading to ROS accumulation, which mediates AMPK phosphorylation and autophagy induction. The cytoprotective autophagy perhaps arose compensatively in response to DJ-1 loss, thus implying a strategy of modulating autophagy in combination with therapeutic treatment. Our study thus identifies DJ-1 as a target of CPX in CRC, and provides a further insight into understanding the action and mechanism of cytoprotective autophagy during cancer therapy.

## Supplementary Material

Supplementary figures and tables.
