# Supplementary material for: CPX Targeting DJ-1 Triggers ROS-induced Cell Death and Protective Autophagy in Colorectal Cancer
Source: Theranostics. 2019 Jul 28;9(19):5577–94. doi: 10.7150/thno.34663 (PMC6735393; doi:10.7150/thno.34663)
Supplement: Supplementary file 1 — Supplementary figures and tables. [file thnov09p5577s1.pdf]

Supplementary figure legends

Figure S1. CPX inhibits cell growth via promoting apoptosis of CRC cells.

A. Table shows the median inhibitory concentration (IC50) of CPX in HCT116, DLD-1, RKO, HT29, SW480, HIEC and NCM460 cells. **B.** Quantification of colony numbers of DLD-1, RKO, HT29 and SW620 cells subjected to vehicle or 20 μM CPX for 24 hours. **C.** Morphology of DLD-1, RKO, HT29 and SW620 cells following treatment with vehicle or 20 μM CPX for 24 hours. Scale bar, 100 μm. **D.** HIEC cells treated with 20 μM CPX for 24 hours were fixed, stained with Annexin V/PI, and then analyzed by flow cytometry.

Figure S2. DJ-1 downregulation plays an important role in CPX-induced mitochondrial dysfunction. A-D CRC cells were transfected with siNC or siDJ-1 for 24 hours. **A.** Expression of DJ-1 mRNA was examined by qRT-PCR. **B.** ROS level was analyzed by DCFH-DA staining via flow cytometry. **C.** The number of mitochondrial DNA copies was determined by qRT-PCR. **D.** The mRNA expression of genes involved in oxidative phosphorylation was examined by qRT-PCR. **E.** CRC cells were transfected with empty Vector or Myc-DJ-1 plasmid for 24 hours, followed by treatment with or without 20 μM CPX for 24 hours. The mRNA expression of genes involved in oxidative phosphorylation was examined by qRT-PCR. **F.** The ECAR test was performed under XF Base Medium containing 2 mM glutamine (pH was adjusted to 7.40 ± 0.05 at 37°C) after injection of 10 mM glucose, 1.0 μM oligomycin (an inhibitor of mitochondrial membrane adenosine triphosphate synthase) and 50 mM 2-deoxyglucose (2-DG, a non-metabolizable glucose analog that inhibits

glycolysis) in HCT116 cells treated with or without 20 μM CPX for 24 hours (n = 3 per group). The OCR was analyzed during the glycolysis stress test in HCT116 cells treated with or without 20 μM CPX for 24 hours (n = 3 per group).

Figure S3. CPX induces autophagy in several types of cancer.

A. Immunoblot analysis of LC3 and p62 expression in DLD-1, HT29, and RKO cells treated with indicated concentrations of CPX for 24 hours. **B.** Time course analysis of LC3 expression in HCT116 and SW480 cells treated with CPX (20 μM) for 3, 6, 12, 24, 48 hours by immunoblotting. **C.** Immunoblot analysis of LC3 expression in MDA-MB-231, MCF-7, Hela, and A549 cells treated with CPX (20 μM) for 24 hours.

Figure S4. CPX induces autophagy initiation in CRC cells.

A. Immunoblot analysis of LC3 and Beclin 1 expression in CRC cells transfected with siNC or siBeclin 1 for 24 hours, followed by treatment with or without 20 μM CPX for another 24 hours. **B.** Immunoblot analysis of LC3 and ATG5 expression in CRC cells transfected with siNC or siATG5 for 24 hours, followed by treatment with or without 20 μM CPX for another 24 hours. **C.** CRC cells were treated as in (A). The formation of endogenous LC3 puncta by immunofluorescence analysis was shown and quantitated. Scale bar, 20 μm. **D.** CRC cells were treated as in (B). The formation of endogenous LC3 puncta by immunofluorescence analysis was shown and quantitated. Scale bar, 20 μm. **E.** CRC cells were treated with vehicle, CPX (20 μM), wortmannin (10 μM), or in combination for 24 hours. Immunoblot analysis was used to detect the expression of LC3. Data are means ± s.d. *, $P < 0.05$, **, $P < 0.01$, ***, $P < 0.001$.

Figure S5. CPX promotes autophagy flux in CRC cells

A. The accumulation of LC3 puncta was examined by immunofluorescent analysis of cells treated with Baf A1 (100 nM) in the presence or absence of CPX (20 μM) for 24 hours. Scale bar, 20 μm. **B.** The accumulation of LC3 puncta was examined by immunofluorescent analysis of cells treated with E64D and PepA in the presence or absence of CPX (20 μM) for 24 hours. Scale bar, 20 μm.

Figure S6. CPX-induced autophagy is dependent on AMPK activation in CRC cells.

A. CRC cells were treated with indicated concentrations of CPX. The total and phosphorylation levels of AMPK, mTOR, p70S6K, and 4EBP1 were examined by immunoblotting. **B.** The phosphorylation and total levels of AMPK in vehicle- or CPX-treated mice bearing HCT116 xenografts were examined by Immunoblotting. **C.** Immunoblot analysis of LC3, total AMPK and p-AMPK expression in CRC cells transfected with siNC or siAMPK for 24 hours, followed by treatment with or without 20 μM CPX for another 24 hours. **D.** CRC cells were treated as in (C). The formation of endogenous LC3 puncta was shown and quantitated by immunofluorescence analysis. Scale bar, 20 μm. **E.** CRC cells were transiently transfected with Vector or DN-AMPK plasmid for 24 hours, followed by treatment with or without 20 μM CPX for another 24 hours. Autophagy was measured by acridine orange staining. Scale bar, 10 μm. **F.** CRC cells were treated as in (E). Immunoblot analysis was used to detect the expression of LC3 and p-AMPK. Data are means ± s.d. *, $P < 0.05$, **, $P < 0.01$, ***, $P < 0.001$.

Figure S7. CPX induces autophagy through down-regulating DJ-1 in CRC cells.

A. CRC cells were transiently transfected with Vector or Myc-DJ-1 plasmid for 24 hours, followed by treatment with or without 20 μM CPX for another 24 hours. The expression of DJ-1, LC3, total AMPK and p-AMPK was examined by immunoblotting. **B.** The levels of DJ-1, p-AMPK, total AMPK and LC3 in CPX-treated mice bearing HCT116 xenografts or HCT116-DJ-1 xenografts were examined by Immunoblot analysis. **C.** CRC cells were treated as in (A). The formation of endogenous LC3 puncta was shown and quantitated by immunofluorescence analysis. Scale bar, 20 μm. **D.** CRC cells were treated as in (A). Autophagy was measured by acridine orange staining. Scale bar, 10 μm. **E.** Immunofluorescence analysis of the co-localization of endogenous LC3 and LAMP1 in CRC cells treated as in (A). Scale bar, 20 μm. **F.** Immunoblot analysis of LC3 and DJ-1 expression in CRC cells transfected with siNC or siDJ-1 for 24 hours, followed by treatment with or without 20 μM CPX for another 24 hours. Data are means ± s.d. *, $P < 0.05$, **, $P < 0.01$, ***, $P < 0.001$.

Figure S8. CPX-induced autophagy is dependent on increased ROS in CRC cells.

A. CRC cells were treated with CPX (20 μM) in the presence or absence of NAC (5 mM) for 24 hours. The expression of LC3, p-AMPK, total AMPK and DJ-1 were examined by immunoblotting. **B.** CRC cells were treated as in (A). The formation of endogenous LC3 puncta was shown and quantitated by immunofluorescence analysis. Scale bar, 20 μm. **C.** CRC cells were treated with CPX (20 μM) with or without NAC, Rotenone, APO, and NDGA, respectively. Autophagy was measured by acridine

orange staining. Scale bar, 10 μm. **D.** CRC cells were treated with CPX (20 μM) with or without Rotenone for 24 hours. The expression of LC3 was examined by immunoblotting. **E.** CRC cells were treated with APO and NDGA in the presence or absence of CPX (20 μM) for 24 hours. The expression of LC3 was examined by immunoblotting. Data are means ± s.d. *, P < 0.05, **, P < 0.01, ***, P < 0.001.

Figure S9. Regulation of DJ-1 expression by CPX-induced transcription factors.

A. HCT116 cells were transfected with indicated siRNA for 24 hours, followed by treatment with or without 20 μM CPX for 24 hours. The mRNA expression of indicated genes was examined by qRT-PCR. Data are means ± s.d. and are representative of 3 independent experiments. *, P < 0.05, **, P < 0.01, ***, P < 0.001. Statistical significance compared with respective control groups. **B.** HCT116 cells were transfected with indicated siRNA for 24 hours, followed by treatment with or without 20 μM CPX for 24 hours. The protein levels of DJ-1 and indicated transcription factors were examined by Immunoblotting. **C.** CRC cells were transfected with empty Vector or NFIL3, FOXA1 and ETS1 plasmid for 24 hours, followed by treatment with or without 20 μM CPX for another 24 hours. The protein levels of DJ-1 were analyzed by immunoblotting. **D.** Luciferase-reporter studies on DJ-1 promoter in HCT116 cells transfected with Vector or NFIL3, FOXA1 and ETS1 plasmid for 24 hours.

Figure S10. RUNX1-binding consensus sequence motif and DJ-1 promoter sequence

A. RUNX1 binding models were analyzed using JASPAR database.

B. DJ-1 promoter sequence (GenBank Accession number AB045294) with RUNX1 binding sites was shown. The +1 indicated the transcription start site and yellow areas indicated the translation start site analyzed by UCSC Genome Browser.

Figure S1. CPX inhibits cell growth via promoting apoptosis of CRC cells

A

IC50 values (μM) of CPX in Colorectal cancer cells						
RKO	SW480	HCT116	DLD-1	HT29	HIEC	NCM460
15	16	18	13	3	47	52

B

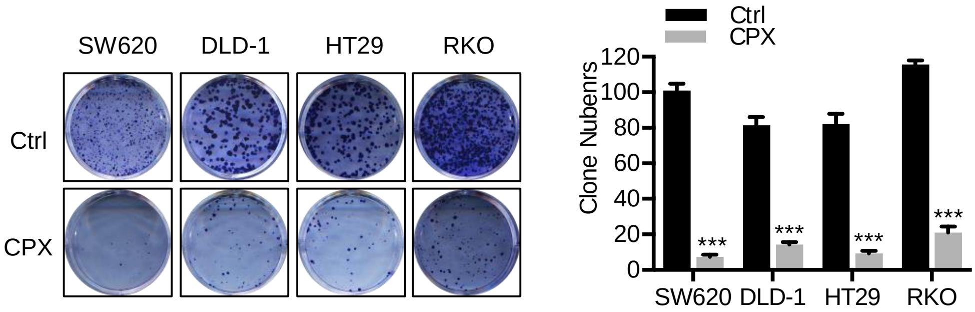

C

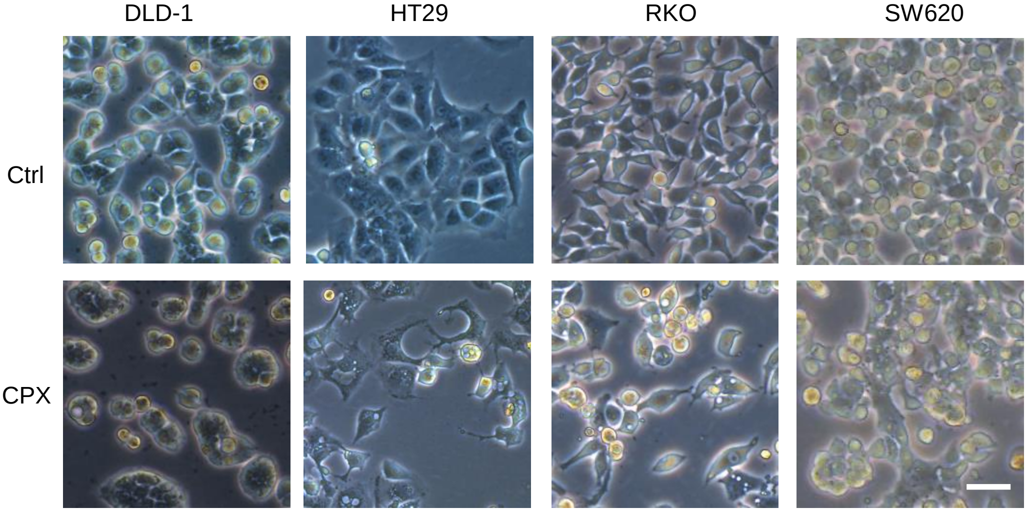

D

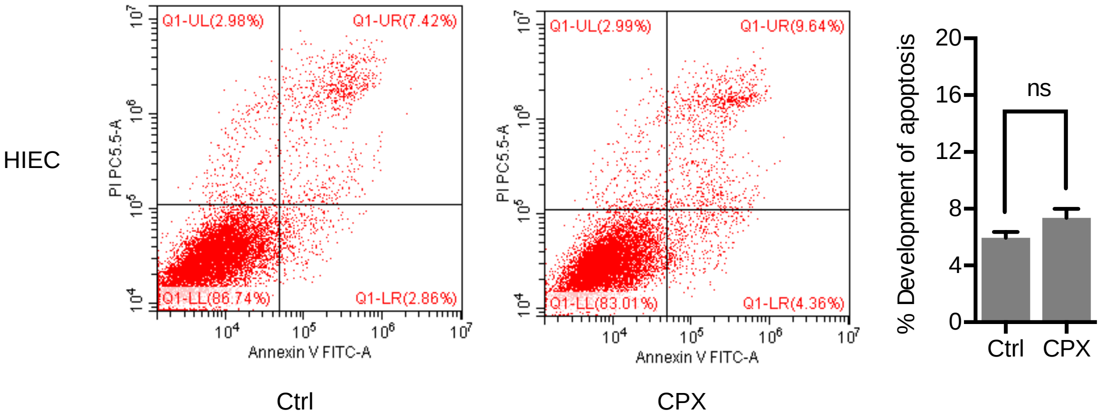

Figure S2. DJ-1 downregulation plays an important role in CPX-induced mitochondrial dysfunction

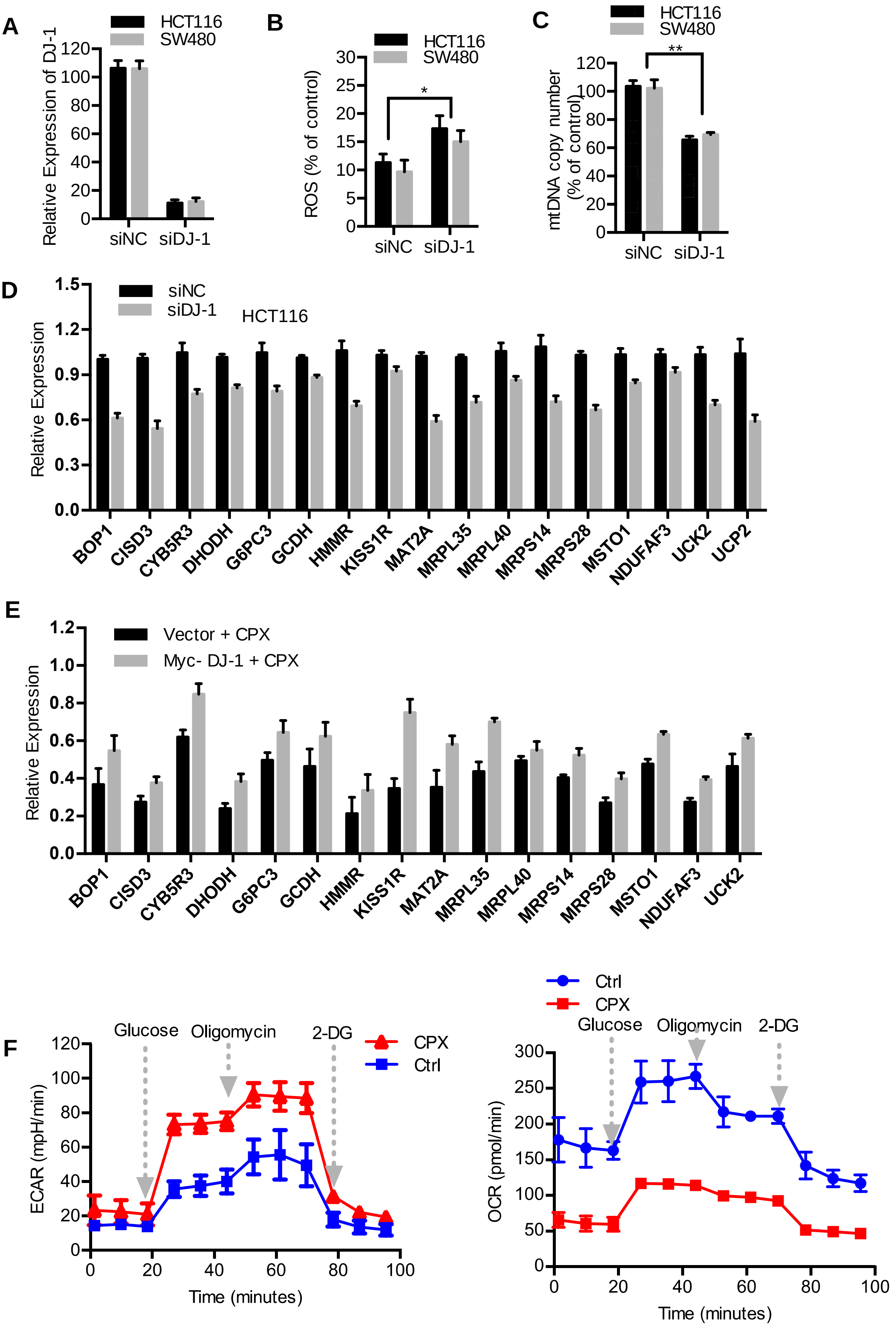

Figure S3. CPX induces autophagy in several types of cancer.

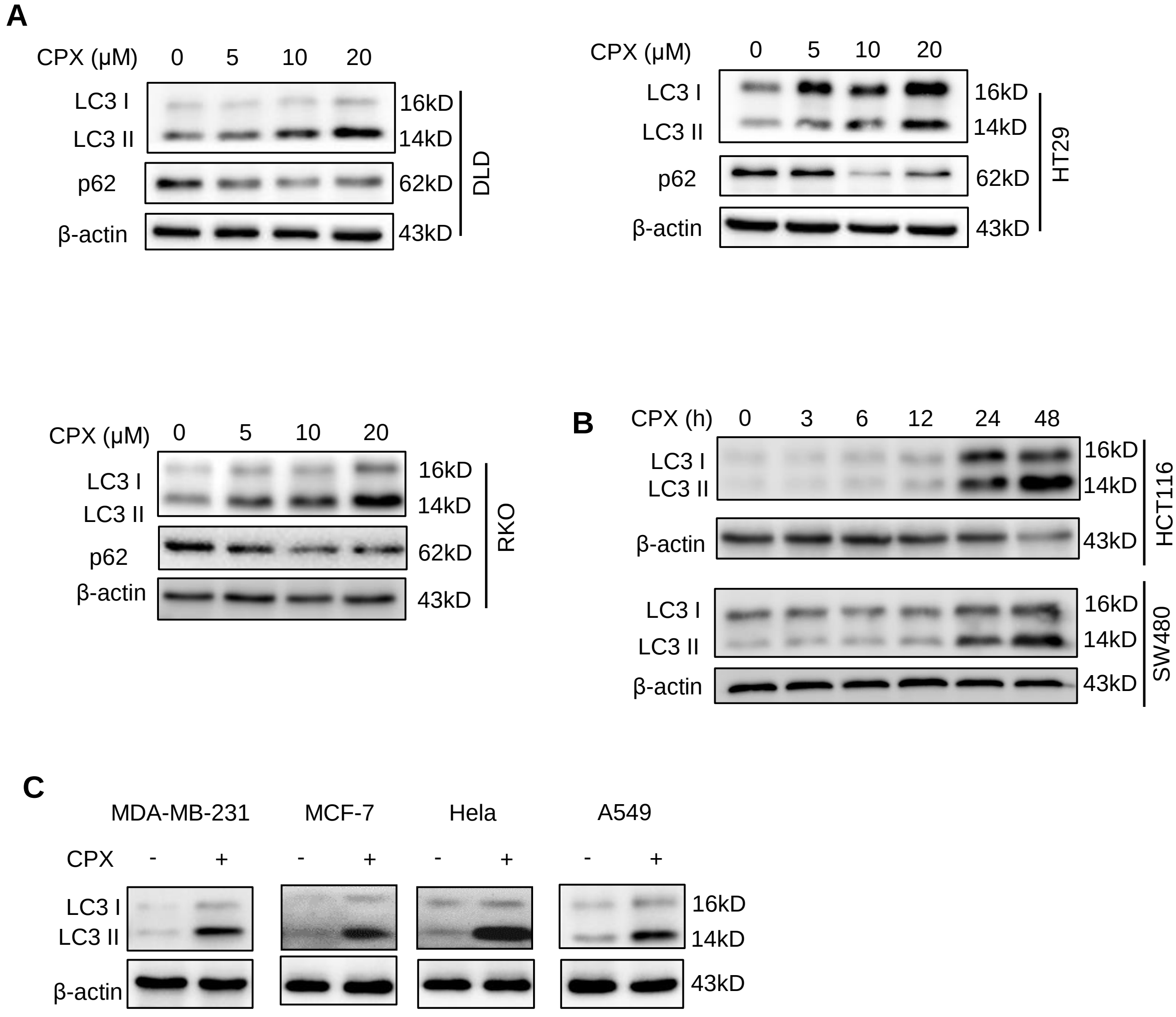

Figure S4. CPX induces autophagy initiation in CRC cells.

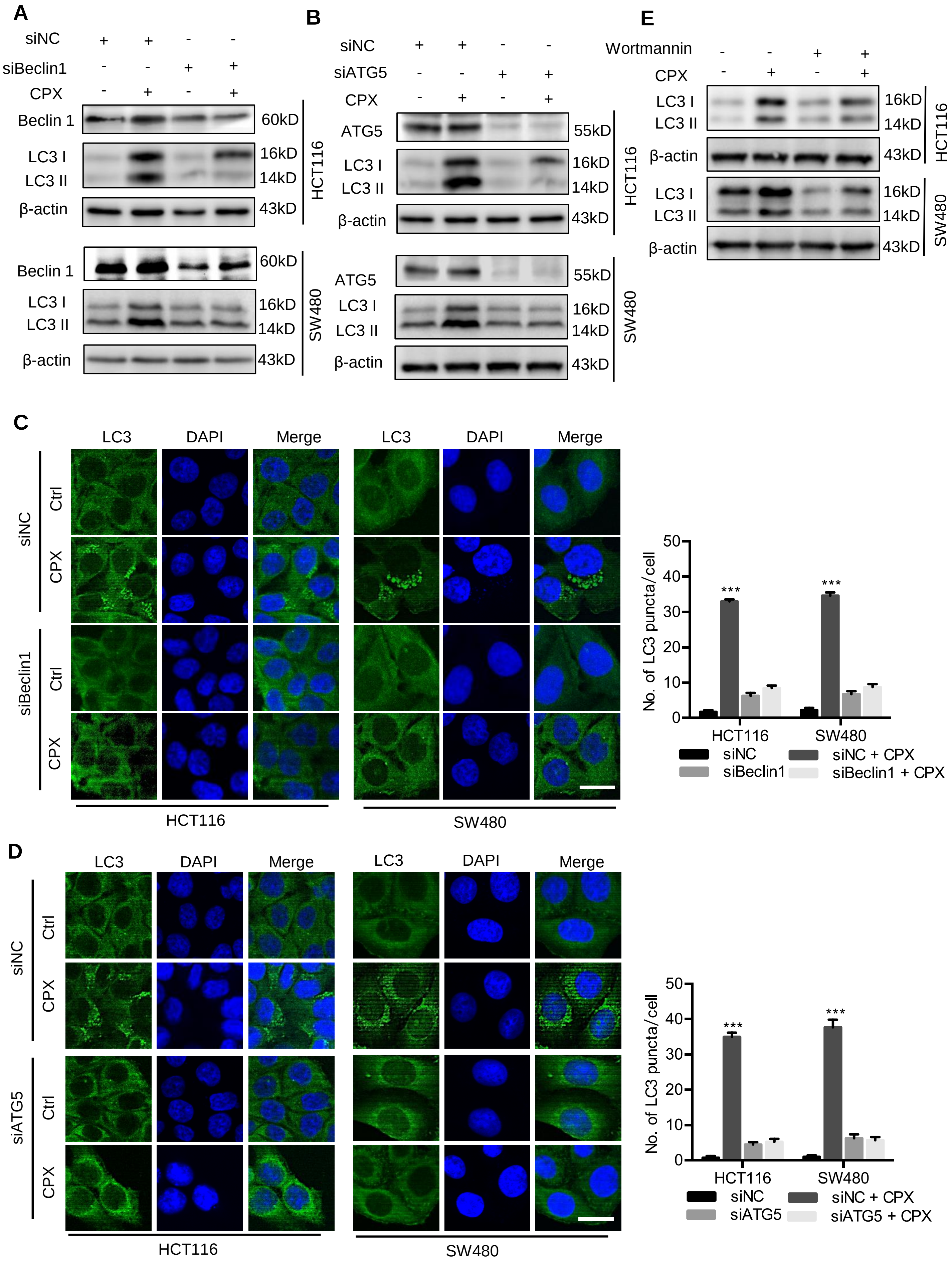

Figure S5. CPX promotes autophagy flux in CRC cells

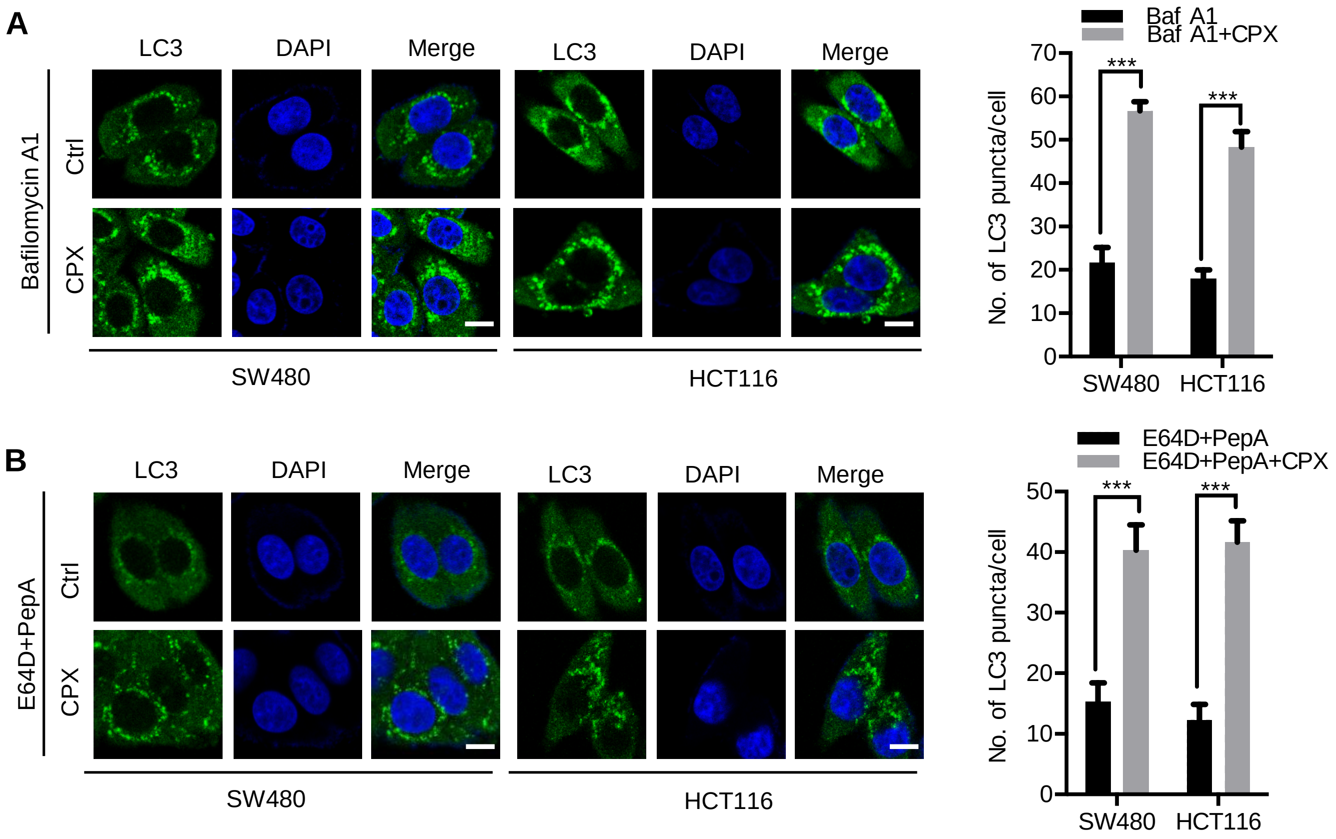

Figure S6. CPX-induced autophagy is dependent on AMPK activation in colorectal cancer cells

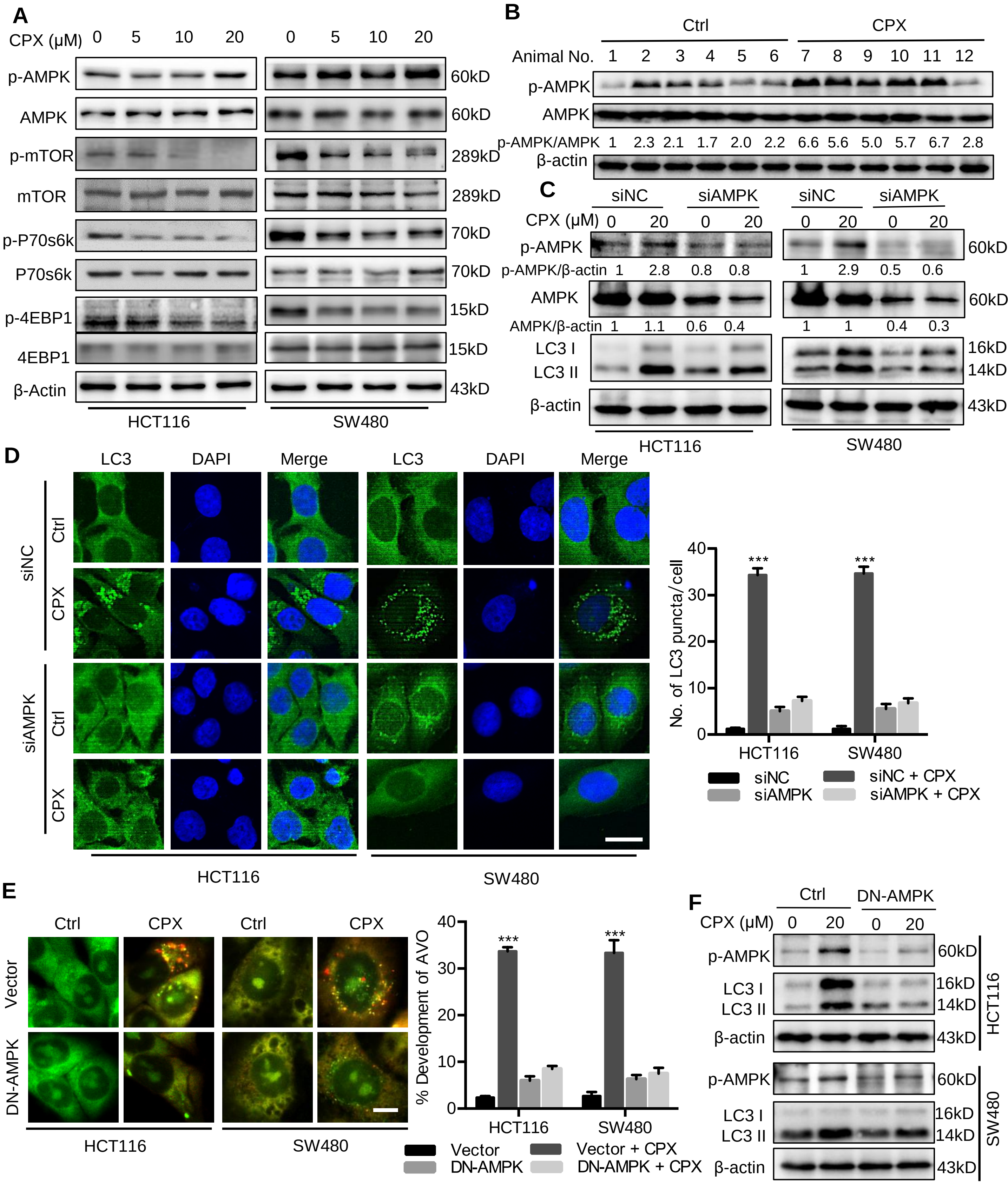

Figure S7. CPX induces autophagy through down-regulating DJ-1 in HCT116 and SW480 cells

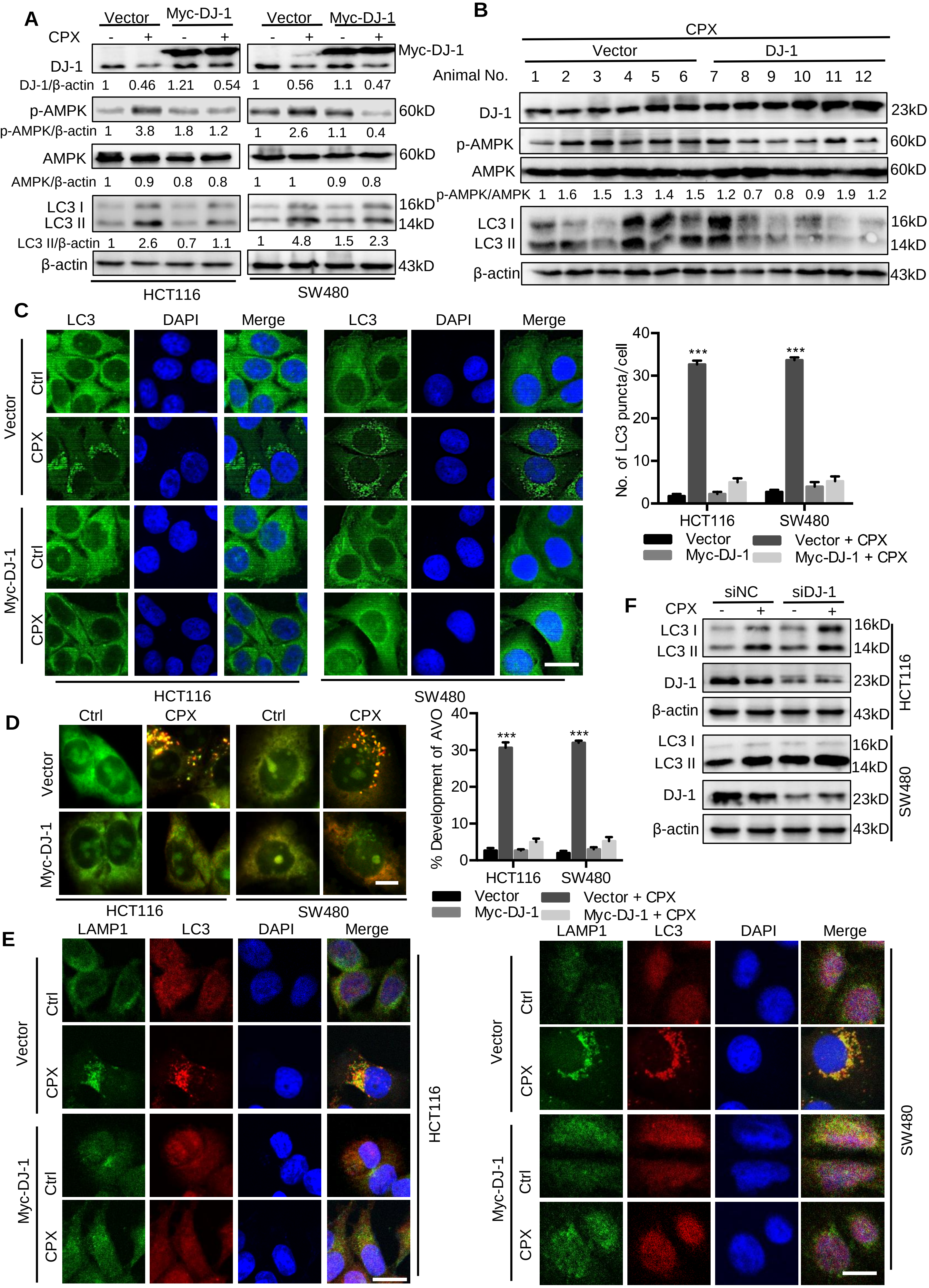

Figure S8. CPX-induced autophagy is dependent on increased ROS in colorectal cancer cells

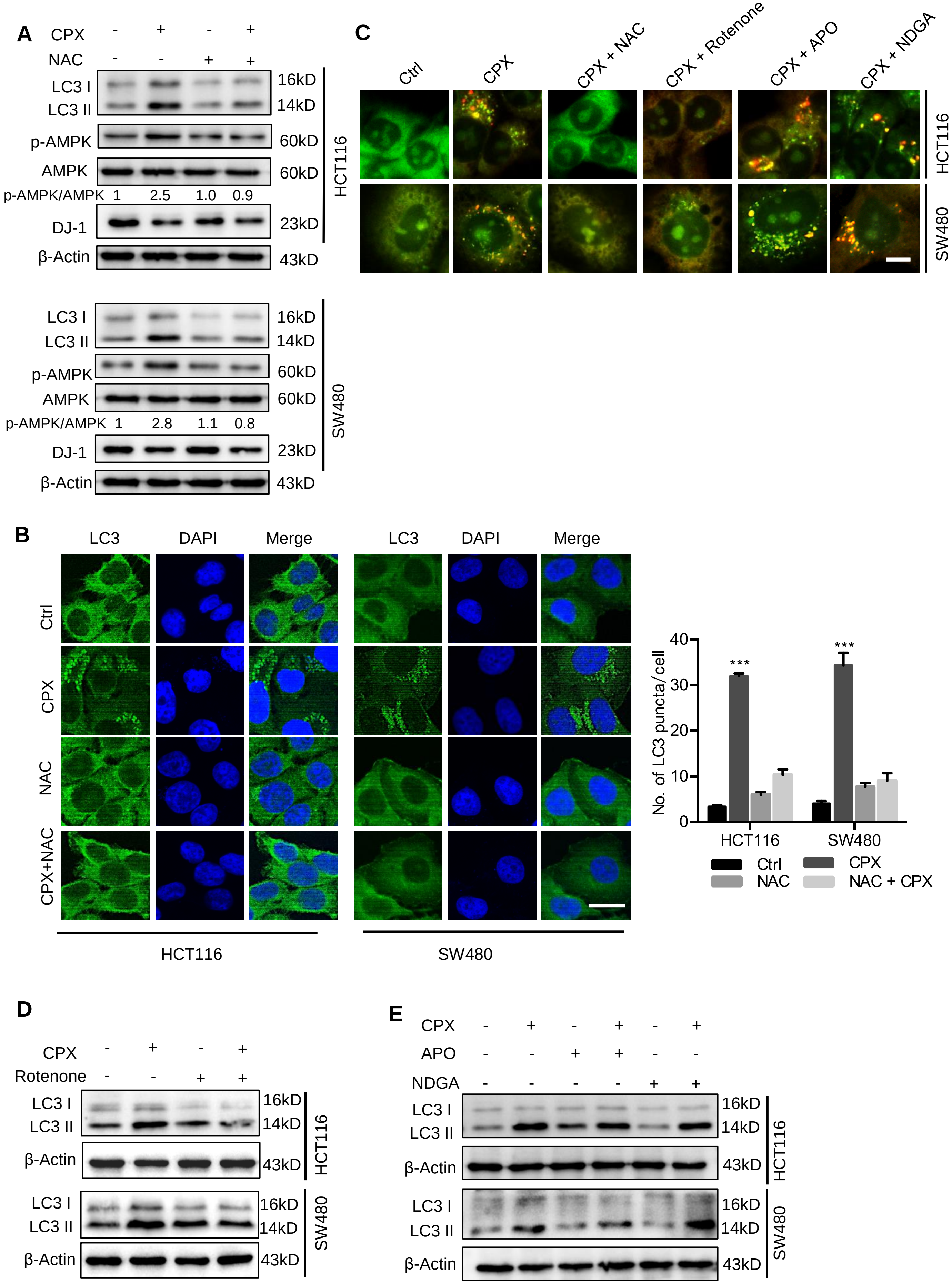

Figure S9. Regulation of DJ-1 expression by CPX-induced transcription factors

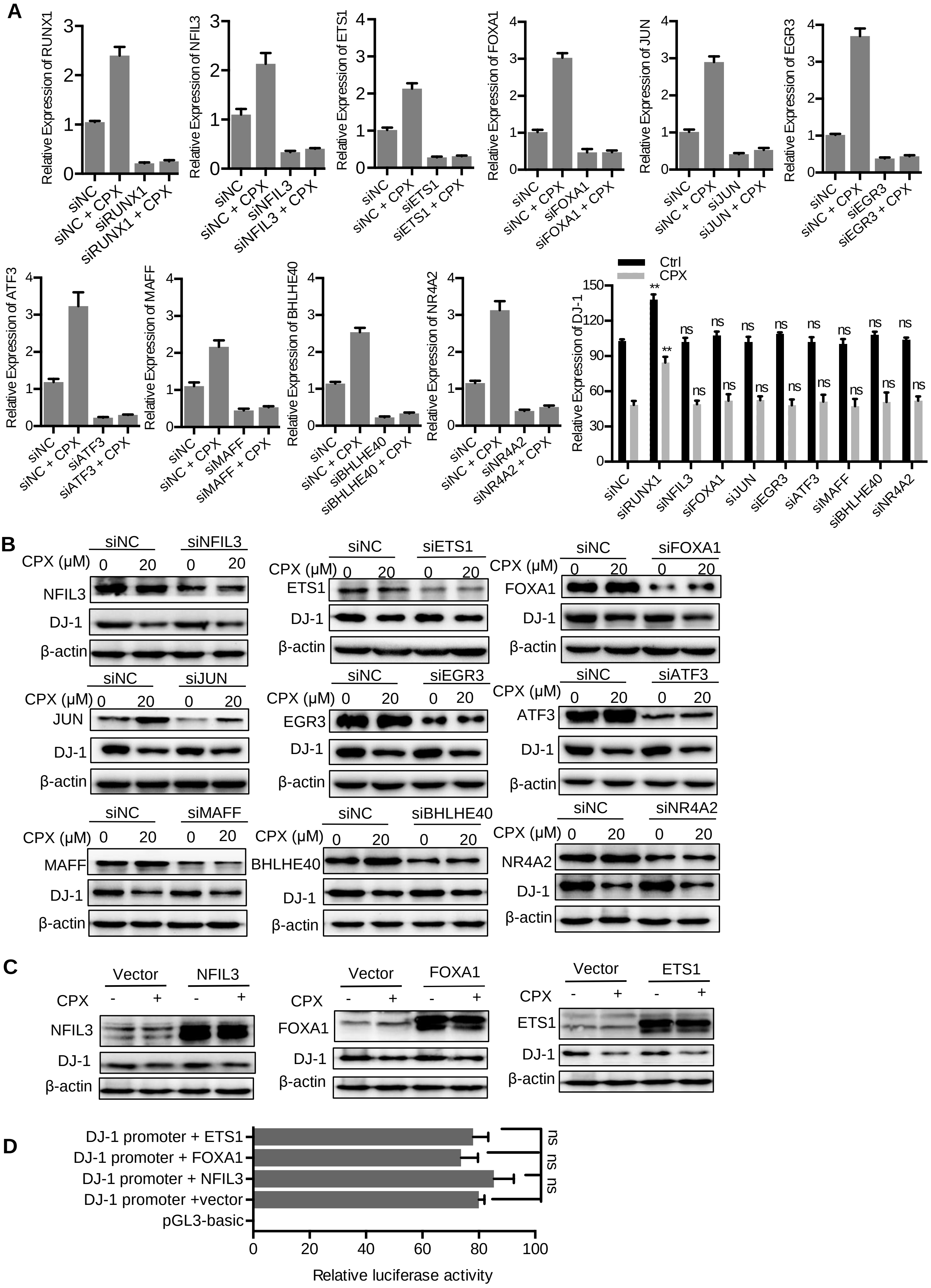

Figure S10. RUNX1-binding consensus sequence motif and DJ-1 promoter sequence

A

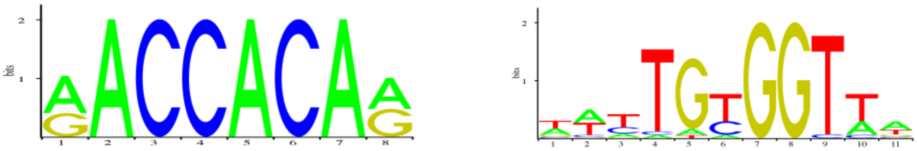

B

```
-1015 ggatccttct aagctcattc aagaattttg ggctttaact atttcctttg atttaacctg
 -955 gtaccaggtg ccaactttag ataataggga tatctaatta cttctaaatt cctcagataa
 -895 ggggcctgct tgatggtcac caggtgatct gtgctctcct taagagggaa taagacctag
 -835 cgttggcaga gttctgtagg gtgactatag ttaacagtaa tctgttgtat attttaaaat
                                         RUNX1 binding site 1
 -775 gttattattg aagagagtaa ctggaatgtt cccagtataa agacaaatgt ttaaggtgat
 -715 agagatctca tttaccctga tttaatcatt acacattata tgaaagtatc aaaataccac
                                                     RUNX1 binding site 2
 -655 atgtacccag aaaacacata cgtctcttac atatcaataa atacaacttg agattatgat
 -595 gtaaatacat ctgaccaact tggtacttat tagacttatg tgcgcagcac tgctctagtc
 -535 ctgtgggtgc agcagcatca ggatcgttaa agaaaacaaa caatgctgag aaaaaaactc
   RUNX1 binding site 3
 -475 acacccctga gacatccggg tgtgaataaa tgcggcagag tcgcccgaga tcgggagacc
 -415 aggcgtgggg gagaggtccg ggaggcctgg accagagtcc taacagacca gaggcgaaac
                          RUNX1 binding site 4   RUNX1 binding site 5
 -355 gggaaggcgc gccagaaaag gaacaacgca aagggagcag gcgtgcacgg agcgcgaact
 -295 aaggaacccc tctgacaacc ccagtccctc ggcagttcca gagaccggct cctcacggag
                 RUNX1 binding site 6
 -235 ggtggcggta gagactgtta agccccgcgg gcgccggggc aggccggact gtgccattcg
 -175 tgggggtac catgtgggac cgagccgcct cacccagggc tgtccagcta gaaactcccc
 -115 ggtgccaccc ccgcctcagt ccgaggtaga ctcggccgga cgtgacgcag cgtgaggcca
                                                               +1
  -55 aggcggcgtg agtctgcgca gtgtggggct gagggaggcc ggacggcgcg cgtgcgtgct
   +6 ggcgtgcgtt cactttcagc ctggtgtggg gtgagtggta cccaacgggc cggggcgccg
  +66 cgtccgcagg aagaggcgcg gggtgcaggt cagcgccagc gggggcgcgg cgcatgtgtg
 +126 ggccgtggcg ctgggcggcg tgggggtgct ggacggtgtc cctgtgctgg acggtgtccc
 +186 gctggctcag aaccggcgcg gggcctgggt cggggccgcc ctcgcttccg gcctcccagt
 +246 cgggccctgt cgctggcgtt ggatttgact gaccgccagc gtggtggcaa cgctgaagcg
 +306 tccagaatct tctgcctaac ctctcgccgg catggaactg gctagccgtt ttattaaact
 +366 ctgttttgcg tggacggtaa accctccaga taatctgtaa ataggttaaa aaaaattcgg
 +426 aacctcgttg agctgctgtc gttggcagtg agaactccgc gcagagagac agatgtagtt
                RUNX1 binding site 7
 +486 gggttgactt cagtgagggg atttccatct ttctcagtca ttaaaaaaag tgttcagaca
 +546 tttaacactg ttgaccccca cacacaattt tttagtacag ttataactaa gaaaacaaaa
 +606 atcccctcca aaaaattaca agttaattgc gaaagaccac atttaaattt ttgcccatga
                                     RUNX1 binding site 8
 +666 aattcagttt agtcgtttct ctgaaacagt gcttcaaaaa agactgtttc cccgcattgt
 +726 gtgaaatgca ggagacccac gtacttgtat ttttaaaaaa cccatttgca acatactatt
 +786 aaagttggat ttaagagaac atggtagaag aaaatctaag caatactaca ccttttagca
 +846 ccctcattat gttttcatct cagagcaatt aaaactgcta tacaaatcaa cgttaagata
 +906 actaaactgc tgcttttttc gtattcagtt gtctatgaaa accgtttccc taggaagtac
 +966 ttactctgct tgaaaatgct cctaaacttt aaattttggg gtatctcagg gttgcaatga
                                      RUNX1 binding site 9
+1026 aagtttttttg aaatcttttt tttttttttt ttttaaggct tgtaaacata taacataaaa
+1086 atggcttcca aaagagctc
```

Supplementary Table 1. Gene features significantly differentially expressed in HCT116 cells treated with CPX.

#ID	Ctrl_Count	cpx_Count	Ctrl_FPKM	cpx_FPKM	log2FC	regulated
ENSG00000002746(HECW1)	115	16	0.847009403	0.164299447	-2.598034492	down
ENSG00000003989(SLC7A2)	416	149	2.806435331	1.043053061	-1.414385237	down
ENSG00000004700(RECQL)	784	1681	9.4695	22.487016	1.147435537	up
ENSG00000005001(PRSS22)	229	528	11.800673	27.687708	1.244607591	up
ENSG00000005187(ACSM3)	128	9	2.224177	0.2121664	-3.420348861	down
ENSG00000005238(FAM214B)	225	556	4.522685338	11.17470571	1.34387043	up
ENSG00000006432(MAP3K9)	437	1074	1.952520995	4.86827876	1.341460067	up
ENSG00000006459(KDM7A)	274	781	1.585933017	4.430908758	1.551063175	up
ENSG00000006468(ETV1)	467	181	4.991307778	1.80655983	-1.304183124	down
ENSG00000006534(ALDH3B1)	516	203	12.488618	4.69753158	-1.284186216	down
ENSG00000006555(TTC22)	61	14	1.611480431	0.302423803	-1.876634538	down
ENSG00000006652(IFRD1)	606	1675	15.18250438	42.96952097	1.512254889	up
ENSG00000007376(RPUSD1)	2644	969	92.3080497	33.510655	-1.395584647	down
ENSG00000007520(TSR3)	1459	461	91.3951	29.0401	-1.60626826	down
ENSG00000008083(JARID2)	368	882	4.6566953	10.85312233	1.304275275	up
ENSG00000008086(CDKL5)	35	116	0.130559783	0.437249973	1.697979617	up
ENSG00000008294(SPAG9)	1800	4795	10.03113094	30.64902568	1.462025126	up
ENSG00000008382(MPND)	474	141	24.33308625	6.8797789	-1.679563796	down
ENSG00000008405(CRY1)	411	967	7.682039	19.32839	1.278361784	up
ENSG00000010017(RANBP9)	1514	3298	36.075	76.4378	1.171661202	up
ENSG00000010379(SLC6A13)	8	43	0.289153881	1.667683067	2.119963003	up
ENSG00000010626(LRRC23)	910	7972	20.43364259	177.7991854	3.1768815	up
ENSG00000010818(HIVEP2)	116	1167	0.530070223	5.30242445	3.348304089	up
ENSG00000011332(DPF1)	168	420	4.741346471	11.68044142	1.356749576	up
ENSG00000011422(PLAUR)	658	2493	26.74659522	97.91091826	1.966958525	up
ENSG00000013364(MVP)	1311	577	26.70272944	11.51192779	-1.130234881	down
ENSG00000013588(GPRC5A)	3055	9346	24.53869479	80.4899	1.662213236	up
ENSG00000019186(CYP24A1)	1953	407	34.29199	7.320740152	-2.204831768	down
ENSG00000019995(ZRANB1)	433	927	3.54116	7.867149	1.142882585	up
ENSG00000020577(SAMD4A)	115	482	0.830361913	3.4854613	2.089707633	up
ENSG00000021355(SERPINB1)	953	2179	22.148622	56.5794836	1.240555395	up
ENSG00000023330(ALAS1)	1416	3030	36.963367	76.236303	1.145848651	up
ENSG00000023445(BIRC3)	44	187	0.407485882	1.721236999	2.066559666	up
ENSG00000023839(ABCC2)	4240	1564	32.20643	12.589476	-1.387246223	down
ENSG00000024526(DEPDC1)	2069	772	23.78792	7.980766	-1.369068086	down
ENSG00000025708(TYMP)	1647	715	98.04425394	44.207492	-1.150734514	down
ENSG00000026103(FAS)	217	532	3.971610247	9.975977316	1.332090095	up
ENSG00000026652(AGPAT4)	160	1045	1.900083883	18.10517068	2.735203822	up
ENSG00000027075(PRKCH)	352	760	7.0477392	14.7668102	1.153847562	up
ENSG00000031081(ARHGAP31)	49	158	0.2330713	0.752998033	1.681247333	up
ENSG00000033178(UBA6)	1259	2715	7.168272885	14.20150189	1.15681221	up
ENSG00000034677(RNF19A)	468	1160	6.647296439	13.5877391	1.354068747	up
ENSG00000037897(METTL1)	889	387	29.626944	13.355535	-1.144146121	down
ENSG00000038427(VCAN)	381	822	2.624558337	4.93924805	1.153270673	up
ENSG00000039068(CDH1)	1342	3095	13.28079899	31.12749312	1.253715601	up
ENSG00000040633(PHF23)	1240	498	30.89224518	12.86141977	-1.261406574	down
ENSG00000043143(JADE2)	962	321	7.965653769	2.943895531	-1.525264782	down
ENSG00000047346(FAM214A)	56	204	1.604369061	4.003565671	1.861694983	up
ENSG00000047365(ARAP2)	356	793	4.754798819	10.06719284	1.198778849	up
ENSG00000047634(SCML1)	232	515	4.950834705	11.30194373	1.190291909	up
ENSG00000048162(NOP16)	1539	528	76.59347541	26.78577565	-1.488471233	down
ENSG00000048342(CC2D2A)	315	129	7.759033406	2.545941042	-1.220168825	down
ENSG00000049130(KITLG)	15808	5805	133.7303	51.72966	-1.394877461	down
ENSG00000049283(EPN3)	717	310	13.90193081	6.207939954	-1.152547907	down
ENSG00000049323(LTBP1)	122	277	2.599951821	3.732527928	1.213536284	up
ENSG00000049759(NEDD4L)	884	1886	11.80284276	32.88468957	1.140594673	up
ENSG00000050555(LAMC3)	353	126	3.153549101	1.08303913	-1.416277502	down
ENSG00000051108(HERPUD1)	781	1843	20.63835504	47.12979089	1.28547074	up
ENSG00000052795(FNIP2)	148	408	0.968368547	2.78199359	1.494759332	up
ENSG00000053747(LAMA3)	94	2088	0.862258932	15.88861833	4.481242189	up
ENSG00000054523(KIF1B)	865	1893	4.179183264	9.300185014	1.177173715	up
ENSG00000054611(TBC1D22A)	744	1591	15.60744996	34.72438332	1.143450082	up
ENSG00000055609(KMT2C)	411	1104	4.59867232	12.7704449	1.468995069	up
ENSG00000055950(MRPL43)	1785	702	139.594063	53.952685	-1.292988839	down
ENSG00000057294(PKP2)	636	1494	7.866029894	18.555604	1.278183135	up
ENSG00000057468(MSH4)	8	80	0.122518	1.21081	2.974085284	up
ENSG00000057657(PRDM1)	8	93	0.07849552	0.953501	3.184453145	up
ENSG00000057704(TMCC3)	150	365	1.252324804	3.051660918	1.316255039	up
ENSG00000058063(ATP11B)	595	1297	5.147482709	11.033325	1.170277293	up
ENSG00000058085(LAMC2)	346	2840	2.872376	23.93076	3.076365838	up
ENSG00000059691(GATB)	567	244	11.41885723	5.449891	-1.157349754	down
ENSG00000059804(SLC2A3)	54	528	0.77758762	7.870033	3.271408405	up
ENSG00000063180(CA11)	602	221	27.17498016	9.931486	-1.38446157	down

ENSG00000063241(ISOC2)	1122	316	74.96664451	20.5371407	-1.769058518	down
ENSG00000063761(ADCK1)	183	70	6.29453588	3.226302859	-1.302395119	down
ENSG00000064651(SLC12A2)	1067	2573	7.865794151	19.60234999	1.317513298	up
ENSG00000065057(NTHL1)	618	127	35.109222	7.429055	-2.207991481	down
ENSG00000065809(FAM107B)	879	1923	13.47727692	29.09726253	1.176738715	up
ENSG00000067057(PFKP)	5149	12070	108.2837612	254.9973782	1.278558709	up
ENSG00000067066(SP100)	555	1294	9.154858816	17.92657681	1.266839503	up
ENSG00000067082(KLF6)	1086	2903	11.24235219	31.48517017	1.466032139	up
ENSG00000067836(ROGDI)	408	163	15.21668685	5.93542398	-1.259304347	down
ENSG00000067992(PDK3)	107	371	3.274415121	11.279438	1.815957559	up
ENSG00000068971(PPP2R5B)	215	1093	5.01833215	25.128474	2.380168218	up
ENSG00000069020(MAST4)	70	316	0.32846231	1.513756004	2.178480751	up
ENSG00000069275(NUCKS1)	8930	3747	64.46045624	28.27304302	-1.20233579	down
ENSG00000070190(DAPP1)	2	78	0.06789964	1.302170591	4.090585334	up
ENSG00000070495(JMJD6)	1242	3054	34.672215	89.733148	1.345961971	up
ENSG00000070669(ASNS)	4118	9943	110.3295732	275.4090816	1.321102411	up
ENSG00000070778(PTPN21)	131	298	1.08823985	2.747274	1.217566464	up
ENSG00000071073(MGAT4A)	111	328	1.55747303	2.35375473	1.588084453	up
ENSG00000072274(TFRC)	6236	18409	70.946409	201.082	1.61124202	up
ENSG00000072571(HMMR)	2144	781	26.79132831	10.01634517	-1.403707075	down
ENSG00000072682(P4HA2)	459	2898	8.786622779	60.7316609	2.700747651	up
ENSG00000072952(MRVI1)	241	56	1.862724574	0.631148285	-2.002207039	down
ENSG00000073670(ADAM11)	352	93	4.275242847	1.293112367	-1.839209191	down
ENSG00000073712(FERMT2)	752	1587	15.14648547	28.55341454	1.124454557	up
ENSG00000073756(PTGS2)	8	142	0.0970282	1.72733	3.780358933	up
ENSG00000074071(MRPS34)	3157	971	216.7043	66.23244	-1.648192894	down
ENSG00000074410(CA12)	172	374	1.767601422	3.574847411	1.157202067	up
ENSG00000074416(MGLL)	105	305	1.624403505	4.096082466	1.562187642	up
ENSG00000074660(SCARF1)	15	58	0.279262094	1.04829218	1.813511041	up
ENSG00000074966(TXK)	268	869	2.300254324	7.670601	1.736176127	up
ENSG00000075213(SEMA3A)	247	65	3.108380433	0.509144326	-1.831834796	down
ENSG00000075426(FOSL2)	934	3844	7.524714128	31.63364422	2.087656396	up
ENSG00000075945(KIFAP3)	236	507	3.810520024	8.3572223	1.143469759	up
ENSG00000076356(PLXNA2)	30	104	0.2142167	0.689424	1.748421393	up
ENSG00000076513(ANKRD13A)	552	1228	6.42461	14.56275259	1.199259139	up
ENSG00000076641(PAG1)	72	496	0.325014783	2.266279	2.785230577	up
ENSG00000077157(PPP1R12B)	159	388	0.526063117	1.355119578	1.321252332	up
ENSG00000077585(GPR137B)	193	500	6.33503279	16.75115663	1.409798985	up
ENSG00000078114(NEBL)	305	961	2.665380704	6.94939563	1.696216183	up
ENSG00000078177(N4BP2)	254	755	1.218111408	3.822047165	1.610549357	up
ENSG00000078269(SYNJ2)	542	1992	8.047039283	23.00248707	1.922137576	up
ENSG00000078804(TP53INP2)	195	646	3.176005496	11.31956804	1.762927343	up
ENSG00000079150(FKBP7)	123	19	4.323242271	1.08227099	-2.479266057	down
ENSG00000079215(SLC1A3)	839	263	10.47679734	3.523676562	-1.613287297	down
ENSG00000079385(CEACAM1)	2	92	0.049270181	1.329928309	4.321086959	up
ENSG00000080031(PTPRH)	129	770	2.031032107	11.4051729	2.600533179	up
ENSG00000080200(CRYBG3)	111	381	0.44481	1.5569	1.802473045	up
ENSG00000080503(SMARCA2)	61	216	0.863219212	3.059721585	1.825539042	up
ENSG00000080546(SESN1)	254	706	4.779328062	14.06914115	1.514112325	up
ENSG00000080854(IGSF9B)	401	876	1.180405	2.761491	1.171494355	up
ENSG00000081052(COL4A4)	95	398	0.599740153	2.607483357	2.083364207	up
ENSG00000081181(ARG2)	63	500	1.68838	13.6144	2.981413057	up
ENSG00000081189(MEF2C)	641	269	6.98549088	3.045796657	-1.194277632	down
ENSG00000081692(JMJD4)	419	158	10.298213	3.962997	-1.341590648	down
ENSG00000082014(SMARCD3)	779	333	26.49815231	11.96253082	-1.169375717	down
ENSG00000082438(COBLL1)	34	170	0.266010051	1.596524019	2.27707079	up
ENSG00000082516(GEMIN5)	1550	601	12.4145	4.89943	-1.312828261	down
ENSG00000083223(ZCCHC6)	395	886	4.240904166	8.9283156	1.20940977	up
ENSG00000083799(CYLD)	191	556	1.320740108	4.8921766	1.576946863	up
ENSG00000083807(SLC27A5)	229	85	33.04730002	12.13818706	-1.35111117	down
ENSG00000084731(KIF3C)	186	404	1.757480477	3.822933876	1.156633578	up
ENSG00000085117(CD82)	240	597	9.706626458	19.15002448	1.354060464	up
ENSG00000085433(WDR47)	316	889	3.76030007	10.84454212	1.533563435	up
ENSG00000085552(IGSF9)	466	141	7.022057	2.091315553	-1.655157836	down
ENSG00000086189(DIMT1)	1466	534	28.51330001	11.243161	-1.40228275	down
ENSG00000086504(MRPL28)	1186	495	67.16047453	28.84276963	-1.20599241	down
ENSG00000086730(LAT2)	149	528	4.040489769	13.90804114	1.854954043	up
ENSG00000087128(TMPRSS11E)	0	39	0	0.95779	3.894520325	up
ENSG00000087586(AURKA)	1589	480	36.885586	12.17704806	-1.671251038	down
ENSG00000088726(TMEM40)	98	351	3.137129771	11.8716953	1.859880416	up
ENSG00000088854(C20orf194)	137	373	1.264089057	3.713162729	1.475480884	up
ENSG00000089041(P2RX7)	50	7	0.447191822	0.068298795	-2.381042742	down
ENSG00000089916(GPATCH2L)	762	2215	6.40030707	13.22011929	1.585750267	up
ENSG00000090020(SLC9A1)	537	1372	8.416897	20.531561	1.398417969	up
ENSG00000090447(TFAP4)	509	184	23.1614329	9.1758207	-1.404332463	down
ENSG00000091409(ITGA6)	1473	5403	12.52836	47.05858768	1.922882395	up

ENSG00000091428(RAPGEF4)	4	43	0.0542253	0.513940579	2.778815949	up
ENSG00000092098(RNF31)	1640	620	31.86490536	14.36944864	-1.34941011	down
ENSG00000092421(SEMA6A)	134	394	1.488734538	4.063207994	1.585196657	up
ENSG00000094841(UPRT)	157	1046	3.715686847	24.77398488	2.763394946	up
ENSG00000094914(AAAS)	1386	611	44.17513167	20.70997127	-1.128109772	down
ENSG00000094975(SUCO)	635	1689	5.803503555	15.37289006	1.457119252	up
ENSG00000095397(WHRN)	275	702	7.253075834	12.85101727	1.392593302	up
ENSG00000095564(BTAF1)	1053	2797	13.481601	29.89503	1.456818542	up
ENSG00000095637(SORBS1)	65	200	0.516600403	1.601461712	1.628408262	up
ENSG00000095752(IL11)	38	164	1.280492399	4.332827454	2.077650519	up
ENSG00000099139(PCSK5)	179	456	1.468394465	3.770019861	1.384648759	up
ENSG00000099250(NRP1)	46	395	0.468505502	3.9063741	3.073844218	up
ENSG00000099622(CIRBP)	4173	1137	219.3884281	63.46073489	-1.82331916	down
ENSG00000099624(ATP5D)	4479	1779	601.562816	228.030232	-1.28079271	down
ENSG00000099795(NDUFB7)	1674	732	396.301	180.221	-1.140383266	down
ENSG00000099800(TIMM13)	2040	787	95.033712	35.2189	-1.321069393	down
ENSG00000099840(IZUMO4)	12	71	0.960104017	5.596753431	2.357489253	up
ENSG00000099953(MMP11)	302	114	8.45514	3.20372	-1.334228023	down
ENSG00000100296(THOC5)	5798	2474	119.7390985	49.66493631	-1.177818859	down
ENSG00000100311(PDGFB)	362	783	6.586504005	14.07459553	1.156616849	up
ENSG00000100364(KIAA0930)	1655	723	25.861243	12.707363	-1.141722993	down
ENSG00000100379(KCTD17)	624	212	19.110808	6.612449245	-1.495212313	down
ENSG00000100439(ABHD4)	274	621	4.287487721	9.805559888	1.221666552	up
ENSG00000100526(CDKN3)	895	372	68.48809808	27.982235	-1.210430267	down
ENSG00000100592(DAAM1)	153	710	1.135866775	5.299822988	2.242778583	up
ENSG00000100600(LGMN)	762	1939	20.19200217	52.49111631	1.394016669	up
ENSG00000100629(CEP128)	256	612	2.28532923	5.6574357	1.297666826	up
ENSG00000100647(SUSD6)	170	722	1.732872142	7.36473	2.117556458	up
ENSG00000100802(C14orf93)	209	59	6.69614323	1.963557099	-1.727533205	down
ENSG00000100979(PLTP)	101	31	4.34162	1.306716	-1.569782351	down
ENSG00000101096(NFATC2)	89	322	0.74630714	2.3815738	1.871276828	up
ENSG00000101220(C20orf27)	2011	882	106.916546	48.45058894	-1.136581186	down
ENSG00000101236(RNF24)	400	1392	2.810313107	10.29014303	1.841515153	up
ENSG00000101246(ARFRP1)	778	333	28.03623357	11.89806142	-1.167529447	down
ENSG00000101333(PLCB4)	79	193	0.89844088	1.3918967	1.307320643	up
ENSG00000101577(LPIN2)	297	682	2.45502107	5.721342136	1.241151085	up
ENSG00000101654(RNMT)	1237	3822	10.58349819	33.76138969	1.675129075	up
ENSG00000101665(SMAD7)	58	403	1.369409648	9.111806	2.786022428	up
ENSG00000101695(RNF125)	251	598	2.21848	5.41966	1.292568514	up
ENSG00000101782(RIOK3)	644	4265	8.326922893	54.43629018	2.771834807	up
ENSG00000101844(ATG4A)	161	395	3.32187993	8.319696791	1.329152674	up
ENSG00000102144(PGK1)	10220	33945	102.8468832	354.0784125	1.781489043	up
ENSG00000102287(GABRE)	45	126	0.672111423	1.629796991	1.476822447	up
ENSG00000102580(DNAJC3)	899	1992	7.43024273	17.0526014	1.195172621	up
ENSG00000102967(DHODH)	536	232	10.79588474	4.720239357	-1.148566652	down
ENSG00000103021(CCDC113)	304	125	2.7907139	1.15205799	-1.213824785	down
ENSG00000103024(NME3)	1290	258	137.46221	28.14529	-2.259566956	down
ENSG00000103089(FA2H)	80	218	2.353797931	8.8060918	1.4631641	up
ENSG00000103202(NME4)	1236	535	88.61728247	39.147295	-1.153923203	down
ENSG00000103253(HAGHL)	791	111	54.40222769	7.37346661	-2.752354476	down
ENSG00000103254(FAM173A)	612	249	50.94813	17.91607	-1.238056255	down
ENSG00000103260(METRN)	3790	1098	310.968	82.22543573	-1.734761509	down
ENSG00000103266(STUB1)	1396	610	65.92326014	30.655639	-1.140812105	down
ENSG00000103351(CLUAP1)	480	197	7.796958984	3.8769408	-1.223140318	down
ENSG00000103811(CTSH)	664	266	21.73385299	10.05826347	-1.260922259	down
ENSG00000103995(CEP152)	799	1793	4.553281352	10.47689075	1.213099156	up
ENSG00000104093(DMXL2)	347	748	6.833165047	12.07766971	1.151441012	up
ENSG00000104267(CA2)	1153	2497	38.383	85.2025	1.162766012	up
ENSG00000104325(DECR1)	1080	461	56.08492945	24.98978549	-1.173327293	down
ENSG00000104419(NDRG1)	1088	15000	20.68130723	301.6619546	3.831604662	up
ENSG00000104450(SPAG1)	293	719	4.21664396	10.20172572	1.336437433	up
ENSG00000104522(TSTA3)	2441	915	107.2487851	39.87736022	-1.362945314	down
ENSG00000104524(PYCRL)	1137	278	28.83744847	6.95695304	-1.971270544	down
ENSG00000104765(BNIP3L)	1015	6287	14.384911	90.69523023	2.67738248	up
ENSG00000104872(PIH1D1)	1724	742	97.53082818	44.89114019	-1.163269744	down
ENSG00000104884(ERCC2)	1738	577	28.17757206	10.25209954	-1.536209902	down
ENSG00000104915(STX10)	2538	1119	121.6735524	56.4250586	-1.129538912	down
ENSG00000105088(OLFM2)	681	242	24.28437	9.33205808	-1.432160198	down
ENSG00000105220(GPI)	12509	36035	326.1079803	976.8047554	1.576185772	up
ENSG00000105339(DENND3)	212	475	3.200180434	6.998319652	1.202681676	up
ENSG00000105364(MRPL4)	3177	1228	175.717887	68.75582103	-1.319399951	down
ENSG00000105516(DBP)	923	228	41.178467	11.17217926	-1.954181637	down
ENSG00000105523(FAM83E)	7	45	0.11692544	0.760784721	2.320298031	up
ENSG00000105641(SLC5A5)	44	4	0.83844508	0.0934061	-2.733905244	down
ENSG00000105668(UPK1A)	10	65	0.648827268	4.025549479	2.442956802	up
ENSG00000105784(RUNDC3B)	67	195	0.855926001	2.480326067	1.550520611	up

ENSG00000106211(HSPB1)	9311	3026	1017.19256	326.8015	-1.570658788	down
ENSG00000106366(SERPINE1)	225	858	3.418181	13.314679	1.967206132	up
ENSG00000106415(GLCCI1)	60	161	0.806897145	2.080852731	1.430639105	up
ENSG00000106477(CEP41)	232	83	2.869629461	0.802025798	-1.402893141	down
ENSG00000106688(SLC1A1)	181	600	2.46777367	8.355718	1.762679169	up
ENSG00000107036(RIC1)	524	1815	3.855123	14.19028229	1.83654911	up
ENSG00000107077(KDM4C)	282	1047	2.96090952	10.70789199	1.931525841	up
ENSG00000107159(CA9)	16	166	0.722094427	6.884171771	3.208127408	up
ENSG00000107338(SHB)	761	2222	15.59262391	40.64378	1.592183954	up
ENSG00000107833(NPM3)	2666	1169	209.0983	95.02787	-1.137534039	down
ENSG00000107864(CPEB3)	50	172	0.395482573	1.396103807	1.774149246	up
ENSG00000107872(FBXL15)	805	349	70.2575742	30.6917142	-1.149416696	down
ENSG00000107874(CUEDC2)	2613	1027	124.75	49.8721	-1.294956533	down
ENSG00000107957(SH3PXD2A)	199	878	0.856467811	3.922536534	2.175093519	up
ENSG00000107984(DKK1)	14547	2940	510.292953	105.188448	-2.255766802	down
ENSG00000108175(ZMIZ1)	601	1261	8.58015118	17.52866739	1.115350962	up
ENSG00000108244(KRT23)	726	128	17.03018543	3.021034	-2.428276638	down
ENSG00000108379(WNT3)	118	365	1.7887804	5.33787	1.655002907	up
ENSG00000108387(SEPT4)	117	30	3.54260641	0.995562067	-1.819711406	down
ENSG00000108528(SLC25A11)	1376	535	56.79871	20.98215994	-1.308380049	down
ENSG00000108641(B9D1)	318	133	26.547177	11.483182	-1.190589728	down
ENSG00000109046(WSB1)	997	6690	12.45887884	100.7830308	2.792717925	up
ENSG00000109107(ALDOC)	1652	8531	51.49744705	285.5052872	2.416413484	up
ENSG00000109220(CHIC2)	147	460	6.20761913	22.94483702	1.676299255	up
ENSG00000109321(AREG)	1347	3107	53.39750068	126.086777	1.253939786	up
ENSG00000109511(ANXA10)	462	97	21.24068476	4.654094	-2.169699655	down
ENSG00000109667(SLC2A9)	5	37	0.176031415	1.3234789	2.38131305	up
ENSG00000109756(RAPGEF2)	319	827	2.044200828	6.392611143	1.416100239	up
ENSG00000109762(SNX25)	282	606	3.665045	8.095206688	1.145462982	up
ENSG00000109771(LRP2BP)	122	1072	1.37155621	10.34643734	3.155093043	up
ENSG00000110169(HPX)	11	59	0.372435129	1.978291393	2.201672816	up
ENSG00000110324(IL10RA)	23	95	0.323660483	1.356214	1.966441065	up
ENSG00000110422(HIPK3)	689	1796	4.82152755	12.77202747	1.42836303	up
ENSG00000110442(COMMD9)	397	168	12.51763	4.344040537	-1.177296258	down
ENSG00000110811(P3H3)	795	314	18.553091	7.8965341	-1.282626115	down
ENSG00000110881(ASIC1)	1080	338	15.139901	4.68303217	-1.617891943	down
ENSG00000110888(CAPRIN2)	483	1499	6.11650309	16.458594	1.677917552	up
ENSG00000111145(ELK3)	1235	3518	15.33188481	44.1649563	1.557977388	up
ENSG00000111653(ING4)	251	78	9.785088198	2.657036587	-1.601712445	down
ENSG00000111671(SPSB2)	187	66	10.41845315	3.28407925	-1.414623249	down
ENSG00000111700(SLCO1B3)	49	569	0.852353787	10.64874145	3.511416825	up
ENSG00000111801(BTN3A3)	171	62	2.116902489	0.932017643	-1.374048592	down
ENSG00000111859(NEDD9)	6	41	0.076034424	0.50111258	2.347700758	up
ENSG00000111860(CEP85L)	100	299	0.657568627	2.00009029	1.602233324	up
ENSG00000111875(ASF1A)	763	276	22.32818	9.0371008	-1.407909681	down
ENSG00000111897(SERINC1)	936	2641	14.62459	43.21994195	1.543536162	up
ENSG00000112081(SRSF3)	9990	3710	222.1412371	82.89880218	-1.378416419	down
ENSG00000112139(MDGA1)	50	184	0.890201178	1.323838181	1.869932455	up
ENSG00000112182(BACH2)	25	136	0.141365766	1.331075526	2.364221457	up
ENSG00000112304(ACOT13)	671	284	7.369511	3.186318	-1.182478064	down
ENSG00000112379(ARFGEF3)	120	1519	0.380905735	4.9560405	3.680085659	up
ENSG00000112406(HECA)	247	809	2.30361	7.81233	1.749709442	up
ENSG00000112667(DNPH1)	1752	584	230.4234	79.707416	-1.530452123	down
ENSG00000112715(VEGFA)	1054	3047	18.4139583	51.1891347	1.578843265	up
ENSG00000112972(HMGCS1)	1704	3737	24.78329	55.466561	1.181554563	up
ENSG00000112984(KIF20A)	1677	532	23.55638	7.41885	-1.601305715	down
ENSG00000113070(HBEGF)	204	1529	4.247284355	32.46189378	2.938136214	up
ENSG00000113119(TMCO6)	402	117	13.323024	4.001297	-1.706861113	down
ENSG00000113369(ARRDC3)	341	1097	4.403909508	14.18123425	1.727127469	up
ENSG00000113578(FGF1)	1	49	0.105943	0.867482009	3.777811017	up
ENSG00000113615(SEC24A)	540	1546	4.25741	13.02842	1.56235161	up
ENSG00000113739(STC2)	992	5668	11.495526	61.48488	2.56089296	up
ENSG00000113742(CPEB4)	257	633	1.96068508	4.968291323	1.340560349	up
ENSG00000113790(EHHADH)	207	67	2.812016769	0.872470154	-1.538262851	down
ENSG00000113916(BCL6)	201	626	2.87429132	9.028720442	1.674667645	up
ENSG00000114023(FAM162A)	884	3836	33.96202842	161.6095899	2.163775689	up
ENSG00000114166(KAT2B)	85	350	1.084825585	4.012317064	2.054765901	up
ENSG00000114268(PFKFB4)	415	3712	6.42643486	57.02364273	3.201999439	up
ENSG00000114331(ACAP2)	726	1639	4.869299373	11.40865554	1.221451265	up
ENSG00000114480(GBE1)	568	2822	9.34932	47.57653	2.356794463	up
ENSG00000114646(CSPG5)	67	179	1.625205648	4.343932243	1.428837722	up
ENSG00000114698(PLSCR4)	29	111	0.550363158	1.993027371	1.884517496	up
ENSG00000114767(RRP9)	2203	815	76.9593	28.8228	-1.381557668	down
ENSG00000114771(AADAC)	83	440	2.378696632	8.547411	2.415762071	up
ENSG00000115112(TFCP2L1)	272	898	1.63967	5.494	1.762239444	up
ENSG00000115234(SNX17)	2531	894	69.12516	24.98798	-1.448512537	down

ENSG00000115255(REEP6)	1680	706	77.43783216	31.84560459	-1.197508228	down
ENSG00000115274(INO80B)	1167	440	55.02970201	18.18085558	-1.351657354	down
ENSG00000115339(GALNT3)	441	979	7.785838117	16.8115608	1.195153509	up
ENSG00000115548(KDM3A)	594	5177	5.184259794	45.840492	3.167289444	up
ENSG00000115641(FHL2)	1533	3725	60.96568475	149.6416445	1.32921118	up
ENSG00000115756(HPCAL1)	1272	3042	59.91287944	140.2512987	1.305933352	up
ENSG00000115844(DLX2)	233	542	7.871196836	17.93841314	1.257493637	up
ENSG00000115875(SRSF7)	4382	1554	155.41501	42.797429	-1.443976723	down
ENSG00000115902(SLC1A4)	114	399	1.334445845	4.704421995	1.831090212	up
ENSG00000116016(EPAS1)	688	2107	7.179388	22.01407	1.660519731	up
ENSG00000116017(ARID3A)	410	1295	4.899551849	13.59617012	1.702156139	up
ENSG00000116106(EPHA4)	44	194	0.6288696	1.572268127	2.118806804	up
ENSG00000116260(QSOX1)	2018	6927	21.81386688	89.49211325	1.827787118	up
ENSG00000116285(ERRFI1)	7009	19160	113.27148	312.624961	1.50040622	up
ENSG00000116514(RNF19B)	560	1858	9.733337307	29.607156	1.774930799	up
ENSG00000116586(LAMTOR2)	710	252	98.277673	34.940021	-1.43431455	down
ENSG00000116649(SRM)	2905	998	178.669204	59.162265	-1.488836233	down
ENSG00000116704(SLC35D1)	196	439	1.739408121	4.056532972	1.201287574	up
ENSG00000116711(PLA2G4A)	277	93	4.765787762	1.596671	-1.496719573	down
ENSG00000116791(CRYZ)	632	188	20.907358	6.271209256	-1.68442922	down
ENSG00000116977(LGALS8)	512	1431	6.576298	25.98702878	1.527438096	up
ENSG00000116991(SIPA1L2)	169	528	1.686511406	4.690346797	1.676518003	up
ENSG00000117000(RLF)	677	2133	4.48119055	14.444198	1.701343935	up
ENSG00000117036(ETV3)	551	1303	4.895922	12.500617	1.287198182	up
ENSG00000117143(UAP1)	1232	2867	26.2935755	62.18663	1.266525897	up
ENSG00000117280(RAB29)	646	241	11.29841656	4.4276501	-1.362277328	down
ENSG00000117318(ID3)	4383	1151	270.839	71.3973	-1.876492445	down
ENSG00000117394(SLC2A1)	3098	11777	51.34147576	203.459545	1.975529113	up
ENSG00000117399(CDC20)	7428	1858	246.5886	62.32565	-1.94763066	down
ENSG00000117525(F3)	218	651	4.868003	14.67460392	1.615388891	up
ENSG00000117595(IRF6)	472	1387	4.97854	15.191577	1.599137978	up
ENSG00000117877(CD3EAP)	511	214	8.20733	3.436194	-1.195099908	down
ENSG00000118263(KLF7)	283	740	2.34184547	6.101331712	1.42740874	up
ENSG00000118276(B4GALT6)	193	443	2.193033886	5.132383001	1.236215146	up
ENSG00000118418(HMGN3)	1087	435	71.02649	29.777771	-1.265851704	down
ENSG00000118420(UBE3D)	239	79	7.116055611	2.323502913	-1.514122732	down
ENSG00000118503(TNFAIP3)	43	199	0.552970912	2.108652706	2.186087396	up
ENSG00000118515(SGK1)	40	229	0.972515	5.499147233	2.483524578	up
ENSG00000118523(CTGF)	50	162	1.24255	4.04383	1.689166028	up
ENSG00000118620(ZNF430)	225	491	2.864668763	5.443332517	1.165469131	up
ENSG00000118689(FOXO3)	864	2165	6.29443218	16.05089203	1.372260731	up
ENSG00000118804(STBD1)	398	844	6.1143944	13.6115455	1.128736911	up
ENSG00000118894(EEF2KMT)	805	351	20.48230231	8.315718033	-1.141238395	down
ENSG00000118898(PPL)	1874	4589	19.39008	42.31504672	1.340679991	up
ENSG00000118922(KLF12)	191	442	0.843120627	2.01855588	1.247779195	up
ENSG00000118965(WDR35)	566	246	2.879024408	1.349787235	-1.143172535	down
ENSG00000118985(ELL2)	400	1956	3.238219005	18.12541989	2.331423581	up
ENSG00000119411(BSPRY)	490	1058	11.5453	25.4893	1.155746997	up
ENSG00000119508(NR4A3)	8	302	0.091858725	3.2131727	4.854100446	up
ENSG00000119574(ZBTB45)	346	104	13.12025878	3.379085219	-1.657814594	down
ENSG00000119699(TGFB3)	25	107	0.47853632	1.7084705	2.026123324	up
ENSG00000119801(YPEL5)	336	858	8.165678848	21.54448399	1.394769251	up
ENSG00000119929(CUTC)	1061	409	43.4681	17.585142	-1.319344572	down
ENSG00000119986(AVPI1)	616	1398	25.6423	58.5684	1.228436465	up
ENSG00000120051(CFAP58)	4	39	0.045517043	0.6432541	2.647005745	up
ENSG00000120129(DUSP1)	223	1026	8.08409	37.0203	2.237131632	up
ENSG00000120217(CD274)	18	301	0.28626822	4.320105065	3.910851123	up
ENSG00000120458(MSANTD2)	410	1008	9.447531991	24.681251	1.341589246	up
ENSG00000120471(TP53AIP1)	7	44	0.123195242	1.546418824	2.289803314	up
ENSG00000120519(SLC10A7)	67	185	1.308181523	2.903597753	1.475679687	up
ENSG00000120549(KIAA1217)	193	593	1.320728554	3.837715758	1.654637339	up
ENSG00000120868(APAF1)	221	607	1.801513077	4.679915813	1.495417297	up
ENSG00000120899(PTK2B)	235	555	3.487056185	6.918521307	1.279334851	up
ENSG00000120915(EPHX2)	390	98	9.308738557	4.018165259	-1.912565106	down
ENSG00000121621(KIF18A)	1412	618	19.92329	7.82075	-1.138507325	down
ENSG00000121743(GJA3)	375	945	4.43446	10.68024173	1.376531819	up
ENSG00000121966(CXCR4)	4	83	0.160710046	3.276403396	3.684278147	up
ENSG00000122378(FAM213A)	257	90	4.731975443	1.782110002	-1.435664181	down
ENSG00000122591(FAM126A)	494	1064	6.702555148	11.9572115	1.152223646	up
ENSG00000122592(HOXA7)	539	221	16.661312	6.846965282	-1.225794476	down
ENSG00000122877(EGR2)	28	245	0.5461179	4.704333265	3.053048939	up
ENSG00000122884(P4HA1)	581	7645	10.1436869	137.48072	3.761204193	up
ENSG00000123240(OPTN)	425	1253	6.662819	18.5383475	1.60321524	up
ENSG00000123358(NR4A1)	505	1722	12.66235372	46.50523121	1.813768831	up
ENSG00000123364(HOXC13)	103	27	3.201612	0.833295	-1.778931966	down
ENSG00000123384(LRP1)	238	860	0.8833543	3.362171	1.890524314	up

ENSG00000123405(NFE2)	36	2	1.1908258	0.091299591	-2.965645854	down
ENSG00000123416(TUBA1B)	34751	13674	1275.003206	515.8776249	-1.295466922	down
ENSG00000123453(SARDH)	253	99	4.973308507	1.992297719	-1.279763468	down
ENSG00000123612(ACVR1C)	9	80	0.056270826	0.492079155	2.847568853	up
ENSG00000123836(PFKFB2)	255	704	2.201633561	7.615110276	1.504431174	up
ENSG00000124006(OBSL1)	195	54	2.0558907	0.606931834	-1.750618721	down
ENSG00000124145(SDC4)	1618	5975	35.31285	134.7489	1.932789402	up
ENSG00000124225(PMEPA1)	22	149	0.902363176	2.567908935	2.65742931	up
ENSG00000124466(LYPD3)	102	374	4.28263	15.592	1.894659724	up
ENSG00000124507(PACSIN1)	204	67	2.404780127	0.802170508	-1.517495379	down
ENSG00000124766(SOX4)	9621	2021	98.0275	21.1357	-2.199571883	down
ENSG00000124787(RPP40)	383	153	19.457299	8.0852875	-1.258488845	down
ENSG00000124788(ATXN1)	118	625	1.24271296	6.309660695	2.426367885	up
ENSG00000124813(RUNX2)	24	80	0.552706744	1.485778187	1.671635635	up
ENSG00000124942(AHNAK)	4528	12386	22.37293498	38.44557866	1.50114135	up
ENSG00000125089(SH3TC1)	82	195	1.158082886	3.277502499	1.270086904	up
ENSG00000125246(CLYBL)	276	96	11.2202202	4.590387292	-1.447076056	down
ENSG00000125266(EFNB2)	385	1894	4.366473	21.99692704	2.339756894	up
ENSG00000125378(BMP4)	14578	4270	488.6240109	142.9312282	-1.720846786	down
ENSG00000125534(PPDPF)	2588	1121	330.13132	142.32925	-1.155082741	down
ENSG00000125629(INSIG2)	200	2053	3.72329735	44.85564442	3.390768776	up
ENSG00000125740(FOSB)	84	833	1.301947	13.455391	3.315551083	up
ENSG00000125775(SDCBP2)	22	111	1.000438801	4.523524989	2.241794354	up
ENSG00000125901(MRPS26)	1650	658	101.766	41.0714	-1.27272161	down
ENSG00000125968(ID1)	6523	2512	564.7135	214.9723	-1.325752508	down
ENSG00000126218(F10)	77	17	2.854433	0.648052711	-1.962075967	down
ENSG00000126246(IGFLR1)	102	25	6.271367	1.890352732	-1.865433068	down
ENSG00000126524(SBDS)	939	2168	28.34472338	67.12211566	1.254548953	up
ENSG00000126562(WNK4)	32	138	0.452814536	1.94129803	2.062006792	up
ENSG00000126787(DLGAP5)	1705	695	22.181585	9.25780628	-1.241346788	down
ENSG00000126804(ZBTB1)	374	858	4.929924	11.69628	1.241451612	up
ENSG00000127081(ZNF484)	251	601	1.222198741	1.840587144	1.299754321	up
ENSG00000127124(HIVEP3)	186	499	1.996767165	3.46414811	1.459418694	up
ENSG00000127152(BCL11B)	33	110	0.263470903	0.8868506	1.701410914	up
ENSG00000127528(KLF2)	501	1150	26.16514349	58.00964539	1.243893513	up
ENSG00000127603(MACF1)	1339	2869	13.1195464	20.92195152	1.147649012	up
ENSG00000127663(KDM4B)	678	3642	6.960978	36.722769	2.470283503	up
ENSG00000128011(LRFN1)	800	296	20.117634	6.865946444	-1.376027415	down
ENSG00000128310(GALR3)	1	27	0.111405	2.96748	2.980406239	up
ENSG00000128510(CPA4)	110	1226	2.07726841	22.53253281	3.493988079	up
ENSG00000128590(DNAJB9)	285	889	5.88674	19.085	1.681094615	up
ENSG00000128694(OSGEPL1)	217	75	6.14322043	2.029173786	-1.448886172	down
ENSG00000128917(DLL4)	27	129	0.457675	2.20524	2.189726614	up
ENSG00000128923(MINDY2)	575	1458	3.0086828	7.815043	1.387828373	up
ENSG00000128944(KNSTRN)	1252	508	39.14896771	16.05137903	-1.246745317	down
ENSG00000128965(CHAC1)	167	598	7.30100563	26.02138632	1.872113477	up
ENSG00000129173(E2F8)	429	904	6.514295636	13.71416163	1.120046564	up
ENSG00000129226(CD68)	114	523	3.3081387	15.464854	2.21911711	up
ENSG00000129355(CDKN2D)	238	512	13.00869	28.2773	1.145523113	up
ENSG00000129514(FOXA1)	18	133	0.436207	3.10994	2.749279891	up
ENSG00000129521(EGLN3)	149	642	3.161913723	13.382898	2.135631923	up
ENSG00000129646(QRICH2)	112	491	1.974988416	7.398845045	2.153425707	up
ENSG00000129757(CDKN1C)	231	784	11.2352096	40.032495	1.800037587	up
ENSG00000130005(GAMT)	2223	784	141.9355052	51.03549857	-1.450329306	down
ENSG00000130052(STARD8)	183	67	2.964260897	0.882522704	-1.363096797	down
ENSG00000130147(SH3BP4)	986	2101	16.05459382	29.02373027	1.139069967	up
ENSG00000130294(KIF1A)	10	52	0.082631408	0.352818127	2.135905119	up
ENSG00000130303(BST2)	54	11	3.46992	0.731547	-1.992856207	down
ENSG00000130347(RTN4IP1)	244	85	7.77314	2.834430001	-1.441531207	down
ENSG00000130449(ZSWIM6)	296	812	4.376047	10.219984	1.496728977	up
ENSG00000130513(GDF15)	3106	11639	245.7356535	903.742628	1.954811025	up
ENSG00000130589(HELZ2)	598	1637	5.196558	14.46659	1.49828429	up
ENSG00000130675(MNX1)	126	309	6.843050747	13.95289494	1.324280187	up
ENSG00000130766(SESN2)	447	1523	7.3581	25.2396	1.811843114	up
ENSG00000130783(CCDC62)	32	163	0.4435556	2.067732	2.297785804	up
ENSG00000130811(EIF3G)	5028	2011	257.417171	102.70241	-1.270914459	down
ENSG00000130827(PLXNA3)	960	2449	4.966643	12.5773	1.398350841	up
ENSG00000130881(LRP3)	1884	696	51.5522844	18.4374166	-1.383074428	down
ENSG00000131015(ULBP2)	123	784	5.534317	35.09530379	2.693595456	up
ENSG00000131069(ACSS2)	943	2005	13.77081789	29.83853055	1.13582462	up
ENSG00000131094(C1QL1)	124	42	9.99679	3.45037	-1.451896559	down
ENSG00000131378(RFTN1)	97	236	1.578553685	3.7565049	1.307139738	up
ENSG00000131389(SLC6A6)	917	2038	7.625573689	16.31766875	1.199553903	up
ENSG00000131711(MAP1B)	457	1644	2.045772456	5.579705	1.890226709	up
ENSG00000131791(PRKAB2)	400	865	3.888028202	8.451958	1.156898556	up
ENSG00000131979(GCH1)	544	1435	13.7700639	32.0963125	1.44447421	up

ENSG00000132004(FBXW9)	294	107	11.980621	4.3444106	-1.385023527	down
ENSG00000132128(LRRC41)	1322	576	27.63568	10.4477541	-1.144753143	down
ENSG00000132326(PER2)	274	715	2.122121733	5.559557	1.42416146	up
ENSG00000132330(SCLY)	640	230	21.96877413	8.543967	-1.415479603	down
ENSG00000132334(PTPRE)	256	704	2.879598254	8.135034162	1.498848209	up
ENSG00000132382(MYBBP1A)	2782	1200	34.38946781	14.24328388	-1.16124402	down
ENSG00000132394(EEFSEC)	543	229	14.19582	5.951079	-1.185736334	down
ENSG00000132423(COQ3)	422	116	18.17706478	5.234698903	-1.788508961	down
ENSG00000132600(PRMT7)	623	268	19.96023982	7.305457773	-1.15869133	down
ENSG00000132622(HSPA12B)	51	10	1.166493279	0.234179458	-2.02315885	down
ENSG00000132763(MMACHC)	527	190	8.16507	1.955508623	-1.408581727	down
ENSG00000132768(DPH2)	1437	430	33.58006713	10.2186965	-1.684192186	down
ENSG00000132801(ZSWIM3)	30	125	0.592775	2.405504	2.007372209	up
ENSG00000132906(CASP9)	205	477	4.868841458	11.7409098	1.256481181	up
ENSG00000133069(TMCC2)	81	278	1.271998459	4.544152266	1.792654379	up
ENSG00000133083(DCLK1)	20	310	0.210068499	3.336281345	3.821186998	up
ENSG00000133131(MORC4)	550	1233	5.7871847	13.59412701	1.210317147	up
ENSG00000133138(TBC1D8B)	296	651	2.444077712	5.202960802	1.179137358	up
ENSG00000133265(HSPBP1)	2048	897	85.86869566	39.80755086	-1.138593481	down
ENSG00000133315(MACROD1)	1109	447	68.97564	27.77633	-1.255671998	down
ENSG00000133316(WDR74)	1874	790	86.96199037	36.55528896	-1.193333735	down
ENSG00000133627(ACTR3B)	452	192	11.97106788	5.361127	-1.173528013	down
ENSG00000133639(BTG1)	529	2149	6.120811546	25.83980681	2.066272629	up
ENSG00000133739(LRRCC1)	575	178	5.692320376	1.799155171	-1.626383785	down
ENSG00000133794(ARNTL)	216	544	3.342331186	12.32713581	1.370678529	up
ENSG00000133816(MICAL2)	574	1426	4.26002028	10.7837654	1.358372502	up
ENSG00000133943(C14orf159)	587	239	12.49317515	6.944143933	-1.236637234	down
ENSG00000133985(TTC9)	59	221	0.566897906	2.154082783	1.904003498	up
ENSG00000134013(LOXL2)	1572	5445	18.77625	70.21160648	1.84038337	up
ENSG00000134057(CCNB1)	5443	1043	150.0481296	27.94767703	-2.330574061	down
ENSG00000134107(BHLHE40)	629	5823	9.90525	92.2653	3.254643268	up
ENSG00000134109(EDEM1)	397	1285	3.49587851	11.50716098	1.737148663	up
ENSG00000134198(TSPAN2)	41	1	0.641840716	0.031265537	-3.476185363	down
ENSG00000134222(PSRC1)	936	304	27.73370031	9.075568726	-1.563658973	down
ENSG00000134317(GRHL1)	112	255	2.255795261	4.69150171	1.21578171	up
ENSG00000134324(LPIN1)	449	1597	6.61825193	22.28778181	1.873769263	up
ENSG00000134330(IAH1)	394	161	19.05333568	8.16482	-1.226801318	down
ENSG00000134333(LDHA)	23088	52779	521.5117695	1226.157064	1.242692274	up
ENSG00000134461(ANKRD16)	317	99	9.057423702	3.206455	-1.601792438	down
ENSG00000134531(EMP1)	614	3072	6.691848295	45.35936335	2.367341235	up
ENSG00000134533(RERG)	186	30	4.13629608	0.806257853	-2.475456173	down
ENSG00000134545(KLRC1)	144	33	9.857006	2.285002	-1.986469209	down
ENSG00000134548(SPX)	0	108	0	2.650964825	5.300657688	up
ENSG00000134574(DDB2)	736	1552	20.64147692	44.82506948	1.123244502	up
ENSG00000134755(DSC2)	381	897	3.139464209	7.43319326	1.27882904	up
ENSG00000134769(DTNA)	154	34	3.512533907	0.798733763	-2.041739911	down
ENSG00000134954(ETS1)	145	1181	1.416617839	11.31857127	3.050619102	up
ENSG00000134970(TMED7)	2369	6217	37.32210564	101.4461495	1.440797725	up
ENSG00000135063(FAM189A2)	37	190	1.286277013	6.118479236	2.322325739	up
ENSG00000135119(RNFT2)	567	246	5.60494654	3.031525	-1.1457062	down
ENSG00000135127(BICDL1)	410	873	6.2933666	13.73141537	1.134767546	up
ENSG00000135164(DMTF1)	533	1259	7.761045752	16.60526476	1.285408733	up
ENSG00000135241(PNPLA8)	423	1296	5.622518729	17.32002502	1.65854616	up
ENSG00000135245(HILPDA)	877	2273	21.02434	50.73429	1.420928757	up
ENSG00000135318(NT5E)	174	691	2.88051	11.66754531	2.021500167	up
ENSG00000135373(EHF)	328	97	2.811547641	0.86442329	-1.679187753	down
ENSG00000135480(KRT7)	478	176	15.78473101	6.556663001	-1.377353064	down
ENSG00000135540(NHSL1)	233	514	1.62092715	3.595722	1.181374644	up
ENSG00000135604(STX11)	16	97	0.4235845	2.999682	2.449991772	up
ENSG00000135679(MDM2)	3278	7369	15.20629932	38.06218	1.21789939	up
ENSG00000135709(KIAA0513)	280	638	2.627889142	5.62875895	1.229530486	up
ENSG00000135766(EGLN1)	1106	5394	15.79106	72.318	2.332940784	up
ENSG00000135842(FAM129A)	75	279	0.485361623	1.97941323	1.904816385	up
ENSG00000135913(USP37)	638	1367	3.973563245	8.990391748	1.145758902	up
ENSG00000135924(DNAJB2)	487	1619	13.14971786	44.5115	1.776992356	up
ENSG00000136068(FLNB)	2884	7426	30.6934672	54.25270624	1.413581483	up
ENSG00000136114(THSD1)	46	133	0.685752036	1.998702551	1.523364408	up
ENSG00000136167(LCP1)	29	156	0.337641169	1.8858	2.365186785	up
ENSG00000136327(NKX2-8)	120	34	11.1621	3.1485	-1.689406257	down
ENSG00000136463(TACO1)	975	428	33.9353	15.255	-1.132669469	down
ENSG00000136492(BRIP1)	656	1386	7.354847215	12.73400304	1.125672716	up
ENSG00000136603(SKIL)	311	1027	2.099762996	7.324683099	1.763931337	up
ENSG00000136718(IMP4)	1637	677	106.331474	40.49402946	-1.220434104	down
ENSG00000136770(DNAJC1)	500	1149	10.8025	25.1748	1.245507355	up
ENSG00000136802(LRRC8A)	905	1972	13.947772	29.23605	1.171069337	up
ENSG00000136826(KLF4)	490	1499	10.57842241	32.22839869	1.657282447	up

ENSG00000136868(SLC31A1)	1032	2489	10.6021	26.3288	1.317665321	up
ENSG00000136997(MYC)	7736	2950	204.13843	75.02531	-1.340064779	down
ENSG00000137193(PIM1)	445	1611	10.82384182	40.27344	1.899166002	up
ENSG00000137261(KIAA0319)	21	84	0.171929212	0.766044242	1.91021543	up
ENSG00000137274(BPHL)	553	193	16.90397093	6.783236501	-1.455411238	down
ENSG00000137288(UQCC2)	694	294	73.4681	28.37549	-1.181450318	down
ENSG00000137440(FGFBP1)	219	75	7.647586	2.634642974	-1.461947613	down
ENSG00000137449(CPEB2)	14	140	0.313201895	1.316425402	3.128094197	up
ENSG00000137460(FHDC1)	85	228	0.699133172	1.86564295	1.442583275	up
ENSG00000137496(IL18BP)	105	270	2.465016	5.685876693	1.388044929	up
ENSG00000137502(RAB30)	87	257	0.466796555	1.843243534	1.580879399	up
ENSG00000137571(SLCO5A1)	2	33	0.022400834	0.263708279	2.916446148	up
ENSG00000137700(SLC37A4)	1383	539	41.707665	16.78745789	-1.304994911	down
ENSG00000137801(THBS1)	197	574	1.133046519	3.83798674	1.578721769	up
ENSG00000137825(ITPKA)	233	610	9.673314705	24.3997313	1.427179872	up
ENSG00000137868(STRA6)	7	41	0.253837189	1.036997035	2.194249786	up
ENSG00000137877(SPTBN5)	36	132	0.270357008	0.9222898	1.842686795	up
ENSG00000137936(BCAR3)	335	1108	5.954891904	17.42045524	1.766877192	up
ENSG00000138028(CGREF1)	111	33	3.067455129	0.882812976	-1.619432518	down
ENSG00000138074(SLC5A6)	1272	398	26.10214213	8.252079047	-1.619423968	down
ENSG00000138085(ATRAID)	1547	619	96.2832626	37.603358	-1.267659417	down
ENSG00000138271(GPR87)	75	292	2.912889	11.24704	1.969878717	up
ENSG00000138356(AOX1)	1	41	0.015080173	0.598077279	3.53584793	up
ENSG00000138448(ITGAV)	802	1804	5.970532	13.67546	1.216522087	up
ENSG00000138623(SEMA7A)	405	1451	6.512211141	24.18904492	1.883491975	up
ENSG00000138641(HERC3)	95	567	1.37121889	5.28273847	2.590925115	up
ENSG00000138678(GPAT3)	353	1843	6.583445259	35.74217635	2.424645128	up
ENSG00000138764(CCNG2)	361	1211	4.551163257	17.67485472	1.787865087	up
ENSG00000138777(PPA2)	1490	608	54.48604086	26.48897433	-1.239352264	down
ENSG00000138778(CENPE)	1537	613	6.45464717	2.763348709	-1.272309502	down
ENSG00000139132(FGD4)	177	422	1.250274297	2.715200449	1.289519439	up
ENSG00000139174(PRICKLE1)	23	167	0.233450335	1.593600989	2.762299651	up
ENSG00000139496(NUP58)	2296	6086	25.80228937	69.34732	1.455186788	up
ENSG00000139531(SUOX)	512	182	11.43915555	4.318487735	-1.428291297	down
ENSG00000139687(RB1)	781	2269	11.341652	29.7852	1.585058656	up
ENSG00000139734(DIAPH3)	845	1853	10.251821	20.09440525	1.180042654	up
ENSG00000139946(PELI2)	77	192	0.705464742	1.780834	1.335597294	up
ENSG00000140009(ESR2)	5	42	0.0493686	0.4418449	2.551997114	up
ENSG00000140057(AK7)	76	9	1.380300722	0.182368818	-2.690057445	down
ENSG00000140451(PIF1)	502	72	8.623767838	1.183665595	-2.704720636	down
ENSG00000140470(ADAMTS17)	29	110	0.3443383	1.752754779	1.871785291	up
ENSG00000140854(KATNB1)	1245	550	27.33000669	11.8142568	-1.124678672	down
ENSG00000140941(MAP1LC3B)	1707	6575	35.901081	140.73972	1.993663286	up
ENSG00000140950(TLDC1)	537	1130	5.69167973	12.36485038	1.119091945	up
ENSG00000140990(NDUFB10)	3058	902	410.93916	122.914219	-1.708295339	down
ENSG00000141086(CTRL)	87	18	3.1995	0.843677	-2.061137361	down
ENSG00000141255(SPATA22)	39	114	0.3370466	1.027195943	1.528773291	up
ENSG00000141295(SCRN2)	1197	282	45.18593909	10.99147246	-2.024871401	down
ENSG00000141314(RHBDL3)	157	382	2.072837597	4.269327001	1.316863195	up
ENSG00000141376(BCAS3)	353	134	5.351100499	1.83064684	-1.329349353	down
ENSG00000141449(GREB1L)	111	391	0.593164165	2.22055975	1.839580354	up
ENSG00000141458(NPC1)	752	2521	11.76307472	38.975124	1.791203022	up
ENSG00000141522(ARHGDIA)	8749	2733	284.9468023	90.62749923	-1.627652721	down
ENSG00000141562(NARF)	1419	3421	44.149162	116.3179318	1.317749031	up
ENSG00000141682(PMAIP1)	965	3389	28.01750428	102.818143	1.859086838	up
ENSG00000141741(MIEN1)	1061	390	77.90693	29.96353	-1.387491535	down
ENSG00000141934(PLPP2)	2115	899	117.408932	49.24739399	-1.181767467	down
ENSG00000142089(IFITM3)	4848	2093	524.1310104	231.2580079	-1.160770179	down
ENSG00000142619(PADI3)	72	9	1.343945786	0.189082115	-2.615008144	down
ENSG00000142627(EPHA2)	3503	7784	72.36823	152.04799	1.201219985	up
ENSG00000142694(EVA1B)	508	206	50.447708	20.261943	-1.240893496	down
ENSG00000142867(BCL10)	472	1206	9.979063539	28.0894942	1.397838035	up
ENSG00000142871(CYR61)	297	1130	7.446666737	28.89850812	1.967280124	up
ENSG00000142875(PRKACB)	2653	1010	34.5495213	12.61593873	-1.340864479	down
ENSG00000142910(TINAGL1)	821	2961	23.3172	80.762531	1.89687055	up
ENSG00000143061(IGSF3)	383	1090	2.749448097	7.730272281	1.551682641	up
ENSG00000143067(ZNF697)	206	478	2.297936171	5.370084	1.252562463	up
ENSG00000143217(NECTIN4)	29	219	0.443027194	3.462193	2.847205739	up
ENSG00000143314(MRPL24)	1690	534	125.8189425	40.95386346	-1.607041954	down
ENSG00000143367(TUFT1)	306	938	5.050725743	15.31877298	1.656693017	up
ENSG00000143369(ECM1)	61	233	1.669517329	6.3662325	1.933484588	up
ENSG00000143494(VASH2)	110	34	1.448188232	0.420893451	-1.566977775	down
ENSG00000143631(FLG)	0	53	0	0.1575	4.311183532	up
ENSG00000143669(LYST)	92	299	0.306941701	1.009044783	1.71898387	up
ENSG00000143816(WNT9A)	85	1473	1.27690112	22.47784752	4.119305083	up
ENSG00000143847(PPFIA4)	124	504	1.133052189	4.805933401	2.047613769	up

ENSG00000144120(TMEM177)	689	284	42.0591477	16.7888968	-1.220506492	down
ENSG00000144228(SPOPL)	767	1859	6.06456	15.47735	1.323920728	up
ENSG00000144320(LNPK)	569	1279	3.828512	9.369847767	1.214299143	up
ENSG00000144354(CDCA7)	1525	519	32.79681898	11.5785056	-1.499983135	down
ENSG00000144357(UBR3)	691	1544	5.627332288	11.79532254	1.206452242	up
ENSG00000144395(CCDC150)	313	736	4.194539349	8.627686005	1.27566664	up
ENSG00000144426(NBEAL1)	282	599	1.040152135	2.064339033	1.128779045	up
ENSG00000144452(ABCA12)	13	72	0.0713436	0.365442325	2.283253362	up
ENSG00000144559(TAMM41)	165	51	8.567478162	2.329074	-1.591731622	down
ENSG00000144560(VGLL4)	744	1754	12.29360076	28.5460444	1.283928479	up
ENSG00000144645(OSBPL10)	358	858	2.62621391	8.334872485	1.304032477	up
ENSG00000144655(CSRNP1)	397	1769	6.951453	31.26784	2.197452372	up
ENSG00000144671(SLC22A14)	5	37	0.136900043	1.003795288	2.38131305	up
ENSG00000144747(TMF1)	1141	2657	11.34767072	20.84719568	1.267326226	up
ENSG00000144821(MYH15)	551	2332	2.4528226	8.655435557	2.125557642	up
ENSG00000144959(NCEH1)	291	882	3.347053782	10.45572	1.639969454	up
ENSG00000145362(ANK2)	50	141	0.482112401	1.420998557	1.492528076	up
ENSG00000145390(USP53)	560	1554	4.01967026	11.58781241	1.517593143	up
ENSG00000145623(OSMR)	909	3047	9.324543	32.15595	1.791732329	up
ENSG00000145779(TNFAIP8)	342	947	9.196273918	27.27108355	1.511395422	up
ENSG00000145780(FEM1C)	509	2057	5.15381	21.31092	2.058525157	up
ENSG00000145901(TNIP1)	607	1717	10.3202993	28.48744613	1.545557336	up
ENSG00000145911(N4BP3)	368	798	2.925928	6.192902122	1.160367819	up
ENSG00000145912(NHP2)	4092	1549	353.41126	137.61937	-1.349902197	down
ENSG00000145990(GFOD1)	142	557	1.000076506	4.363371784	1.999754994	up
ENSG00000146085(MUT)	337	758	4.1735436	9.692563718	1.212360345	up
ENSG00000146094(DOK3)	86	234	3.734843453	8.282182958	1.463289011	up
ENSG00000146242(TPBG)	5243	1476	109.137758	31.36421513	-1.776778627	down
ENSG00000146376(ARHGAP18)	2796	1042	28.60194432	11.03243586	-1.371646309	down
ENSG00000146592(CREB5)	16	195	0.105432104	1.760500527	3.436840606	up
ENSG00000146648(EGFR)	774	4058	4.141693	20.8269709	2.43592414	up
ENSG00000146674(IGFBP3)	24	81	0.577486234	1.841875068	1.688952734	up
ENSG00000147036(LANCL3)	68	173	0.94005693	1.15198	1.359933582	up
ENSG00000147174(GCNA)	19	102	0.273415692	1.538124	2.308076296	up
ENSG00000147576(ADHFE1)	1531	508	48.08929224	16.26761004	-1.536375143	down
ENSG00000147586(MRPS28)	1032	448	133.5617493	56.90519185	-1.148933569	down
ENSG00000147689(FAM83A)	7	76	0.093903872	0.943197724	3.041347328	up
ENSG00000147813(NAPRT)	2230	848	88.171341	33.972766	-1.342037648	down
ENSG00000147883(CDKN2B)	119	354	1.613960595	4.891769091	1.599315574	up
ENSG00000147894(C9orf72)	191	418	3.595532195	7.303466869	1.167759226	up
ENSG00000148290(SURF1)	1275	525	65.05587	27.68008	-1.225715854	down
ENSG00000148339(SLC25A25)	520	1372	8.161781672	21.78321818	1.444574201	up
ENSG00000148450(MSRB2)	292	100	7.685429	2.706150675	-1.470300166	down
ENSG00000148803(FUOM)	212	58	25.01312154	7.059185172	-1.771345225	down
ENSG00000148841(ITPRIP)	466	1539	4.977278237	16.25639341	1.767218223	up
ENSG00000148925(BTBD10)	426	929	11.93949533	21.74683551	1.16933777	up
ENSG00000148926(ADM)	517	3286	21.09416569	142.236957	2.711207487	up
ENSG00000149231(CCDC82)	318	709	5.110976017	11.8882877	1.19930045	up
ENSG00000149311(ATM)	447	1024	1.944603796	5.536922278	1.2404377	up
ENSG00000149573(MPZL2)	310	946	7.41704	22.3761	1.65034766	up
ENSG00000149927(DOC2A)	279	38	9.863382147	1.471580329	-2.738215648	down
ENSG00000150051(MKX)	449	169	6.436996087	2.74115829	-1.345234664	down
ENSG00000150093(ITGB1)	7416	19964	89.1711208	245.79604	1.478301189	up
ENSG00000150593(PDCD4)	597	1319	7.709517985	17.57355857	1.189673593	up
ENSG00000150625(GPM6A)	67	16	1.406233155	0.683709465	-1.843923603	down
ENSG00000150637(CD226)	18	65	0.325010058	0.948630593	1.747275384	up
ENSG00000150779(TIMM8B)	680	274	56.63223103	23.33762	-1.252816578	down
ENSG00000150995(ITPR1)	101	249	0.892977939	1.82476955	1.326978401	up
ENSG00000151012(SLC7A11)	738	2703	4.4470009	16.39326769	1.918666533	up
ENSG00000151090(THRB)	16	70	0.250873439	1.965798486	1.994951691	up
ENSG00000151224(MAT1A)	81	22	1.229087	0.9071712	-1.707870375	down
ENSG00000151229(SLC2A13)	185	450	2.167387	4.445730116	1.318840945	up
ENSG00000151414(NEK7)	578	1655	7.79167494	23.034403	1.562868505	up
ENSG00000151474(FRMD4A)	40	133	0.555746992	2.130595968	1.712177787	up
ENSG00000151640(DPYSL4)	189	539	4.321429259	12.24035752	1.547333438	up
ENSG00000151693(ASAP2)	142	582	1.224488	5.11985	2.062785559	up
ENSG00000151694(ADAM17)	786	1919	8.336294412	20.9855	1.334509661	up
ENSG00000151967(SCHIP1)	245	557	5.875509581	16.0869982	1.225097874	up
ENSG00000152147(GEMIN6)	452	176	10.84320468	5.125859571	-1.297167378	down
ENSG00000152256(PDK1)	221	2009	2.808538587	21.224686	3.217477282	up
ENSG00000152503(TRIM36)	210	1085	2.444241672	13.10388558	2.40308576	up
ENSG00000152661(GJA1)	31	1	0.534788	0.0337252	-3.103351744	down
ENSG00000152818(UTRN)	318	677	3.48747028	5.921620141	1.132939969	up
ENSG00000152952(PLOD2)	516	6190	6.580138365	79.629721	3.627013275	up
ENSG00000153064(BANK1)	80	22	0.969629891	0.314913938	-1.69057385	down
ENSG00000153094(BCL2L11)	494	1061	5.2775304	11.65909664	1.148160901	up

ENSG00000153234(NR4A2)	122	469	1.926711865	7.200916314	1.967291752	up
ENSG00000153531(ADPRHL1)	193	796	5.86596236	7.534540303	2.077636513	up
ENSG00000153814(JAZF1)	40	135	0.732401146	3.50016246	1.733268433	up
ENSG00000153815(CMIP)	684	1455	8.968755636	19.21787309	1.135586488	up
ENSG00000154165(GPR15)	42	4	0.782506	0.239508044	-2.67104719	down
ENSG00000154217(PITPNC1)	822	1802	15.77648569	37.84355939	1.179515028	up
ENSG00000154229(PRKCA)	390	973	2.056464	5.19433	1.362378028	up
ENSG00000154263(ABCA10)	95	471	1.004325144	4.977987955	2.324753557	up
ENSG00000154553(PDLIM3)	91	336	3.039927141	11.52072651	1.901100196	up
ENSG00000154640(BTG3)	1034	2218	38.32383223	81.64948001	1.148771958	up
ENSG00000154743(TSEN2)	473	158	16.95786903	6.96972312	-1.515343929	down
ENSG00000155066(PROM2)	263	73	3.370431	0.918483652	-1.760477109	down
ENSG00000155252(PI4K2A)	896	2342	11.40338409	29.09659125	1.433198947	up
ENSG00000155304(HSPA13)	649	1811	8.59923	24.19414	1.526255069	up
ENSG00000155592(ZKSCAN2)	307	663	1.893358814	4.087693801	1.153238343	up
ENSG00000155744(FAM126B)	225	609	1.31485708	4.332668	1.474595959	up
ENSG00000156011(PSD3)	401	956	1.796002156	4.904198585	1.297187828	up
ENSG00000156273(BACH1)	389	968	3.946604859	10.14724734	1.358643236	up
ENSG00000156510(HKDC1)	6	66	0.075088856	0.909642744	2.999041653	up
ENSG00000156515(HK1)	1613	4615	21.14360951	60.894069	1.564838415	up
ENSG00000156675(RAB11FIP1)	1134	2605	8.3110472	18.542729	1.247697303	up
ENSG00000156802(ATAD2)	3074	6744	23.64551138	53.40844721	1.18269982	up
ENSG00000157315(TMED6)	41	119	0.833066	4.44868	1.521987171	up
ENSG00000157379(DHRS1)	349	85	14.8644	3.73127	-1.952751527	down
ENSG00000157456(CCNB2)	1938	843	64.5939911	28.327949	-1.148349176	down
ENSG00000157483(MYO1E)	1142	3176	12.40631953	47.8785391	1.52322905	up
ENSG00000157514(TSC22D3)	115	633	3.139070808	18.99992901	2.480882871	up
ENSG00000157625(TAB3)	309	748	3.68487755	7.412382759	1.317294834	up
ENSG00000157992(KRTCAP3)	614	218	64.47045	22.91485	-1.43226993	down
ENSG00000158055(GRHL3)	49	139	0.906634283	2.473625277	1.499851202	up
ENSG00000158079(PTPDC1)	150	462	1.424028447	4.439369	1.65393427	up
ENSG00000158483(FAM86C1)	367	101	6.585714	2.265097645	-1.783234566	down
ENSG00000158805(ZNF276)	2048	772	16.02352704	6.512532	-1.354370936	down
ENSG00000158825(CDA)	369	812	27.1613	61.5232	1.181487101	up
ENSG00000159082(SYNJ1)	118	370	1.442041732	4.224913407	1.674482685	up
ENSG00000159199(ATP5G1)	3078	1053	435.4233568	159.2138206	-1.495028121	down
ENSG00000159216(RUNX1)	379	1263	4.017510122	13.61112622	1.778732374	up
ENSG00000159256(MORC3)	512	1325	5.14172	13.63406439	1.416633074	up
ENSG00000159335(PTMS)	9026	3870	405.2419	184.8707	-1.171194184	down
ENSG00000159388(BTG2)	556	1358	11.1064	27.8534	1.33375438	up
ENSG00000159399(HK2)	915	12292	9.925699989	128.0746903	3.793528733	up
ENSG00000159733(ZFYVE28)	195	576	5.380255096	14.10976601	1.598221519	up
ENSG00000159958(TNFRSF13C)	353	117	3.665421	1.242873	-1.520772568	down
ENSG00000160193(WDR4)	451	135	17.39619029	4.685136782	-1.66973591	down
ENSG00000160221(C21orf33)	214	85	11.50469399	4.5556966	-1.254642933	down
ENSG00000160439(RDH13)	626	225	21.2883	7.663640867	-1.41503817	down
ENSG00000160446(ZDHHC12)	858	363	73.85880297	30.87221799	-1.184786677	down
ENSG00000160460(SPTBN4)	151	52	2.523038751	0.607824763	-1.439566726	down
ENSG00000160588(MPZL3)	139	621	2.312441169	9.71430129	2.186104826	up
ENSG00000160606(TLCD1)	578	153	50.590113	13.668099	-1.848538416	down
ENSG00000160688(FLAD1)	1376	469	45.24640265	16.0086839	-1.497270096	down
ENSG00000160712(IL6R)	175	1037	1.656866792	10.3613141	2.597074592	up
ENSG00000160813(PPP1R35)	608	244	51.12981	21.16683	-1.257575412	down
ENSG00000160993(ALKBH4)	431	182	11.5079	4.97	-1.18136712	down
ENSG00000161082(CELF5)	72	14	1.552911474	0.267722335	-2.105749417	down
ENSG00000161179(YDJC)	1369	524	97.29361	38.08937	-1.330851366	down
ENSG00000161509(GRIN2C)	63	287	0.82586042	3.634308	2.186510111	up
ENSG00000161547(SRSF2)	7084	2696	236.8077139	80.187905	-1.342856184	down
ENSG00000161638(ITGA5)	1257	3494	15.23186435	38.56671598	1.522695187	up
ENSG00000161956(SENP3)	1944	692	49.11145035	15.54513658	-1.436516716	down
ENSG00000161999(JMJD8)	3061	1258	101.29474	42.14893962	-1.231045174	down
ENSG00000162009(SSTR5)	47	8	3.46908	0.61446	-2.156810095	down
ENSG00000162241(SLC25A45)	32	154	0.808606867	4.239681145	2.217273066	up
ENSG00000162377(COA7)	1565	535	17.0209	5.97232	-1.493694435	down
ENSG00000162396(PARS2)	280	116	6.0591988	2.544010768	-1.201695739	down
ENSG00000162409(PRKAA2)	184	441	0.971520716	2.377099497	1.297575458	up
ENSG00000162415(ZSWIM5)	21	81	0.200594046	0.8017057	1.859477433	up
ENSG00000162433(AK4)	1279	2891	20.4460676	48.92820586	1.224651816	up
ENSG00000162490(DRAXIN)	105	499	0.663591	3.18248	2.267286166	up
ENSG00000162545(CAMK2N1)	542	1163	16.570995	36.57759	1.147219654	up
ENSG00000162591(MEGF6)	589	1559	5.840844325	13.53120105	1.449745725	up
ENSG00000162601(MYSM1)	741	1655	6.414586491	14.00837113	1.206055367	up
ENSG00000162676(GFI1)	109	272	2.362620874	5.780251707	1.34607271	up
ENSG00000162772(ATF3)	294	1746	7.474395982	47.40852857	2.608251048	up
ENSG00000162775(RBM15)	31	183	0.52718745	3.072889061	2.504010519	up
ENSG00000162998(FRZB)	33	152	0.805469498	4.023107	2.15800965	up

ENSG00000163216(SPRR2D)	1	37	0.154743263	5.692011402	3.397723325	up
ENSG00000163235(TGFA)	873	4145	11.30171876	50.40501635	2.293471364	up
ENSG00000163328(GPR155)	140	329	0.957220423	2.407161316	1.265227849	up
ENSG00000163382(NAXE)	2954	1157	169.73408	66.873357	-1.300223853	down
ENSG00000163435(ELF3)	1247	3194	26.94640202	72.671331	1.404785293	up
ENSG00000163462(TRIM46)	165	62	3.331859636	1.251079814	-1.323396011	down
ENSG00000163464(CXCR1)	0	31	0	0.695407	3.588133991	up
ENSG00000163516(ANKZF1)	519	1090	10.75761391	22.29016762	1.116146541	up
ENSG00000163521(GLB1L)	86	14	1.956354832	0.4211145	-2.352935181	down
ENSG00000163602(RYBP)	789	1989	5.151975727	13.38921652	1.380648197	up
ENSG00000163635(ATXN7)	514	1126	3.771178213	8.323142797	1.176794482	up
ENSG00000163644(PPM1K)	103	324	1.075015486	3.380166603	1.675588896	up
ENSG00000163814(CDCP1)	4078	11420	46.32643111	118.3069371	1.534931264	up
ENSG00000163872(YEATS2)	1142	2844	12.19473052	28.75770132	1.364088547	up
ENSG00000163898(LIPH)	178	533	2.156465322	6.665235153	1.616425457	up
ENSG00000163993(S100P)	832	275	9.359728698	3.342276042	-1.537539162	down
ENSG00000164011(ZNF691)	256	100	8.439010769	3.183138014	-1.282476471	down
ENSG00000164023(SGMS2)	557	1706	4.845887592	15.18218881	1.659701461	up
ENSG00000164039(BDH2)	284	100	13.45221657	4.934101742	-1.430623748	down
ENSG00000164056(SPRY1)	33	182	0.585968263	3.673936507	2.41355164	up
ENSG00000164062(APEH)	4630	1550	85.571692	29.44598736	-1.527059703	down
ENSG00000164096(C4orf3)	767	2181	19.34771	50.8708	1.554062055	up
ENSG00000164117(FBXO8)	116	263	2.6236231	6.386321	1.210503155	up
ENSG00000164142(FAM160A1)	222	579	1.482428053	2.593443284	1.421176913	up
ENSG00000164171(ITGA2)	1358	5486	7.65545	31.76562	2.061898027	up
ENSG00000164181(ELOVL7)	392	862	5.90493	13.7011734	1.180836233	up
ENSG00000164187(LMBRD2)	58	222	0.366372	1.473226	1.933928321	up
ENSG00000164211(STARD4)	1064	4575	12.93437419	58.40795603	2.151195313	up
ENSG00000164236(ANKRD33B)	453	1148	2.694258	6.895482	1.385805297	up
ENSG00000164300(SERINC5)	480	1070	3.863800378	8.881184212	1.201545164	up
ENSG00000164327(RICTOR)	411	893	2.095642021	4.65308658	1.163852601	up
ENSG00000164331(ANKRA2)	123	374	3.06508	9.46441	1.631420272	up
ENSG00000164400(CSF2)	0	26	0	2.63356	3.35726424	up
ENSG00000164411(GJB7)	0	33	0	0.872940067	3.67104308	up
ENSG00000164463(CREBRF)	102	233	0.586308	1.4483053	1.218450651	up
ENSG00000164465(DCBLD1)	508	1548	7.348458	22.442548	1.651857175	up
ENSG00000164520(RAET1E)	54	164	1.846459525	4.370921625	1.601427128	up
ENSG00000164603(BMT2)	135	492	1.759638745	6.743315267	1.893122694	up
ENSG00000164611(PTTG1)	1930	784	168.7930128	70.71745152	-1.246708561	down
ENSG00000164683(HEY1)	18	68	0.69755135	1.796769341	1.809729165	up
ENSG00000164695(CHMP4C)	295	657	7.52654	17.1226	1.197151132	up
ENSG00000164932(CTHRC1)	194	62	9.881133	4.054885288	-1.553243567	down
ENSG00000164938(TP53INP1)	344	1207	3.24731	11.90889	1.852123137	up
ENSG00000164949(GEM)	259	813	6.28944056	20.13487504	1.689138136	up
ENSG00000164951(PDP1)	20455	8483	268.5186388	118.3685234	-1.219557804	down
ENSG00000164983(TMEM65)	482	1148	2.98784	7.43491	1.296837445	up
ENSG00000165030(NFIL3)	533	1191	12.28291186	27.72046992	1.205487381	up
ENSG00000165175(MID1IP1)	2356	899	71.37378	26.49504318	-1.337246522	down
ENSG00000165195(PIGA)	166	669	2.7887206	11.27651655	2.041773553	up
ENSG00000165240(ATP7A)	192	562	1.24646436	3.62373223	1.584933733	up
ENSG00000165271(NOL6)	1448	523	21.09625279	7.71599	-1.41436467	down
ENSG00000165359(INTS6L)	106	415	1.508295297	5.209780598	1.989684999	up
ENSG00000165434(PGM2L1)	261	598	2.084728689	4.392164	1.236839825	up
ENSG00000165506(DNAAF2)	567	241	11.8754	5.09495	-1.1749932	down
ENSG00000165533(TTC8)	563	224	13.31331832	6.673713911	-1.269104148	down
ENSG00000165813(CCDC186)	699	2049	4.277333945	12.04752347	1.597515571	up
ENSG00000165819(METTL3)	1074	453	27.740754	12.2031581	-1.190412907	down
ENSG00000165861(ZFYVE1)	258	597	4.158840484	9.181833549	1.250930325	up
ENSG00000165879(FRAT1)	5	40	0.140779	1.11361	2.486126377	up
ENSG00000165891(E2F7)	432	1475	4.690803777	15.1269461	1.814648125	up
ENSG00000165898(ISCA2)	502	214	17.114734	6.867253	-1.169610461	down
ENSG00000165917(RAPSN)	1	32	0.050134428	1.278937337	3.204101722	up
ENSG00000165959(CLMN)	377	1019	2.073055	4.384887626	1.477373852	up
ENSG00000165997(ARL5B)	994	2627	6.92635	19.20632	1.449399893	up
ENSG00000166016(ABTB2)	241	1024	4.481699981	19.45329393	2.123723792	up
ENSG00000166046(TCP11L2)	59	189	0.6821812	2.7123104	1.681408476	up
ENSG00000166130(IKBIP)	468	1432	12.5239366	29.87407	1.657312732	up
ENSG00000166133(RPUSD2)	702	206	18.91506	6.342926	-1.705262982	down
ENSG00000166166(TRMT61A)	683	296	12.24409	5.342392453	-1.148808584	down
ENSG00000166189(HPS6)	1579	641	35.4486	14.6578	-1.246981432	down
ENSG00000166311(SMPD1)	73	456	1.919056	12.64432	2.645505891	up
ENSG00000166337(TAF10)	1500	627	112.749	47.45864402	-1.204790802	down
ENSG00000166343(MSS51)	32	133	0.506462665	2.013772775	2.00984725	up
ENSG00000166398(KIAA0355)	352	781	3.814279495	7.76260495	1.193027712	up
ENSG00000166401(SERPINB8)	138	674	2.5409708	10.8541623	2.313950989	up
ENSG00000166681(BEX3)	2155	803	137.77177	52.781689	-1.371144397	down

ENSG00000166833(NAV2)	43	256	0.456931438	1.543446122	2.544980839	up
ENSG00000166851(PLK1)	3658	643	102.751849	17.44072	-2.453041157	down
ENSG00000167264(DUS2)	419	166	10.61159649	4.143851112	-1.271548011	down
ENSG00000167272(POP5)	684	228	59.61844	20.28404	-1.523431043	down
ENSG00000167306(MYO5B)	850	2281	14.06704714	41.597255	1.470953694	up
ENSG00000167371(PRRT2)	889	354	29.40686424	11.96941461	-1.271760683	down
ENSG00000167562(ZNF701)	277	755	1.059283073	3.326786475	1.486849619	up
ENSG00000167619(TMEM145)	80	295	2.756304181	10.6220881	1.894748369	up
ENSG00000167700(MFSD3)	1007	143	57.5537	8.36109	-2.742075271	down
ENSG00000167767(KRT80)	259	820	3.615527	11.42534	1.701464251	up
ENSG00000167772(ANGPTL4)	120	3193	4.344310396	111.7595059	4.750478338	up
ENSG00000167964(RAB26)	1228	135	47.55431224	5.045311328	-3.108981951	down
ENSG00000167965(MLST8)	1407	592	54.92539154	24.149586	-1.19512286	down
ENSG00000167994(RAB3IL1)	322	127	7.596734888	2.981692249	-1.273648582	down
ENSG00000168140(VASN)	204	637	5.2445	16.2127	1.678616179	up
ENSG00000168152(THAP9)	101	243	1.3352	3.2978	1.292186632	up
ENSG00000168209(DDIT4)	1776	5529	61.6442	194.99	1.686734501	up
ENSG00000168214(RBPJ)	1384	3335	13.30077299	31.83548509	1.317004205	up
ENSG00000168569(TMEM223)	478	194	26.65825	11.122561	-1.238978016	down
ENSG00000168672(FAM84B)	532	1193	7.01093877	18.17302	1.210596874	up
ENSG00000168743(NPNT)	230	542	4.045027	8.51861	1.275957587	up
ENSG00000168769(TET2)	223	480	1.762855116	3.408736769	1.145684795	up
ENSG00000169031(COL4A3)	71	260	0.815564575	2.390528108	1.88015269	up
ENSG00000169032(MAP2K1)	1317	2800	24.9101229	52.92502116	1.13641102	up
ENSG00000169047(IRS1)	1910	447	10.6818451	2.550840137	-2.038400691	down
ENSG00000169126(ARMC4)	197	70	2.319690667	0.87016	-1.407141098	down
ENSG00000169155(ZBTB43)	285	770	2.336350513	6.593637006	1.474472357	up
ENSG00000169403(PTAFR)	145	586	1.9317	7.4646266	2.043057396	up
ENSG00000169429(CXCL8)	7	150	0.212726	4.846868	3.996622984	up
ENSG00000169499(PLEKHA2)	404	1009	4.426753	9.920561431	1.3641334	up
ENSG00000169826(CSGALNACT2)	332	999	6.034935	16.544504	1.630739813	up
ENSG00000169851(PCDH7)	3080	1154	30.60988253	11.30506742	-1.364162832	down
ENSG00000169884(WNT10B)	252	91	7.02825202	2.593855856	-1.392178845	down
ENSG00000169967(MAP3K2)	691	1857	2.8793	8.03465	1.472311263	up
ENSG00000169972(PUSL1)	769	332	51.0326	22.24635	-1.155108374	down
ENSG00000170035(UBE2E3)	1393	483	77.27142565	27.34843993	-1.472761657	down
ENSG00000170113(NIPA1)	800	1755	6.745688169	14.232564	1.18044565	up
ENSG00000170175(CHRNB1)	280	628	7.46996813	15.19971607	1.206839407	up
ENSG00000170231(FABP6)	97	19	7.542957938	1.483009	-2.145575757	down
ENSG00000170345(FOS)	242	1092	6.773720309	30.94987315	2.210332171	up
ENSG00000170379(TCAF2)	32	282	0.260078212	2.5169291	3.078249381	up
ENSG00000170396(ZNF804A)	67	229	0.63498967	2.222831	1.779346521	up
ENSG00000170456(DENND5B)	1002	399	6.441524094	2.434284001	-1.272500069	down
ENSG00000170468(RIOX1)	126	16	3.57655	0.45572	-2.726712043	down
ENSG00000170525(PFKFB3)	1051	2232	13.2454786	28.19909687	1.134377183	up
ENSG00000170734(POLH)	672	1950	4.716970266	13.126704	1.582760953	up
ENSG00000170836(PPM1D)	963	3039	11.3896431	37.8284741	1.704945728	up
ENSG00000170871(KIAA0232)	452	968	2.532417208	5.9085256	1.143584743	up
ENSG00000170906(NDUFA3)	509	220	117.3333769	48.355243	-1.150091951	down
ENSG00000170917(NUDT6)	128	26	6.965500008	1.901902	-2.133891389	down
ENSG00000170949(ZNF160)	278	1033	3.04529817	11.362839	1.932555252	up
ENSG00000171124(FUT3)	18	100	1.153675048	3.406080085	2.34767566	up
ENSG00000171132(PRKCE)	100	364	1.0253923	3.951161	1.883624473	up
ENSG00000171310(CHST11)	270	714	4.636072978	18.30274821	1.443142062	up
ENSG00000171346(KRT15)	245	991	5.3662929	18.25371	2.053129063	up
ENSG00000171425(ZNF581)	1318	577	94.28647472	37.04268443	-1.137900592	down
ENSG00000171444(MCC)	272	658	2.2358266	4.650733738	1.315252954	up
ENSG00000171462(DLK2)	17	76	1.1483657	3.524000057	2.034849794	up
ENSG00000171522(PTGER4)	30	141	0.46666303	2.74627	2.177533691	up
ENSG00000171681(ATF7IP)	788	192	4.732463104	1.357456836	-1.971459686	down
ENSG00000171903(CYP4F11)	460	1011	4.697096401	9.326845308	1.180960527	up
ENSG00000172007(RAB33B)	202	554	2.4782	7.04929	1.492171185	up
ENSG00000172071(EIF2AK3)	237	670	4.586030945	9.873652771	1.537682215	up
ENSG00000172183(ISG20)	74	443	3.87427774	27.19957902	2.585157054	up
ENSG00000172269(DPAGT1)	474	183	15.5889594	8.131685323	-1.309902895	down
ENSG00000172590(MRPL52)	937	354	113.649487	42.45814561	-1.347385119	down
ENSG00000172613(RAD9A)	476	205	14.0543682	5.95416	-1.15450466	down
ENSG00000172638(EFEMP2)	10	72	0.512723179	2.527872219	2.584687038	up
ENSG00000172731(LRRC20)	664	290	12.17264883	5.935848196	-1.137544872	down
ENSG00000172794(RAB37)	207	56	4.32974702	1.205815856	-1.785629536	down
ENSG00000172869(DMXL1)	306	744	3.16192427	4.4167245	1.323531438	up
ENSG00000172888(ZNF621)	638	1424	5.89239814	10.8938867	1.204576277	up
ENSG00000172901(LVRN)	248	600	5.424637826	17.62250065	1.314510236	up
ENSG00000172939(OXSR1)	795	1980	8.13412	20.6407707	1.363225066	up
ENSG00000172992(DCAKD)	538	230	18.179448	8.0953	-1.166251605	down
ENSG00000173166(RAPH1)	315	1256	2.635819522	8.200228336	2.035345953	up

ENSG00000173221(GLRX)	421	1333	26.910404	77.96493974	1.705861133	up
ENSG00000173281(PPP1R3B)	224	599	1.956122	5.408406	1.457164429	up
ENSG00000173517(PEAK1)	414	886	1.5270148	4.083320984	1.142116037	up
ENSG00000173530(TNFRSF10D)	803	4139	13.41541	67.4855	2.411546758	up
ENSG00000173548(SNX33)	346	886	3.43723	8.36373	1.39899671	up
ENSG00000173575(CHD2)	2274	4892	45.41565173	49.67304459	1.154152154	up
ENSG00000173706(HEG1)	83	214	0.650081965	1.474388384	1.385510432	up
ENSG00000173926(MARCH3)	46	124	0.7518412	3.336909247	1.424444293	up
ENSG00000174010(KLHL15)	350	857	2.9683765	7.5084381	1.334694291	up
ENSG00000174177(CTU2)	1603	427	58.138198	16.054256	-1.851639283	down
ENSG00000174276(ZNHIT2)	427	85	32.074327	6.353850041	-2.241579291	down
ENSG00000174373(RALGAPA1)	419	925	5.196101506	8.452753509	1.186871003	up
ENSG00000174460(ZCCHC12)	81	22	1.87414	0.534248	-1.707870375	down
ENSG00000174669(SLC29A2)	1563	565	29.64829422	8.799829561	-1.41354151	down
ENSG00000174799(CEP135)	308	823	3.688142228	8.942613	1.459289387	up
ENSG00000174950(CD164L2)	210	76	8.6737	2.430343	-1.383794004	down
ENSG00000175084(DES)	88	18	2.238673392	0.49396	-2.077094681	down
ENSG00000175105(ZNF654)	147	976	1.126843	7.8895	2.756905705	up
ENSG00000175197(DDIT3)	226	908	12.6872	51.616109	2.042345047	up
ENSG00000175265(GOLGA8A)	1087	3882	11.02651914	40.63631	1.883611336	up
ENSG00000175305(CCNE2)	591	2377	10.56765	47.093381	2.052506222	up
ENSG00000175567(UCP2)	424	146	16.313733	5.62706133	-1.47047191	down
ENSG00000175602(CCDC85B)	2879	1251	166.688	72.1342	-1.150728684	down
ENSG00000175792(RUVBL1)	3609	1448	97.4284168	39.74697797	-1.265925664	down
ENSG00000175793(SFN)	3167	8876	150.753	422.166	1.535900915	up
ENSG00000175984(DENND2C)	169	441	1.2694416	3.418494	1.418265391	up
ENSG00000176018(LYSMD3)	546	1245	6.359463031	15.090777	1.234733486	up
ENSG00000176125(UFSP1)	59	13	5.28013	1.18346	-1.919240279	down
ENSG00000176171(BNIP3)	798	4719	15.96398754	102.5055464	2.60960391	up
ENSG00000176410(DNAJC30)	287	114	6.63421	2.727793	-1.261449784	down
ENSG00000176438(SYNE3)	51	428	0.344981627	2.099741437	3.04839402	up
ENSG00000176641(RNF152)	37	141	0.320270664	0.820827935	1.899313511	up
ENSG00000176697(BDNF)	1736	566	19.52855679	6.577138541	-1.562184865	down
ENSG00000176714(CCDC121)	76	12	1.402146	0.2221056	-2.363530204	down
ENSG00000176783(RUFY1)	1178	438	24.68827766	8.48527562	-1.371689646	down
ENSG00000177076(ACER2)	88	404	0.898171941	4.24393047	2.211817388	up
ENSG00000177119(ANO6)	1120	2560	9.412638148	21.66404317	1.240460823	up
ENSG00000177125(ZBTB34)	201	457	2.015384973	4.415626304	1.223082534	up
ENSG00000177150(FAM210A)	597	1645	8.851730003	33.908624	1.507708371	up
ENSG00000177181(RIMKLA)	176	846	1.66155105	6.028776811	2.296182262	up
ENSG00000177192(PUS1)	1696	685	74.199383	27.4288003	-1.254548403	down
ENSG00000177352(CCDC71)	893	300	27.2675	9.21623	-1.514958518	down
ENSG00000177606(JUN)	161	2200	2.79051	38.5716	3.798342033	up
ENSG00000177854(TMEM187)	197	62	10.884889	2.95302624	-1.57505777	down
ENSG00000177943(MAMDC4)	60	162	1.048524775	2.70701	1.439419379	up
ENSG00000178038(ALS2CL)	290	631	2.781405529	6.19897863	1.163570099	up
ENSG00000178057(NDUFAF3)	1319	371	69.50593	17.6679	-1.772265465	down
ENSG00000178105(DDX10)	1882	668	42.5020286	13.8528326	-1.44054039	down
ENSG00000178385(PLEKHM3)	94	271	0.559099999	1.739740077	1.548433824	up
ENSG00000178531(CTXN1)	1560	594	119.418	45.4986	-1.338911642	down
ENSG00000178752(ERFE)	262	567	6.73696268	15.216034	1.154955852	up
ENSG00000178896(EXOSC4)	1731	609	148.2847723	53.21710887	-1.452894013	down
ENSG00000178917(ZNF852)	221	480	1.270421122	2.88145051	1.158513937	up
ENSG00000179104(TMTC2)	214	669	2.977664	7.9169965	1.680932731	up
ENSG00000179148(ALOXE3)	106	286	2.2013438	6.901642	1.456960881	up
ENSG00000179361(ARID3B)	243	831	2.92995163	10.152987	1.811564116	up
ENSG00000179388(EGR3)	91	481	1.331028906	7.114653811	2.415062501	up
ENSG00000179403(VWA1)	1547	676	48.37767309	19.20449746	-1.141125696	down
ENSG00000179431(FJX1)	512	132	17.0132	4.43272	-1.883402455	down
ENSG00000179454(KLHL28)	193	600	1.64314393	5.283101	1.671488311	up
ENSG00000179526(SHARPIN)	663	266	27.906088	11.392091	-1.258757384	down
ENSG00000179546(HTR1D)	0	31	7.46E-05	0.64011	3.588133991	up
ENSG00000179630(LACC1)	51	165	0.59415318	1.834158699	1.688178488	up
ENSG00000179833(SERTAD2)	1391	3746	12.88041	35.64169	1.47727177	up
ENSG00000179886(TIGD5)	635	275	7.1314	3.14044	-1.14927165	down
ENSG00000179958(DCTPP1)	3357	1214	186.6228479	71.19782533	-1.41534073	down
ENSG00000180008(SOCS4)	822	1781	6.101235	13.59266397	1.162630032	up
ENSG00000180340(FZD2)	520	118	16.19439	4.824098	-2.063830667	down
ENSG00000180537(RNF182)	93	321	1.390240193	4.80306583	1.805369278	up
ENSG00000180667(YOD1)	501	1478	4.114089299	12.61605393	1.605124904	up
ENSG00000180998(GPR137C)	28	94	0.412900436	1.325267059	1.697055521	up
ENSG00000181085(MAPK15)	472	186	9.295798002	3.144724506	-1.28073315	down
ENSG00000181218(HIST3H2A)	216	68	21.1138	6.6537	-1.578310676	down
ENSG00000181450(ZNF678)	116	285	0.971079801	2.207658879	1.3252239	up
ENSG00000181458(TMEM45A)	129	658	4.633326037	24.42722912	2.374655389	up
ENSG00000181544(FANCB)	110	270	1.453764	3.6876524	1.322700543	up

ENSG00000181652(ATG9B)	47	180	0.644107001	2.189270397	1.922916648	up
ENSG00000181690(PLAG1)	10	48	0.076075968	0.343974229	2.026581416	up
ENSG00000181744(C3orf58)	369	1006	5.703632455	15.32567766	1.489605504	up
ENSG00000181817(LSM10)	758	316	62.8859	27.006	-1.20504699	down
ENSG00000181894(ZNF329)	38	108	0.508958454	1.437805515	1.487539083	up
ENSG00000182022(CHST15)	983	2529	17.65650433	42.6651103	1.410617797	up
ENSG00000182389(CACNB4)	38	141	0.246224481	0.94431717	1.86361072	up
ENSG00000182580(EPHB3)	180	533	2.52911	7.45775	1.600563708	up
ENSG00000182606(TRAK1)	814	1770	10.13743375	19.12729518	1.16776558	up
ENSG00000182759(MAFA)	43	127	1.62117	4.71629	1.549496937	up
ENSG00000182795(C1orf116)	29	186	0.258225824	1.72415429	2.614818135	up
ENSG00000182798(MAGEB17)	5	37	0.217330661	1.5106681	2.38131305	up
ENSG00000182810(DDX28)	362	120	10.7121	3.56346	-1.521121444	down
ENSG00000183011(NAA38)	1213	530	152.2072	57.797624	-1.140363658	down
ENSG00000183018(SPNS2)	69	295	1.305410019	6.170348	2.100100669	up
ENSG00000183048(SLC25A10)	8686	2852	475.1561611	153.5775856	-1.555803365	down
ENSG00000183161(FANCF)	145	50	1.977492	0.704094	-1.435826116	down
ENSG00000183421(RIPK4)	359	815	5.292301075	12.08332309	1.226111621	up
ENSG00000183508(FAM46C)	74	286	0.64605	2.55145	1.958851291	up
ENSG00000183696(UPP1)	392	1839	17.11978922	79.62032295	2.271504097	up
ENSG00000183760(ACP7)	73	199	1.263196967	3.994959456	1.460612454	up
ENSG00000183779(ZNF703)	162	431	4.31081	11.3927	1.445361028	up
ENSG00000183978(COA3)	520	175	52.1475	17.8548	-1.506239564	down
ENSG00000184178(SCFD2)	294	96	4.735415741	2.340586	-1.537301913	down
ENSG00000184502(GAST)	6	39	2.0035	13.4427	2.280290843	up
ENSG00000184545(DUSP8)	177	523	2.78700977	8.332425146	1.597242357	up
ENSG00000184557(SOCS3)	476	1028	10.02541196	22.53604276	1.155928855	up
ENSG00000184731(FAM110C)	357	1614	4.348978	19.461322	2.217417686	up
ENSG00000184897(H1FX)	3001	1111	183.81	66.3023	-1.381360364	down
ENSG00000184900(SUMO3)	626	178	18.82749236	5.579682673	-1.748392419	down
ENSG00000184967(NOC4L)	1073	388	47.51315239	17.21005723	-1.411037062	down
ENSG00000185015(CA13)	151	391	2.2740167	6.078767	1.405389531	up
ENSG00000185022(MAFF)	421	1555	10.50161777	38.515663	1.927665377	up
ENSG00000185033(SEMA4B)	1026	2314	17.67935783	37.77617972	1.220999564	up
ENSG00000185201(IFITM2)	995	425	142.19499	61.9428586	-1.171958964	down
ENSG00000185361(TNFAIP8L1)	985	337	12.8935009	4.483104794	-1.489669527	down
ENSG00000185507(IRF7)	117	399	4.447450703	15.32187632	1.794536735	up
ENSG00000185522(LMNTD2)	42	172	1.4385987	5.484475001	2.010650369	up
ENSG00000185551(NR2F2)	517	1194	8.796835399	19.27000551	1.252837056	up
ENSG00000185567(AHNAK2)	1107	3257	6.563470153	17.06129717	1.604321601	up
ENSG00000185630(PBX1)	1425	241	14.89572546	2.522834239	-2.500091432	down
ENSG00000185697(MYBL1)	306	1168	3.3662326	12.731289	1.972224259	up
ENSG00000185745(IFIT1)	132	31	3.0870759	0.64069143	-1.946443449	down
ENSG00000185950(IRS2)	225	1024	2.285895013	10.0510933	2.221608253	up
ENSG00000186205(MARC1)	662	273	20.41893605	8.950785236	-1.219501494	down
ENSG00000186352(ANKRD37)	18	1031	1.24878	73.38808	5.677576121	up
ENSG00000186470(BTN3A2)	560	159	7.00789506	1.85047031	-1.748615622	down
ENSG00000186523(FAM86B1)	229	83	5.771290001	2.16084291	-1.384350084	down
ENSG00000186529(CYP4F3)	43	296	0.44130239	3.06020117	2.752314838	up
ENSG00000186765(FSCN2)	125	886	1.364787	6.247642	2.846718815	up
ENSG00000186832(KRT16)	16	127	0.537104418	4.18489098	2.829132242	up
ENSG00000186847(KRT14)	4	37	0.133878	1.21943	2.576291055	up
ENSG00000186854(TRABD2A)	73	18	0.91443415	0.250991492	-1.816947295	down
ENSG00000186891(TNFRSF18)	586	225	39.28257	14.413363	-1.320231848	down
ENSG00000187024(PTRH1)	363	142	35.4079607	14.3325602	-1.287338958	down
ENSG00000187193(MT1X)	589	1441	257.3290001	655.5481	1.33640778	up
ENSG00000187514(PTMA)	81584	29661	3187.391681	1209.967186	-1.409657259	down
ENSG00000187634(SAMD11)	716	305	21.62751137	9.623490893	-1.173788946	down
ENSG00000187713(TMEM203)	1122	484	50.2889	22.0727	-1.158398833	down
ENSG00000187867(PALM3)	373	116	7.161211	2.227512	-1.611739193	down
ENSG00000187961(KLHL17)	1122	465	32.08741744	13.28726	-1.215834764	down
ENSG00000188064(WNT7B)	190	816	2.978142552	13.49934493	2.135575827	up
ENSG00000188130(MAPK12)	1297	570	45.92291478	20.11972299	-1.132303664	down
ENSG00000188186(LAMTOR4)	1329	547	202.751172	90.926108	-1.22651189	down
ENSG00000188321(ZNF559)	190	415	4.094815067	9.845090107	1.16489655	up
ENSG00000188338(SLC38A3)	70	249	1.371683908	4.861733808	1.83812117	up
ENSG00000188343(FAM92A)	2629	946	77.87640619	34.73044804	-1.421940674	down
ENSG00000188372(ZP3)	147	23	5.91562	0.936376431	-2.48804315	down
ENSG00000188483(IER5L)	6118	1386	195.129	44.1618	-2.089919078	down
ENSG00000188486(H2AFX)	4130	1189	245.473	70.0939	-1.744025667	down
ENSG00000188559(RALGAPA2)	241	722	1.421546732	3.772945112	1.621230144	up
ENSG00000188610(FAM72B)	160	38	3.370503194	0.872405673	-1.947058137	down
ENSG00000188868(ZNF563)	51	166	0.710164598	2.008580416	1.696750094	up
ENSG00000188910(GJB3)	789	2081	23.68410336	61.34924188	1.445791857	up
ENSG00000188958(UTS2B)	57	11	1.732806	0.255332701	-2.066970189	down
ENSG00000188993(LRRC66)	7	49	0.101499	0.688165	2.436212873	up

ENSG00000188994(ZNF292)	902	2477	5.8429092	16.3722102	1.504359832	up
ENSG00000189190(ZNF600)	151	413	1.726108291	4.6481948	1.483813962	up
ENSG00000189308(LIN54)	487	1153	4.498025387	11.13038415	1.288292314	up
ENSG00000196083(IL1RAP)	35	436	0.585363076	5.561805874	3.582907069	up
ENSG00000196110(ZNF699)	54	326	0.492538156	3.049701168	2.580469423	up
ENSG00000196230(TUBB)	37550	8229	791.230482	173.864228	-2.139666414	down
ENSG00000196247(ZNF107)	272	724	2.224360058	6.43482822	1.45259542	up
ENSG00000196352(CD55)	629	3279	11.44655937	63.85489663	2.426676609	up
ENSG00000196497(IPO4)	2423	868	40.7129542	13.818821	-1.428117617	down
ENSG00000196502(SULT1A1)	555	188	21.65182086	7.04994806	-1.497909696	down
ENSG00000196639(HRH1)	213	511	2.784764866	5.976863154	1.300781861	up
ENSG00000196646(ZNF136)	16	79	0.194013583	0.952073	2.163089161	up
ENSG00000196712(NF1)	353	827	4.001198413	5.57148666	1.271245391	up
ENSG00000196878(LAMB3)	685	5925	8.888937157	79.2148862	3.157183185	up
ENSG00000196968(FUT11)	476	3219	5.168504462	30.72848214	2.80002047	up
ENSG00000197044(ZNF441)	11	70	0.10285815	0.643271	2.43796411	up
ENSG00000197061(HIST1H4C)	792	191	294.58	77.5517	-1.986162343	down
ENSG00000197321(SVIL)	335	951	2.0582271	5.87763401	1.547039241	up
ENSG00000197329(PELI1)	139	383	2.068025408	5.510502832	1.492890727	up
ENSG00000197343(ZNF655)	802	1684	16.41459386	26.053348	1.117374845	up
ENSG00000197448(GSTK1)	1276	476	77.31712254	29.13053135	-1.367409362	down
ENSG00000197461(PDGFA)	281	1050	7.36970849	28.45544749	1.940715272	up
ENSG00000197467(COL13A1)	65	244	1.187661191	4.13719897	1.911678506	up
ENSG00000197535(MYO5A)	607	1435	3.027004331	7.211461995	1.287181425	up
ENSG00000197603(C5orf42)	228	489	1.170426534	2.679955164	1.140747782	up
ENSG00000197747(S100A10)	2126	5398	148.001927	378.10599	1.393037332	up
ENSG00000197822(OCLN)	646	1480	6.399472248	17.371942	1.242220136	up
ENSG00000197852(FAM212B)-2	145	444	2.423199875	4.188994091	1.644917813	up
ENSG00000197915(HRNR)	0	32	0	0.189398	3.630184033	up
ENSG00000197930(ERO1A)	2291	7951	23.3895438	103.4482201	1.843813711	up
ENSG00000197969(VPS13A)	914	2155	8.251891972	17.72392199	1.284692399	up
ENSG00000197978(GOLGA6L9)	160	382	2.636931539	6.941600502	1.290048279	up
ENSG00000198130(HIBCH)	572	226	15.28797	7.238477	-1.279201845	down
ENSG00000198142(SOWAHC)	699	2233	8.05559	26.5088	1.721415939	up
ENSG00000198157(HMGN5)	818	347	17.70790032	6.415037466	-1.18067108	down
ENSG00000198265(HELZ)	743	1615	3.47455645	7.73641704	1.166945437	up
ENSG00000198393(ZNF26)	119	355	1.027425022	1.393322007	1.603353217	up
ENSG00000198538(ZNF28)	344	853	3.580292529	9.249388001	1.352705794	up
ENSG00000198574(SH2D1B)	0	26	0	0.576484	3.35726424	up
ENSG00000198576(ARC)	590	1253	13.1425	27.3675	1.132710001	up
ENSG00000198695(MT-ND6)	23585	9431	5007.35	2262.37	-1.272158484	down
ENSG00000198712(MT-CO2)	125131	47565	22996.1	8769.21	-1.345438776	down
ENSG00000198715(GLMP)	613	192	31.40468023	10.94093804	-1.610665977	down
ENSG00000198729(PPP1R14C)	136	465	3.335477	11.88303	1.80174763	up
ENSG00000198783(ZNF830)	56	0	1.18387	2.49E-30	-4.339171914	down
ENSG00000198794(SCAMP5)	367	153	6.818273897	2.941662823	-1.197398804	down
ENSG00000198840(MT-ND3)	19069	5931	25597.1	8699.73	-1.634441913	down
ENSG00000198862(LTN1)	222	538	1.95140222	4.800348812	1.315750917	up
ENSG00000198873(GRK5)	136	427	2.75455	8.25088	1.679524368	up
ENSG00000198887(SMC5)	946	2068	6.84427	15.52675	1.175828691	up
ENSG00000198964(SGMS1)	279	855	4.994016833	14.49172618	1.655399652	up
ENSG00000203326(ZNF525)	137	308	1.743403256	3.454269758	1.2015115	up
ENSG00000203943(SAMD13)	140	32	5.281544128	1.246125148	-1.987502671	down
ENSG00000204103(MAFB)	6	46	0.119926	0.891861	2.503612464	up
ENSG00000204186(ZDBF2)	159	461	0.708068311	2.143175524	1.568318042	up
ENSG00000204220(PFDN6)	1657	577	222.0688173	79.42676291	-1.467473323	down
ENSG00000204228(HSD17B8)	226	51	14.8022	3.47973	-2.038822744	down
ENSG00000204237(OXLD1)	364	86	56.68548	13.7002	-1.996634531	down
ENSG00000204316(MRPL38)	2693	1098	119.4804	49.6643	-1.242228786	down
ENSG00000204371(EHMT2)	2646	1078	33.9720025	13.85589339	-1.243307802	down
ENSG00000204386(NEU1)	472	1081	13.689	31.6086	1.240362595	up
ENSG00000204388(HSPA1B)	2304	966	53.7137783	22.19141636	-1.201697784	down
ENSG00000204394(VARS)	7130	2790	152.7460365	56.18633252	-1.302796236	down
ENSG00000204576(PRR3)	306	116	11.21617	4.062439	-1.328538914	down
ENSG00000204822(MRPL53)	732	308	51.82537	22.12706	-1.191552579	down
ENSG00000204922(UQCC3)	843	205	26.97124	6.43937	-1.975253584	down
ENSG00000205085(FAM71F2)	1	99	0.0111086	1.409101152	4.752368216	up
ENSG00000205129(C4orf47)	23	102	1.072147	5.049115	2.066162148	up
ENSG00000205413(SAMD9)	254	682	1.454512	3.973933559	1.464417239	up
ENSG00000205544(TMEM256)	650	214	224.10422	78.33101	-1.540468005	down
ENSG00000205923(CEMP1)	281	104	17.38101	5.82546	-1.360393814	down
ENSG00000206075(SERPINB5)	708	2047	12.0388042	35.55547484	1.577727764	up
ENSG00000206538(VGLL3)	0	46	0	0.236243689	4.117841947	up
ENSG00000206561(COLQ)	59	13	1.603367025	0.368738521	-1.919240279	down
ENSG00000212643(ZRSR1)	51	153	0.869677	2.57301	1.581138576	up
ENSG00000212724(KRTAP2-3)	105	273	13.1445	34.0954	1.403823084	up

ENSG00000213020(ZNF611)	100	257	0.761174741	1.639105645	1.386050783	up
ENSG00000213123(TCTEX1D2)	124	33	15.7126	4.47375	-1.775343179	down
ENSG00000213199(ASIC3)	23	87	0.922507972	3.367597955	1.843337066	up
ENSG00000213339(QTRT1)	816	323	43.00531814	16.23687109	-1.279692138	down
ENSG00000213465(ARL2)	1002	423	73.755995	32.44785	-1.188803997	down
ENSG00000213859(KCTD11)	135	1087	2.68166	21.8503	3.032250486	up
ENSG00000214026(MRPL23)	520	211	66.7453	27.23263611	-1.240248654	down
ENSG00000214193(SH3D21)	14	96	0.297881675	1.831095	2.596732595	up
ENSG00000214435(AS3MT)	24	141	0.3800082	1.527562	2.46791215	up
ENSG00000214736(TOMM6)	3658	1585	599.476	271.8117	-1.155186453	down
ENSG00000221843(C2orf16)	10	56	0.0498133	0.1481733	2.237524911	up
ENSG00000222009(BTBD19)	105	324	2.056230664	4.371965989	1.648599279	up
ENSG00000223572(CKMT1A)	154	54	4.74647769	1.83788588	-1.415712518	down
ENSG00000225830(ERCC6)	314	731	2.222768164	4.106149538	1.261310259	up
ENSG00000225932(CTAGE4)	12	85	0.198192	1.38135	2.608053746	up
ENSG00000227057(WDR46)	2059	720	43.50442	15.33370005	-1.462314029	down
ENSG00000232653(GOLGA8N)	125	339	1.11907673	3.068552954	1.468042092	up
ENSG00000234719(NPIPB2)	73	191	3.912038795	15.3664021	1.402258223	up
ENSG00000235109(ZSCAN31)	187	36	4.707922971	0.703188407	-2.240789849	down
ENSG00000235173(HGH1)	1334	539	26.46732	6.35859	-1.253063705	down
ENSG00000235750(KIAA0040)	455	1928	4.797139685	20.09318165	2.126046103	up
ENSG00000237330(RNF223)	44	236	1.59234	8.52068	2.397864681	up
ENSG00000239900(ADSL)	3090	1212	100.7120839	41.675409	-1.298256236	down
ENSG00000240065(PSMB9)	213	62	20.3634	6.29396	-1.68613691	down
ENSG00000242247(ARFGAP3)	409	878	7.31622704	15.7595804	1.146478662	up
ENSG00000242265(PEG10)	384	125	2.856232749	0.944264904	-1.548006633	down
ENSG00000242485(MRPL20)	1658	556	179.4401991	59.89960458	-1.521564445	down
ENSG00000243566(UPK3B)	62	13	3.758267279	0.642469852	-1.987508005	down
ENSG00000244187(TMEM141)	843	340	78.4554	32.0763	-1.253113385	down
ENSG00000244219(GS1-259H13.2)	35	3	2.559113	0.291828814	-2.655656492	down
ENSG00000244693(CTAGE8)	34	101	0.543567	1.63485	1.541837458	up
ENSG00000247315(ZCCHC3)	160	37	2.99674	0.699606	-1.982848705	down
ENSG00000249992(TMEM158)	375	1528	23.5718	95.4597	2.068156432	up
ENSG00000250479(CHCHD10)	449	164	228.42516	85.85923	-1.387842534	down
ENSG00000251192(ZNF674)	71	229	1.092862794	2.691478127	1.699076374	up
ENSG00000251493(FOXD1)	353	1271	15.7499	56.2423	1.889541678	up
ENSG00000253276(CCDC71L)	449	173	13.60163	4.97684	-1.312031814	down
ENSG00000254726(MEX3A)	439	176	3.571645	1.463605	-1.255338857	down
ENSG00000256269(HMBS)	1176	399	41.21048536	14.91011777	-1.502887242	down
ENSG00000261150(EPPK1)	526	1213	2.16523222	4.901890306	1.250800361	up
ENSG00000261236(BOP1)	6477	2690	176.111845	72.64845549	-1.216884213	down
ENSG00000261609(GAN)	879	2111	4.42475	9.54583	1.311118951	up
ENSG00000261949(GFY)	116	32	4.142004	1.137221918	-1.722266926	down
ENSG00000266074(BAHCC1)-3	47	6	1.5351	0.219563	-2.45191931	down
ENSG00000266094(RASSF5)	131	411	1.8264942	5.733454518	1.677680569	up
ENSG00000267855(NDUFA7)	697	189	126.065	34.8364	-1.81750533	down
ENSG00000268089(GABRQ)	205	451	1.717071	3.87166	1.176106601	up
ENSG00000271079(CTAGE15)	21	105	0.346667	1.70308	2.222768649	up
ENSG00000272398(CD24)	424	174	10.82696342	4.13721407	-1.221745714	down
ENSG00000273748(AL592183.1)	92	221	4.18281605	12.377144	1.287620146	up
ENSG00000275342(PRAG1)	538	1504	9.26574	25.30519	1.528013471	up
ENSG00000277972(CISD3)	983	299	23.88553	7.37454	-1.657832802	down
ENSG00000278535(DHRS11)	317	127	13.31097006	5.828821	-1.251274561	down
ENSG00000278619(MRM1)	216	81	7.277304311	2.769175922	-1.335145926	down
ENSG00000278817(AC007325.4)	13	54	0.733236	2.98839	1.886175885	up
ENSG00000283199(ABC13-4748860	10	74	0.120246184	1.013683	2.622749337	up
Homo_sapiens_newGene_10034	69	179	1.31374	3.27599	1.388144451	up
Homo_sapiens_newGene_10169	4	131	0.0692502	2.00594	4.325237757	up
Homo_sapiens_newGene_1021	568	1917	3.910048	12.9348	1.799604511	up
Homo_sapiens_newGene_10308	7	47	0.18514696	1.402339	2.379419301	up
Homo_sapiens_newGene_10318	2	48	0.068858191	1.371698	3.421179061	up
Homo_sapiens_newGene_10430	3	44	0.0435646	0.649129	3.03547806	up
Homo_sapiens_newGene_10527	260	612	1.661925688	3.535984507	1.275548542	up
Homo_sapiens_newGene_1065	43	130	0.618607283	2.385694	1.58245883	up
Homo_sapiens_newGene_10716	0	25	1.48E-06	0.349867	3.306300903	up
Homo_sapiens_newGene_1080	15	61	0.58509034	2.111634128	1.882983388	up
Homo_sapiens_newGene_10881	5	158	0.0324114	0.907567	4.395428346	up
Homo_sapiens_newGene_11115	19	68	1.471243791	5.049162	1.742334411	up
Homo_sapiens_newGene_11116	149	457	2.982981232	7.304748	1.647796936	up
Homo_sapiens_newGene_11138	2	83	0.018571	0.755927	4.177195795	up
Homo_sapiens_newGene_11228	0	32	0	0.927953	3.630184033	up
Homo_sapiens_newGene_11270	87	384	1.274194191	4.914809	2.15504492	up
Homo_sapiens_newGene_11382	160	57	22.46045	6.98399	-1.395610213	down
Homo_sapiens_newGene_11606	19	85	0.2033026	0.90242583	2.052760892	up
Homo_sapiens_newGene_11636	31	177	0.512549	2.9795	2.456656	up
Homo_sapiens_newGene_11637	7	56	0.288264	2.08049	2.619039033	up

Homo_sapiens_newGene_11656	1	65	0.0135073	0.718801	4.166069068	up
Homo_sapiens_newGene_1166	109	326	2.24451	6.76714	1.604891148	up
Homo_sapiens_newGene_11670	50	3	5.76039831	0.402315604	-3.13661274	down
Homo_sapiens_newGene_12009	0	41	8.17E-07	0.585761	3.96193024	up
Homo_sapiens_newGene_12075	0	44	1.03E-06	0.482852	4.057483768	up
Homo_sapiens_newGene_12367	63	13	1.02381	0.227056	-2.009564441	down
Homo_sapiens_newGene_1283	30	110	0.707189877	2.850165449	1.827268601	up
Homo_sapiens_newGene_1308	1	29	0.005989839	0.168437306	3.074085878	up
Homo_sapiens_newGene_1363	53	8	3.389005	0.630440129	-2.320585893	down
Homo_sapiens_newGene_1424	1264	316	7.131078	1.858746888	-1.940562901	down
Homo_sapiens_newGene_1688	210	0	0.474374	3.40E-06	-6.192821787	down
Homo_sapiens_newGene_1862	36	130	0.871854275	3.1424178	1.821123287	up
Homo_sapiens_newGene_1994	257	35	3.369541118	0.474521469	-2.731144209	down
Homo_sapiens_newGene_2238	11	66	0.12589679	1.028777	2.356447394	up
Homo_sapiens_newGene_2250	34	146	0.85492799	3.641111986	2.061357493	up
Homo_sapiens_newGene_2327	3	41	0.076468227	1.214102	2.939924532	up
Homo_sapiens_newGene_2356	715	272	11.06139803	3.432128	-1.335385563	down
Homo_sapiens_newGene_2393	258	55	437.978	110.858	-2.124151977	down
Homo_sapiens_newGene_2406	230	56	3.328982	0.852667228	-1.935630862	down
Homo_sapiens_newGene_2437	398	1197	4.961927824	15.03730509	1.631431942	up
Homo_sapiens_newGene_2505	23	76	2.0173679	4.903966	1.654842886	up
Homo_sapiens_newGene_2601	793	178	8.55135	1.993883	-2.088143891	down
Homo_sapiens_newGene_2874	62	161	0.4177508	1.149855	1.385488085	up
Homo_sapiens_newGene_2953	152	41	0.864554	0.2354878	-1.772079456	down
Homo_sapiens_newGene_3219	522	104	11.19565572	1.75508278	-2.247028996	down
Homo_sapiens_newGene_3225	28	1	0.523607	0.0362854	-2.969716801	down
Homo_sapiens_newGene_3229	102	13	1.33315	0.173926	-2.680143058	down
Homo_sapiens_newGene_3393	2	29	0.047234508	0.815365662	2.745571256	up
Homo_sapiens_newGene_3579	10	80	0.1762992	0.785032	2.731258234	up
Homo_sapiens_newGene_3642	0	28	4.03E-06	0.400418	3.454088607	up
Homo_sapiens_newGene_3696	8	72	0.044679925	0.3752449	2.827514087	up
Homo_sapiens_newGene_3930	255	99	39.0944	15.6047	-1.290994634	down
Homo_sapiens_newGene_3944	3	40	0.132765736	1.775584059	2.906613254	up
Homo_sapiens_newGene_4064	36	111	0.3879087	1.161816862	1.598389432	up
Homo_sapiens_newGene_4138	4	99	0.042240019	0.943489562	3.930935947	up
Homo_sapiens_newGene_4229	31	148	0.393909757	2.360022859	2.202917047	up
Homo_sapiens_newGene_4242	15	73	0.2689154	1.302753	2.131583893	up
Homo_sapiens_newGene_4285	16	96	1.12904107	6.850093326	2.435464671	up
Homo_sapiens_newGene_4475	64	294	1.465081	5.658225007	2.199217965	up
Homo_sapiens_newGene_4502	125	302	0.841653	1.943699	1.302758455	up
Homo_sapiens_newGene_4698	171	502	5.326980855	13.123509	1.587375771	up
Homo_sapiens_newGene_4741	173	36	1.901457	0.42235	-2.130311793	down
Homo_sapiens_newGene_4980	41	116	0.532316046	1.540596817	1.486011293	up
Homo_sapiens_newGene_4999	48	7	0.887356438	0.149186688	-2.325451957	down
Homo_sapiens_newGene_5580	262	760	62.988	194.1141	1.57579775	up
Homo_sapiens_newGene_5660	134	510	1.02444	3.98089	1.955171491	up
Homo_sapiens_newGene_5661	6	82	0.118782	1.56668	3.300652129	up
Homo_sapiens_newGene_580	19	93	0.4601611	2.325493	2.178549923	up
Homo_sapiens_newGene_5952	6	42	0.0906209	0.63865	2.380260218	up
Homo_sapiens_newGene_6214	89	284	8.949841	29.055	1.691776947	up
Homo_sapiens_newGene_6216	21	86	0.484193598	1.993624987	1.943075785	up
Homo_sapiens_newGene_6379	213	35	2.695379	0.459969502	-2.46356273	down
Homo_sapiens_newGene_6658	359	138	2.626374645	1.069776	-1.311892141	down
Homo_sapiens_newGene_6703	45	161	0.317542	1.15704	1.823597851	up
Homo_sapiens_newGene_7087	3	45	0.0615489	0.920123	3.065972777	up
Homo_sapiens_newGene_7148	212	64	2.846233	0.785116	-1.635592807	down
Homo_sapiens_newGene_7159	44	0	1.04329	3.04E-06	-4.01055818	down
Homo_sapiens_newGene_7177	26	104	0.312391	1.24038	1.935375599	up
Homo_sapiens_newGene_7254	267	83	21.66078	6.54447	-1.603263019	down
Homo_sapiens_newGene_7369	15	65	0.240747	1.03058	1.970696988	up
Homo_sapiens_newGene_7747	105	293	0.835862	2.356373	1.504818637	up
Homo_sapiens_newGene_786	6	90	0.0247341	0.372135	3.430695905	up
Homo_sapiens_newGene_7860	79	402	1.657419703	2.174834	2.355105679	up
Homo_sapiens_newGene_7869	0	27	9.00E-05	0.303757	3.406488549	up
Homo_sapiens_newGene_8039	4	68	0.0721678	1.25423	3.40709058	up
Homo_sapiens_newGene_8177	46	0	0.441022	0.000433897	-4.070791478	down
Homo_sapiens_newGene_8199	6	58	0.0671081	0.649113	2.820735949	up
Homo_sapiens_newGene_8250	1298	429	73.8578	23.69526	-1.541071926	down
Homo_sapiens_newGene_8259	60	204	1.29154	4.42552	1.766918998	up
Homo_sapiens_newGene_8286	44	153	3.451528	6.64564	1.78179387	up
Homo_sapiens_newGene_8546	18	131	1.16842	8.42057	2.727876343	up
Homo_sapiens_newGene_8569	12	203	0.52120383	8.306736	3.836896186	up
Homo_sapiens_newGene_8714	53	147	0.16304258	0.476230861	1.471932278	up
Homo_sapiens_newGene_8985	3	38	0.101771741	1.116588	2.837590494	up
Homo_sapiens_newGene_9070	10	49	0.103048	0.573164	2.054698751	up
Homo_sapiens_newGene_9089	229	576	1.83182	5.5856	1.369501565	up

Homo_sapiens_newGene_9102	27	902	0.143120328	6.00950019	4.96886827	up
Homo_sapiens_newGene_9107	147	48	2.617688	0.871054168	-1.510900214	down
Homo_sapiens_newGene_9148	20	101	0.169935	0.847326	2.229859087	up
Homo_sapiens_newGene_930	87	225	0.802438	2.1313	1.391254633	up
Homo_sapiens_newGene_9341	21	72	0.23333	0.792896	1.695589136	up
Homo_sapiens_newGene_9383	0	39	0.000102428	0.465655	3.894520325	up
Homo_sapiens_newGene_959	37	247	0.348915	2.38594	2.695974645	up
Homo_sapiens_newGene_9891	57	151	1.696518058	4.031556494	1.410257718	up

Supplementary Table 2. Primers for qRT-PCR

Gene name	Primers sequences	
	Forward (5'-3')	Reverse (5'-3')
Eno2	CCGGGAACTCAGACCTCATC	CTCTGCACCTAGTCGCATGG
Eno3	TATCGCAATGGGAAGTACGATCT	AAGCTCTTATACAGCTCTCCGA
Aldoa	ATGCCCTACCAATATCCAGCA	CCAGGCATTCGGCAATGTG
Hk2	GAGCCACCACTCACCCTACT	GAGCCACCACTCACCCTACT
Pgk1	TGGACGTTAAAGGGAAGCGG	GCTCATAAGGACTACCGACTTGG
Ldha	TTGACCTACGTGGCTTGGAAG	GGTAACGGAATCGGGCTGAAT
PKM	AAGAACTTGTGCGAGCCTCA	TTCCGGGTCACAGCAATGAT
RBP1	CTGCGAAGATAGCTCGCTGA	ACTTGGCACTGACATCGGTT
BOP1	CTTGATGACGAGGGCCACAG	ACGTGGGGGAAGTCATCGTA
CISD3	GTGGTTCCCTAGAACCCCAG	GTGCGTTGGAAGAAGTGGGA
CYB5R3	CCCAGCTCAGCACGTTGG	CGGGTGTCATGGCTGATGAT
DHODH	GGCTTAATGACGGAAGGAGC	CCATCAGGTAGGAGGCGAAG
G6PC3	CTTCGTTGCCTGGACTCTGG	GGATCGCCCAGAAAGGTGAT
HMMR	CGGTAGGGAGTGATAATCCGC	TCATAAGCACCTGGAGATGGTG
KISS1R	TTCATGTGCAAGTTCGTCAAC	CACACTCATGGCGGTCAG
MAT2A	CACACCGACACCAACATGAA	GCATCAAGGACAGCATCACTG
MRPL35	TGGCCTTTGGGTGAGGAGAA	AAGGATCATCAACGTACCAGTTT
MRPL40	CAGCGAGCGTCATTGTTGTC	AGCCTTTTCCAGTTTTCGGA
MRPS14	GAATGTGGCGCGATGTGAAG	GGGACGTCATAACACACCGA
MRPS28	ACAGATGGGACCTGCAAAGG	TCTAATAGCCGCAACCGGAC
MSTO1	AAGCCAATCGGCTAGGAGC	CAGCATCCTGCTGGTTCCAC
NDUFAF3	CGCGGCTTCATGATAAACGG	ACCACGATCTCTATCCGGGG
UCK2	CGGCGAGCCCTTCCTTATAG	TCTGCTCCGAGGTAAGGACA
UCP2	TGCCTACAAGACCATTGCCC	GAAGTGGCAAGGGAGGTCAT
TSFM	GTTGGTCCAGCAAGTAGCCC	AGCACCAGCTTGTGAAGTGA
SREBF1	ATGGACGAGCCACCCTTCAG	CAATCCATGGCTCCGCGAT
ETV4	GGACTTCGCCTACGACTCAG	CGCAGAGGTTTCTCATAGCCA
MEF2C	ACAAAGCCCTCAGCAGGTG	AGCACTTGCACAGCTCAGTT
RUNX1	TCTTCACAAACCCACCGCAA	CTGCCGATGTCTTCGAGGTTC
RUNX2	CTCCCTGAACTCTGCACCAA	TAGAGTGGATGGACGGGGAC
FOXA1	GAAGGGCATGAAACCAGCGA	GGTCATGTTGCCGCTCGTAG

BHLHE40	TGCCCTGCAGAGTGGTTTAC	CTCGTGCTTGGCCAGATACT
ETS1	GTGAGGTGCTGAGAGCAGAAT	TCCCAAAAGGGGTAGCAAGG
EHF	CACTCTGATGAGGGCTCAGAC	TGCCGGGGTTGAGATTCATT
NR4A2	GCACTTCGGCAGAGTTGAATG	AGCTTCAGCCGAGTTACAGG
ATF3	CCTCTGCGCTGGAATCAGTC	TTCTTTCTCGTCGCCTCTTTTT
NFIL3	AGAACAAACTAATTGCACTGGGA	GCTCGTCCACAAATGAACTCAC
JUN	TCCAAGTGCCGAAAAAGGAAG	CGAGTTCTGAGCTTTCAAGGT
EGR3	GACATCGGTCTGACCAACGAG	GGCGAACTTTCCCAAGTAGGT

Supplementary Table 3. List of Small Interference RNA Sequences

**Gene name**	**siRNA sense sequences** (5'-3')
RUNX1	CCAGGUUGCAAGAUUUAAU
FOXA1	GCACUGCAAUACUCGCCUU
BHLHE40	AUGUGAAAGCACUAACAAA
ETS1	UGGAAUUACUCACUGAUAA
NR4A2	GCCACCUUGCUUGUACCAA
ATF3	UCAUCGGCCCACGUGUAUU
NFIL3	UGCAAUAGAGCAGUCCAUU
JUN	GGGUGCCAACUCAUGCUAA
EGR3	GCAACAAGACCGUGACCUA
MAFF	GCAAAGCUCUAAAGAUCAA

Supplementary Table 4. Primers for ChIP

Name	Primers sequences		RUNX1 binding site
	Forward (5'-3')	Reverse (5'-3')	
Region 1	gggcctgcttgatggtca<br>-894 to -877	catttgtctttatactgggaaca<br>-749 to -727	Site 1
Region 2	catttaccctgatttaatcattacaca<br>-707 to -681	acgatcctgatgctgctgc<br>-527 to -509	Site 2, 3
Region 3	agatcgggagaccaggcg<br>-428 to -411	caccctccgtgaggagcc<br>-249 to -232	Site 4, 5, 6
Region 4	ggacggtaaaccctccagata<br>+377 to +397	ccctcactgaagtcaaccca<br>+485 to +504	Site 7
Region 5	gggatttccatctttctcagtca<br>+503 to +525	gactaaactgaatttcatgggcaa<br>+656 to +679	Site 8
Region 6	ctacaccttttagcaccctcattat<br>+831 to +855	aaactttcattgcaaccctgag<br>+1010 to +1031	Site 9

STR Genotyping Report of Human Cell Line

To make our science more reliable !

**Sample Submmited By** ：	Zhou Jing
**Organization** ：	ChongQing Medical University
**Sample Description** ：	HCT116
**STR Testing Corporation** ：	Feiouer （www.feiouer.com）
**Report Date** ：	2019/04/10

**Technician** ：	**No. 05**
**Quality Inspector**：	**No. 02**
**Auditor** ：	**No. 01**

Report of Human Cell Line Authentication

Analysis Date: 2019/04/10

Methods and Procedures

1. DNA is amplified with STR Multi-amplification Kit (Fluorescence Detection Kit);
2. PCR products are assayed with DNA Analyzer (Applied Biosystems®);
3. Appropriate positive and negative controls were run and confirmed for each sample submitted.
4. Comparision of the sample STR loci with an International Database is shown.

Results

1. The STR profiles of the sample are shown in Figure 1.
2. The comparision information is shown in Figure 2.
3. Conclusions are made according to the articles shown below:
   - A Resource for Cell Line Authentication, Annotation and Quality Control, **Nature**, 520(2015), 307-11.
   - Authentication of Human Cell Lines by Str DNA Profiling Analysis', (2013).

Conclusions

① **Obvious contamination of other human cell line is not found.**

② **According to the comparison information, the sample matches with a human cell line which is named as** “HCT116” **.**

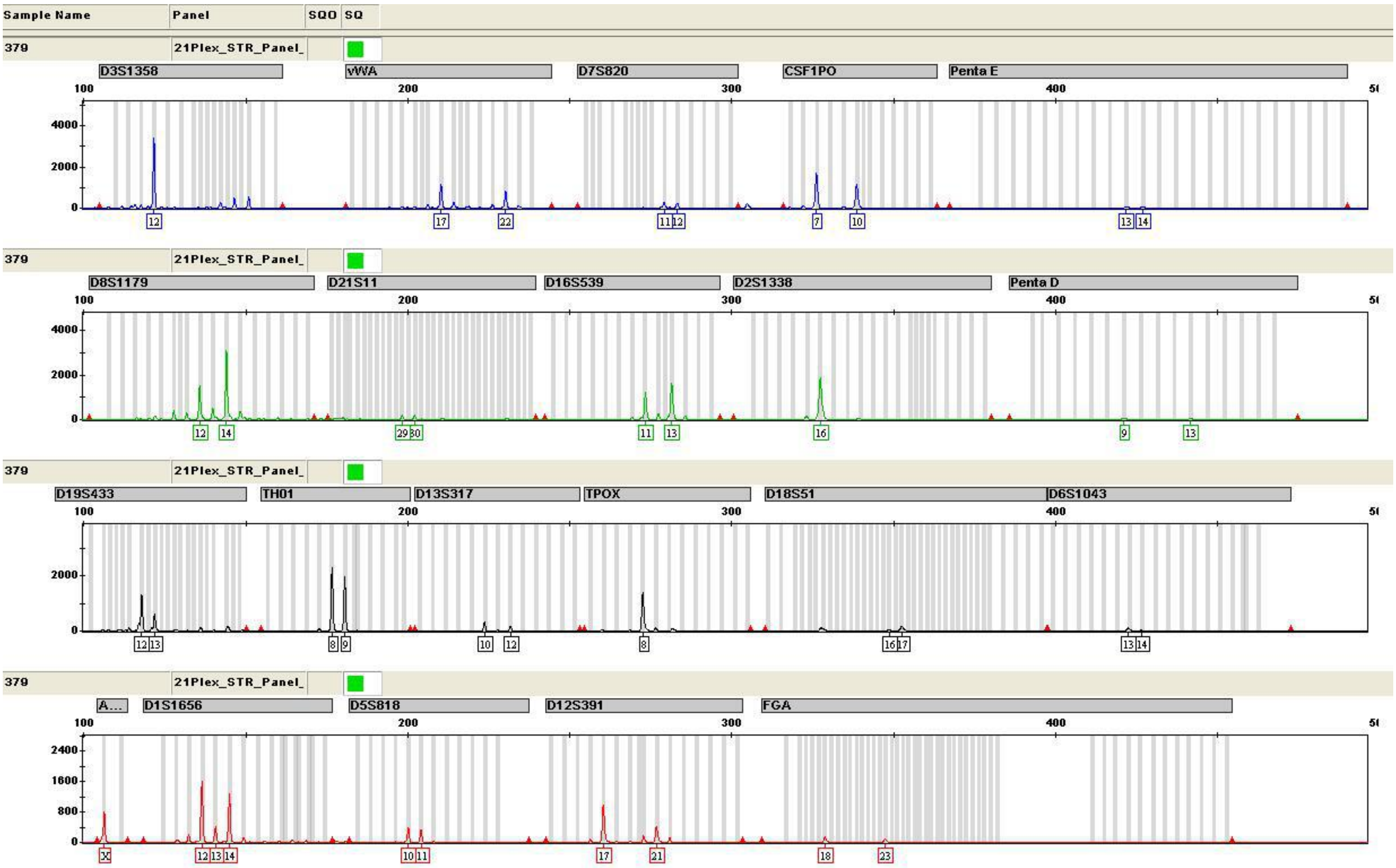

Figure 1. STR profiles of the sample

**Result of STR matching analysis by your data.** - DSMZ Profile Database -												
A graphical presentation is shown at the bottom of this page.												
EV	Cell No.	Cell name	Locus names									Figures
			D5S818	D13S317	D7S820	D16S539	VWA	TH01	AM	TPOX	CSF1PO	
	Query (Your Cell)		10,11	10,12	11,12	11,13	17,22	8,9	X,X	8,8	7,10	
0.89(32/36)	CCL-247	HCT 116	10,11	10,12	11,12	11,13	17,22	8,9	X,Y	8,9	7,10	-

Figure 2. Comparision of the sample STR loci with an International Database

References:

[1] M. Yu, S. K. Selvaraj, M. M. Liang-Chu, S. Aghajani, M. Busse, J. Yuan, G. Lee, F. Peale, C. Klijn, R. Bourgon, J. S. Kaminker, and R. M. Neve, 'A Resource for Cell Line Authentication, Annotation and Quality Control', **Nature**, 520 (2015), 307-11.

[2] Yvonne Reid, PhD,1 Douglas Storts, PhD,2 Terry Riss, PhD,3,* and Lisa Minor,PhD4,*Authentication of Human Cell Lines by Str DNA Profiling Analysis', (2013).

[3] John Butler, 'Short Tandem Repeat Typing Technologies Used in Human Identity Testing', BioTechniques, 43 (2007), Sii-Sv.

[4] Hao Fan, and Jia-You Chu, 'A Brief Review of Short Tandem Repeat Mutation', Genomics, Proteomics & Bioinformatics, 5 (2007), 7-14.

[5] L. P. Freedman, M. C. Gibson, S. P. Ethier, H. R. Soule, R. M. Neve, and Y. A. Reid, 'Reproducibility: Changing the Policies and Culture of Cell Line Authentication', Nat Methods, 12 (2015), 493-7.

[6] W. Parson, R. Kirchebner, R. Muhlmann, K. Renner, A. Kofler, S. Schmidt, and R. Kofler, 'Cancer Cell Line Identification by Short Tandem Repeat Profiling: Power and Limitations', FASEB J, 19 (2005), 434-6.

[7] R. Wooster, A. M. Cleton-Jansen, N. Collins, J. Mangion, R. S. Cornelis, C. S. Cooper, B. A. Gusterson, B. A. Ponder, A. von Deimling, O. D. Wiestler, and et al., 'Instability of Short Tandem Repeats (Microsatellites) in Human Cancers', Nat Genet, 6 (1994), 152-6.

[8] Jaiprakash G. Shewale, 'Str Profiling of Human Cell Lines: Challenges and Possible Solutions to the Growing Problem', Journal of Forensic Research, s2 (2011).

细 胞 系 支 原 体 检 测 报 告

Mycoplasma Contamination Testing of a Cell Line

送检单位：重庆医科大学

送检人：周静

送检样品名：HCT116

委托单位：成都飞鸥尔生物（www.feiouer.com）

报告日期：2019/04/10

**技术人员：**	**No. 07**
**质控人员：**	**No. 03**
**审核人员：**	**No. 02**

Mycoplasma Contamination Testing of a Cell Line

Analysis Date: 2019/04/10

Important Materials

1. Wizard® Genomic DNA Purification Kit
2. CFX96 Touch™ Real-Time PCR Detection System | Life Science
3. 2× MycoSensor QPCR Master Mix (containing *SYBR Green I dye*)
4. MycoSensor Primer Mix
5. A. laidlawii Positive Control
6. Nuclease-free PCR-grade water

Important Methods and Procedures

1. DNA extraction is accomplished by using Wizard® Genomic DNA Purification Kit;
2. Quantitative PCR is conducted as shown below:

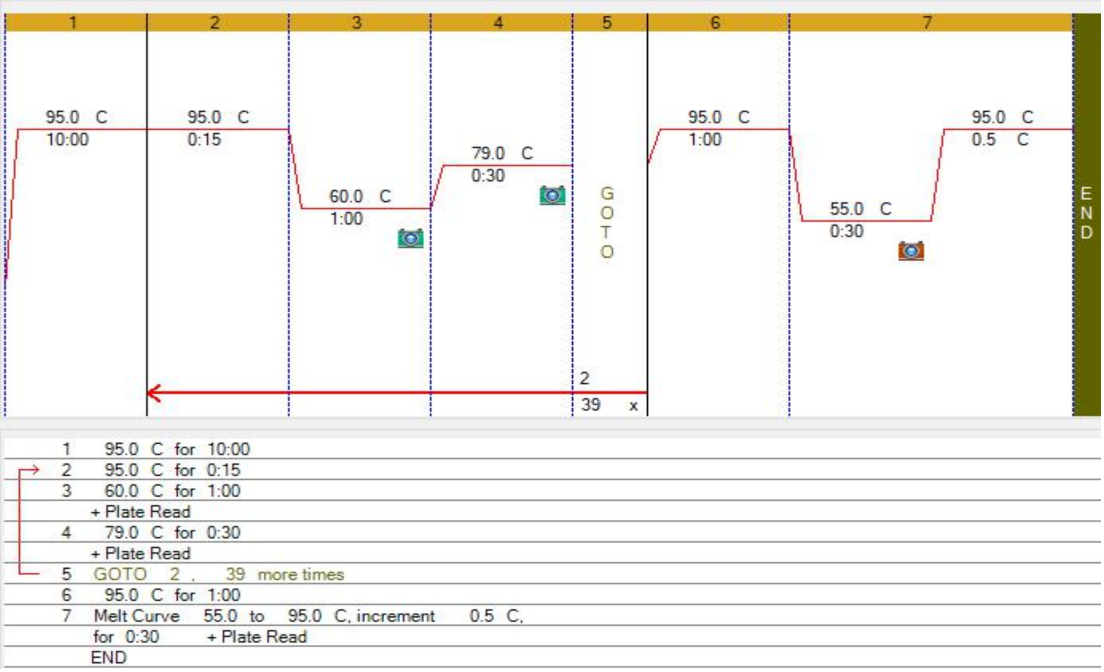

3. Appropriate positive and negative controls were run and confirmed for each sample submitted;
4. All experiments were conducted in duplicate;
5. Data is analysed with Bio-Rad CFX Manager (Version 3.1);

Results

1. The Amplification Chart is shown in Figure 1.
2. The Melt Peaks Chart is shown in Figure 2.
3. The Cq and Melt Temp values are shown in Figure 3.

Conclusion

According to all the Figures , the positive control (A. laidlawii) is confirmed by our detection system. In comparison , the Sample's Cq value and Melt Temperature is not available ,which indicates that the Sample is **NOT** contaminated by mycoplasma.

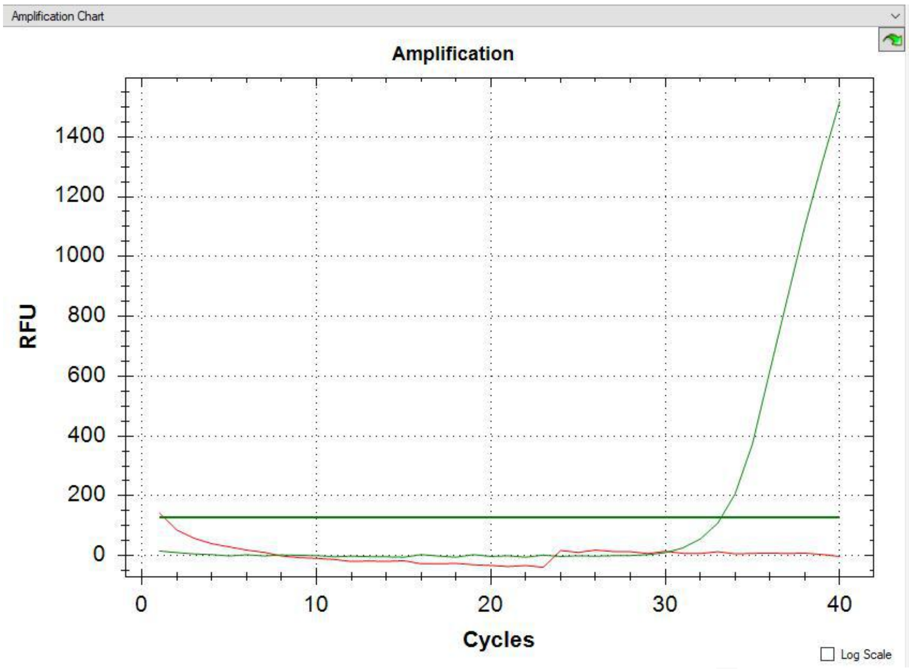

Figure 1. Amplification Chart of the Sample (Red) and the Positive Control(Green)

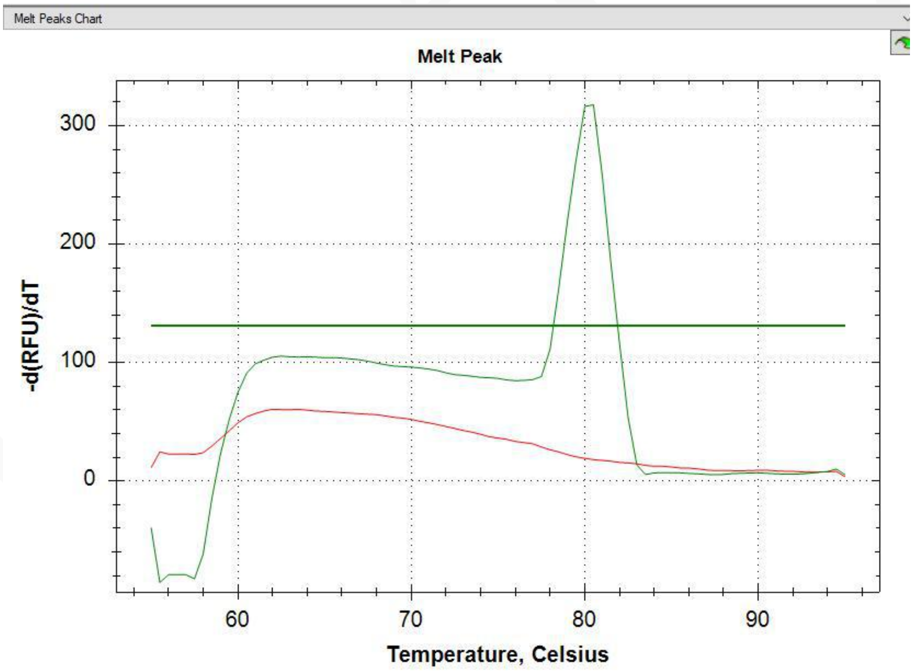

Figure 2. Melt Peaks Chart of the Sample (Red) and the Positive Control(Green)

	Cq	Melt Temp.
Positive Control	33. 2	≈80. 0
Sample	N/A	None

Figure 3. Cq and Melt Temperature values

References:

1. Hellebuyck T, Gobel S, Pasmans F, Adriaensen C, Martel A. Co-occurrence of Mycoplasma Species and Pigeon Herpesvirus-1 Infection in Racing Pigeons (Columba livia). Journal of avian medicine and surgery 2017; 31(4): 351-5.

2. Poumarat F, Gautier-Bouchardon AV, Bergonier D, Gay E, Tardy F. Diversity and variation in antimicrobial susceptibility patterns over time in Mycoplasma agalactiae isolates collected from sheep and goats in France. Journal of applied microbiology 2016; 120(5): 1208-18

3. Iramain R, De Jesus R, Spitters C, et al. Chlamydia pneumoniae, and mycoplasma pneumoniae: Are they related to severe asthma in childhood? The Journal of asthma : official journal of the Association for the Care of Asthma 2016; 53(6): 618-21.

4. Duenas Meza E, Jaramillo CA, Correa E, et al. Virus and Mycoplasma pneumoniae prevalence in a selected pediatric population with acute asthma exacerbation. The Journal of asthma : official journal of the Association for the Care of Asthma 2016; 53(3): 253-60.

5. Sanna G, Lecca V, Foddai A, Tola S. Development of a specific immunomagnetic capture-PCR for rapid detection of viable Mycoplasma agalactiae in sheep milk samples. Journal of applied microbiology 2014; 117(6): 1585-91.

6. Hellebuyck T, Garmyn A, De Cooman L, Boyen F, Pasmans F, Martel A. Mycoplasma columbinum Isolated From a Racing Pigeon (Columba livia) With Arthritis. Journal of avian medicine and surgery 2014; 28(3): 240-1.

7. Bezjian M, Bezjian M. American kestrel (Falco spaverius) fledgling with severe bilateral periorbital swelling and infection with Mycoplasma buteonis, Avibacterium (Pasteurella) gallinarum, and Staphylococcus pasteuri. Journal of avian medicine and surgery 2014; 28(2): 127-31.

8. Adan-Kubo J, Yoshii SH, Kono H, Miyata M. Molecular structure of isolated MvspI, a variable surface protein of the fish pathogen Mycoplasma mobile. Journal of bacteriology 2012; 194(12): 3050-7.

9. Peredeltchouk M, David SA, Bhattacharya B, Volokhov DV, Chizhikov V. Detection of mycoplasma contamination in cell substrates using reverse transcription-PCR assays. Journal of applied microbiology 2011; 110(1): 54-60.

10. Tang LF, Shi YC, Xu YC, Wang CF, Yu ZS, Chen ZM. The change of asthma-associated immunological parameters in children with Mycoplasma pneumoniae infection. The Journal of asthma : official journal of the Association for the Care of Asthma 2009; 46(3): 265-9.

11. Basu S, Saifudeen A, Kaur P. Transient cold agglutinin disease with mycoplasma infection. The Journal of the Association of Physicians of India 2009; 57: 656-7.

12. Imudia AN, Detti L, Puscheck EE, Yelian FD, Diamond MP. The prevalence of ureaplasma urealyticum, mycoplasma hominis, chlamydia trachomatis and neisseria gonorrhoeae infections, and the rubella status of patients undergoing an initial infertility evaluation. Journal of assisted reproduction and genetics 2008; 25(1): 43-6.

13. Adan-Kubo J, Uenoyama A, Arata T, Miyata M. Morphology of isolated Gli349, a leg protein responsible for Mycoplasma mobile gliding via glass binding, revealed by rotary shadowing electron microscopy. Journal of bacteriology 2006; 188(8): 2821-8.

STR Genotyping Report of Human Cell Line

To make our science more reliable !

**Sample Submmited By** ：	Zhou Jing
**Organization** ：	ChongQing Medical University
**Sample Description** ：	SW480
**STR Testing Corporation** ：	Feiouer （www.feiouer.com）
**Report Date** ：	2019/05/02

**Technician：**	**No. 05**
**Quality Inspector:**	**No. 02**
**Auditor：**	**No. 01**

Report of Human Cell Line Authentication

Analysis Date: 2019/05/02

Methods and Procedures

1. DNA is amplified with STR Multi-amplification Kit (Fluorescence Detection Kit);
2. PCR products are assayed with DNA Analyzer (Applied Biosystems®);
3. Appropriate positive and negative controls were run and confirmed for each sample submitted.
4. Comparision of the sample STR loci with an International Database is shown.

Results

1. The STR profiles of the sample are shown in Figure 1.
2. The comparision information is shown in Figure 2.
3. Conclusions are made according to the articles shown below:
   - A Resource for Cell Line Authentication, Annotation and Quality Control, **Nature**, 520(2015), 307-11.
   - Authentication of Human Cell Lines by Str DNA Profiling Analysis', (2013).

Conclusions

① **Obvious contamination of other human cell line is not found.**

② **According to the comparison information, the sample matches with a human cell line which is named as** “SW480” **.**

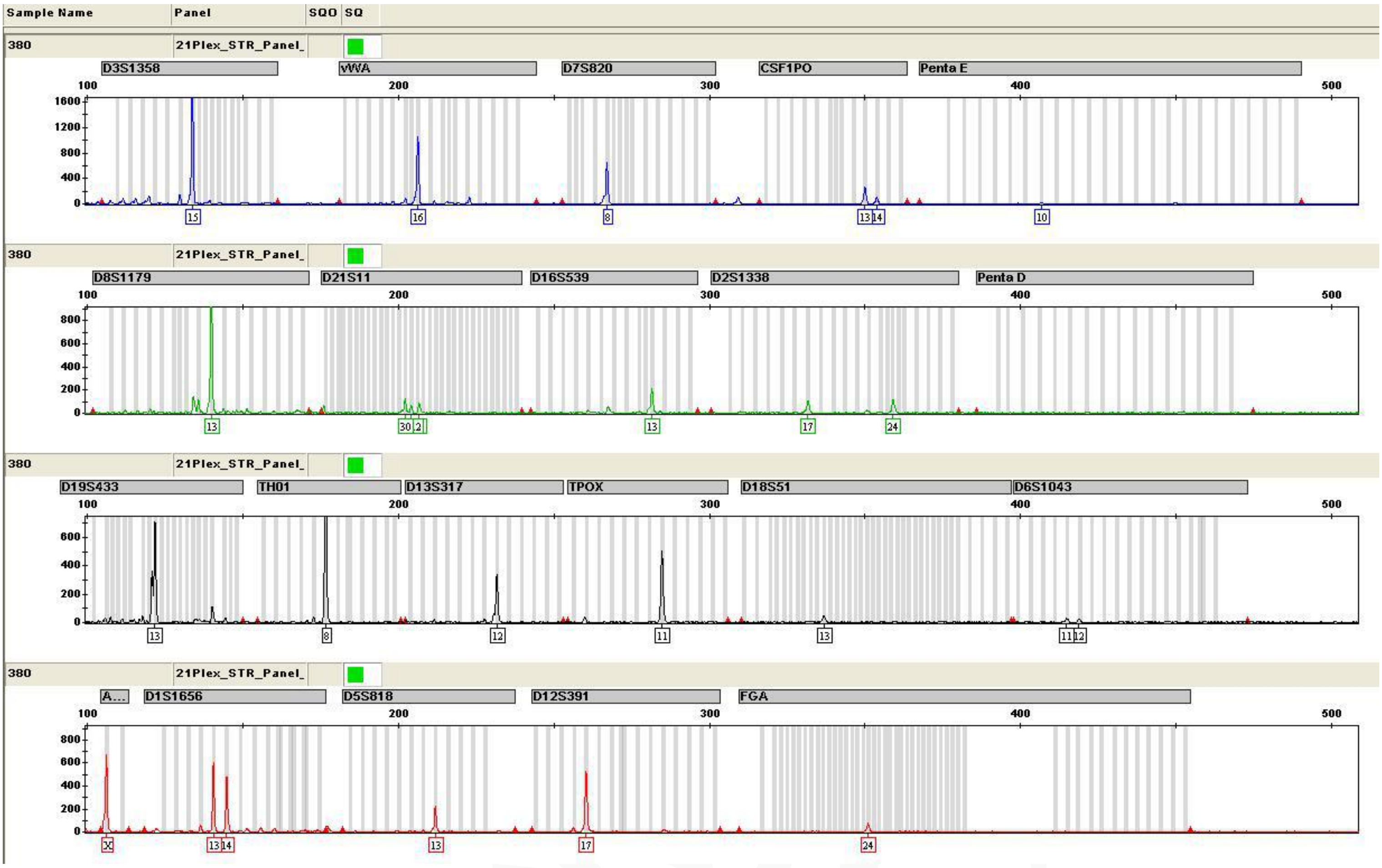

Figure 1. STR profiles of the sample

Result of STR matching analysis by your data.

- DSMZ Profile Database -

A graphical presentation is shown at the bottom of this page.

EV	Cell No.	Cell name	Locus names									Figures
			D5S818	D13S317	D7S820	D16S539	VWA	TH01	AM	TPOX	CSF1PO	
	Query (Your Cell)		13,13	12,12	8,8	13,13	16,16	8,8	X,X	11,11	13,14	
1.00(36/36)	313	SW-480	13,13	12,12	8,8	13,13	16,16	8,8	X,X	11,11	13,14	-

Figure 2. Comparision of the sample STR loci with an International Database

References:

[1] M. Yu, S. K. Selvaraj, M. M. Liang-Chu, S. Aghajani, M. Busse, J. Yuan, G. Lee, F. Peale, C. Klijn, R. Bourgon, J. S. Kaminker, and R. M. Neve, 'A Resource for Cell Line Authentication, Annotation and Quality Control', **Nature**, 520 (2015), 307-11.

[2] Yvonne Reid, PhD,1 Douglas Storts, PhD,2 Terry Riss, PhD,3,* and Lisa Minor,PhD4,*Authentication of Human Cell Lines by Str DNA Profiling Analysis', (2013).

[3] John Butler, 'Short Tandem Repeat Typing Technologies Used in Human Identity Testing', BioTechniques, 43 (2007), Sii-Sv.

[4] Hao Fan, and Jia-You Chu, 'A Brief Review of Short Tandem Repeat Mutation', Genomics, Proteomics & Bioinformatics, 5 (2007), 7-14.

[5] L. P. Freedman, M. C. Gibson, S. P. Ethier, H. R. Soule, R. M. Neve, and Y. A. Reid, 'Reproducibility: Changing the Policies and Culture of Cell Line Authentication', Nat Methods, 12 (2015), 493-7.

[6] W. Parson, R. Kirchebner, R. Muhlmann, K. Renner, A. Kofler, S. Schmidt, and R. Kofler, 'Cancer Cell Line Identification by Short Tandem Repeat Profiling: Power and Limitations', FASEB J, 19 (2005), 434-6.

[7] R. Wooster, A. M. Cleton-Jansen, N. Collins, J. Mangion, R. S. Cornelis, C. S. Cooper, B. A. Gusterson, B. A. Ponder, A. von Deimling, O. D. Wiestler, and et al., 'Instability of Short Tandem Repeats (Microsatellites) in Human Cancers', Nat Genet, 6 (1994), 152-6.

[8] Jaiprakash G. Shewale, 'Str Profiling of Human Cell Lines: Challenges and Possible Solutions to the Growing Problem', Journal of Forensic Research, s2 (2011).

细 胞 系 支 原 体 检 测 报 告

Mycoplasma Contamination Testing of a Cell Line

送检单位：重庆医科大学基础医学院

送检人：周静

送检样品名：SW480

委托单位：成都飞鸥尔生物（www.feiouer.com）

报告日期：2019/05/02

**技术人员：**	**No. 07**
**质控人员：**	**No. 03**
**审核人员：**	**No. 02**

Mycoplasma Contamination Testing of a Cell Line

Analysis Date: 2019/05/02

Important Materials

1. Wizard® Genomic DNA Purification Kit
2. CFX96 Touch™ Real-Time PCR Detection System | Life Science
3. 2× MycoSensor QPCR Master Mix (containing *SYBR Green I dye*)
4. MycoSensor Primer Mix
5. A. laidlawii Positive Control
6. Nuclease-free PCR-grade water

Important Methods and Procedures

1. DNA extraction is accomplished by using Wizard® Genomic DNA Purification Kit;
2. Quantitative PCR is conducted as shown below:

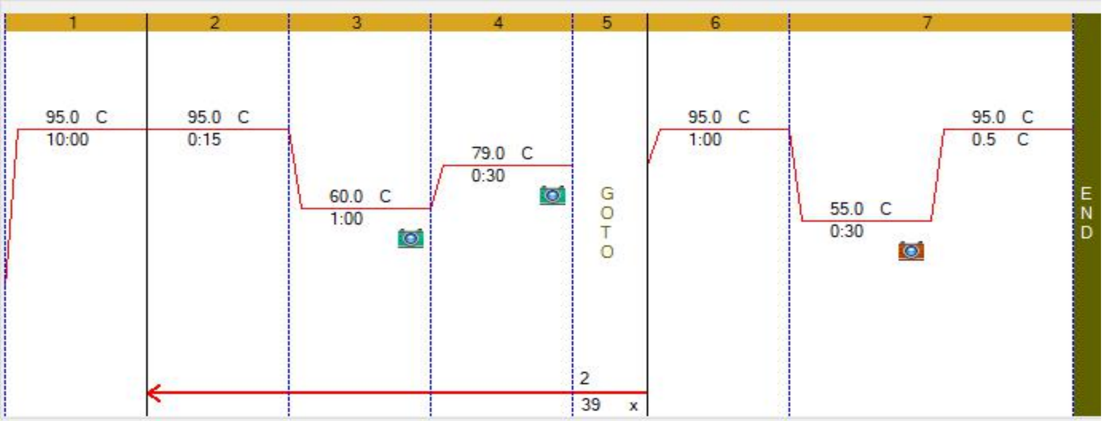


```
1    95.0  C  for  10:00
2    95.0  C  for  0:15
3    60.0  C  for  1:00
   + Plate Read
4    79.0  C  for  0:30
   + Plate Read
5   GOTO   2 ,    39  more times
6    95.0  C  for  1:00
7   Melt Curve   55.0  to   95.0  C, increment   0.5  C,
    for  0:30        + Plate Read
    END
```

3. Appropriate positive and negative controls were run and confirmed for each sample submitted;
4. All experiments were conducted in duplicate;
5. Data is analysed with Bio-Rad CFX Manager (Version 3.1);

Results

1. The Amplification Chart is shown in Figure 1.
2. The Melt Peaks Chart is shown in Figure 2.
3. The Cq and Melt Temp values are shown in Figure 3.

Conclusion

According to all the Figures , the positive control (A. laidlawii) is confirmed by our detection system. In comparison , the Sample's Cq value and Melt Temperature is not available ,which indicates that the Sample is **NOT** contaminated by mycoplasma.

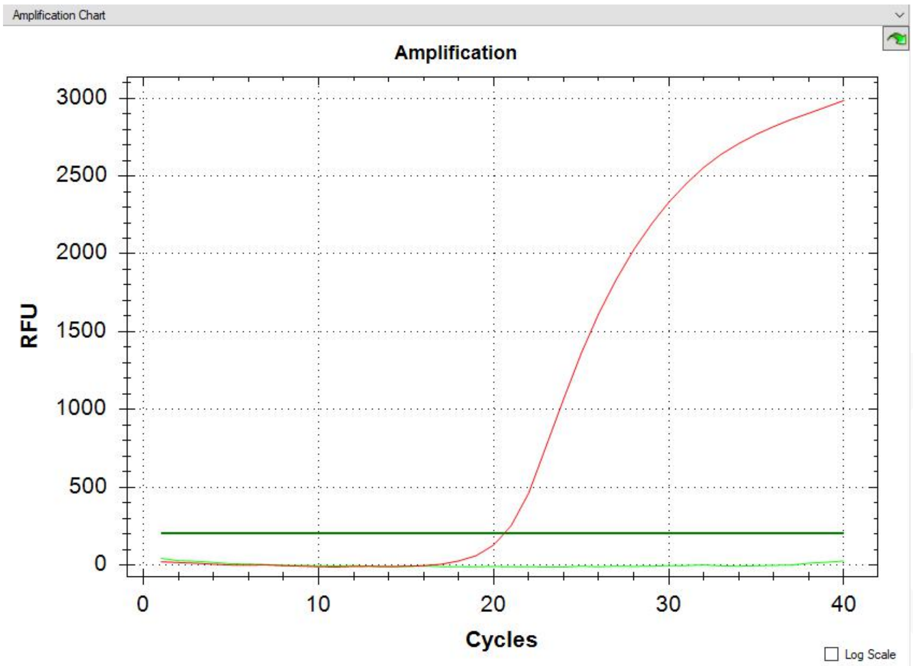

Figure 1. Amplification Chart of the Sample (Green) and the Positive Control(Red)

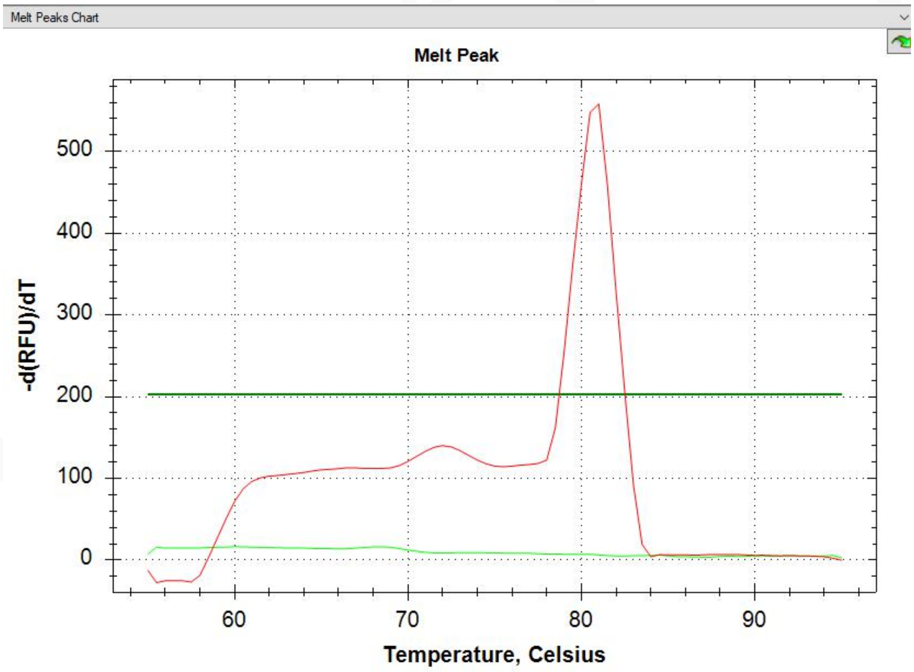

Figure 2. Melt Peaks Chart of the Sample (Green) and the Positive Control(Red)

	Cq	Melt Temp.
Positive Control	20. 59	≈80. 0
Sample	N/A	None

Figure 3. Cq and Melt Temperature values

References:

1. Hellebuyck T, Gobel S, Pasmans F, Adriaensen C, Martel A. Co-occurrence of Mycoplasma Species and Pigeon Herpesvirus-1 Infection in Racing Pigeons (Columba livia). Journal of avian medicine and surgery 2017; 31(4): 351-5.

2. Poumarat F, Gautier-Bouchardon AV, Bergonier D, Gay E, Tardy F. Diversity and variation in antimicrobial susceptibility patterns over time in Mycoplasma agalactiae isolates collected from sheep and goats in France. Journal of applied microbiology 2016; 120(5): 1208-18

3. Iramain R, De Jesus R, Spitters C, et al. Chlamydia pneumoniae, and mycoplasma pneumoniae: Are they related to severe asthma in childhood? The Journal of asthma : official journal of the Association for the Care of Asthma 2016; 53(6): 618-21.

4. Duenas Meza E, Jaramillo CA, Correa E, et al. Virus and Mycoplasma pneumoniae prevalence in a selected pediatric population with acute asthma exacerbation. The Journal of asthma : official journal of the Association for the Care of Asthma 2016; 53(3): 253-60.

5. Sanna G, Lecca V, Foddai A, Tola S. Development of a specific immunomagnetic capture-PCR for rapid detection of viable Mycoplasma agalactiae in sheep milk samples. Journal of applied microbiology 2014; 117(6): 1585-91.

6. Hellebuyck T, Garmyn A, De Cooman L, Boyen F, Pasmans F, Martel A. Mycoplasma columbinum Isolated From a Racing Pigeon (Columba livia) With Arthritis. Journal of avian medicine and surgery 2014; 28(3): 240-1.

7. Bezjian M, Bezjian M. American kestrel (Falco spaverius) fledgling with severe bilateral periorbital swelling and infection with Mycoplasma buteonis, Avibacterium (Pasteurella) gallinarum, and Staphylococcus pasteuri. Journal of avian medicine and surgery 2014; 28(2): 127-31.

8. Adan-Kubo J, Yoshii SH, Kono H, Miyata M. Molecular structure of isolated MvspI, a variable surface protein of the fish pathogen Mycoplasma mobile. Journal of bacteriology 2012; 194(12): 3050-7.

9. Peredeltchouk M, David SA, Bhattacharya B, Volokhov DV, Chizhikov V. Detection of mycoplasma contamination in cell substrates using reverse transcription-PCR assays. Journal of applied microbiology 2011; 110(1): 54-60.

10. Tang LF, Shi YC, Xu YC, Wang CF, Yu ZS, Chen ZM. The change of asthma-associated immunological parameters in children with Mycoplasma pneumoniae infection. The Journal of asthma : official journal of the Association for the Care of Asthma 2009; 46(3): 265-9.

11. Basu S, Saifudeen A, Kaur P. Transient cold agglutinin disease with mycoplasma infection. The Journal of the Association of Physicians of India 2009; 57: 656-7.

12. Imudia AN, Detti L, Puscheck EE, Yelian FD, Diamond MP. The prevalence of ureaplasma urealyticum, mycoplasma hominis, chlamydia trachomatis and neisseria gonorrhoeae infections, and the rubella status of patients undergoing an initial infertility evaluation. Journal of assisted reproduction and genetics 2008; 25(1): 43-6.

13. Adan-Kubo J, Uenoyama A, Arata T, Miyata M. Morphology of isolated Gli349, a leg protein responsible for Mycoplasma mobile gliding via glass binding, revealed by rotary shadowing electron microscopy. Journal of bacteriology 2006; 188(8): 2821-8.
